# A lipidomics platform to analyze the fatty acid compositions of non-polar and polar lipid molecular species from plant tissues: Examples from developing seeds and seedlings of pennycress (*Thlaspi arvense*)

**DOI:** 10.3389/fpls.2022.1038161

**Published:** 2022-11-09

**Authors:** Trevor B. Romsdahl, Jean-Christophe Cocuron, Mackenzie J. Pearson, Ana Paula Alonso, Kent D. Chapman

**Affiliations:** ^1^ Mass Spectrometry Facility, Department of Biochemistry and Molecular Biology, University of Texas Medical Branch, Galveston, TX, United States; ^2^ Department of Biological Sciences & BioDiscovery Institute, University of North Texas, Denton, TX, United States; ^3^ BioAnalytical Facility, University of North Texas, Denton, TX, United States; ^4^ Sciex, Framingham, MA, United States

**Keywords:** *Thlaspi arvense*, lipidomics, mass spectrometry, oilseed, pennycress, LC-MS/MS

## Abstract

The lipidome comprises the total content of molecular species of each lipid class, and is measured using the analytical techniques of lipidomics. Many liquid chromatography-mass spectrometry (LC-MS) methods have previously been described to characterize the lipidome. However, many lipidomic approaches may not fully uncover the subtleties of lipid molecular species, such as the full fatty acid (FA) composition of certain lipid classes. Here, we describe a stepwise targeted lipidomics approach to characterize the polar and non-polar lipid classes using complementary LC-MS methods. Our “polar” method measures 260 molecular species across 12 polar lipid classes, and is performed using hydrophilic interaction chromatography (HILIC) on a NH2 column to separate lipid classes by their headgroup. Our “non-polar” method measures 254 molecular species across three non-polar lipid classes, separating molecular species on their FA characteristics by reverse phase (RP) chromatography on a C30 column. Five different extraction methods were compared, with an MTBE-based extraction chosen for the final lipidomics workflow. A state-of-the-art strategy to determine and relatively quantify the FA composition of triacylglycerols is also described. This lipidomics workflow was applied to developing, mature, and germinated pennycress seeds/seedlings and found unexpected changes among several lipid molecular species. During development, diacylglycerols predominantly contained long chain length FAs, which contrasted with the very long chain FAs of triacylglycerols in mature seeds. Potential metabolic explanations are discussed. The lack of very long chain fatty acids in diacylglycerols of germinating seeds may indicate very long chain FAs, such as erucic acid, are preferentially channeled into beta-oxidation for energy production.

## 1 Introduction

Lipids are essential biomolecules for life and play many roles. They are used as an energy reserve, to create cellular and organellar membranes that help to segregate compartments of the cell, to facilitate vesicular and endomembrane trafficking, to form protective and structural barriers around organisms, and as signaling molecules. Many of the phospholipid classes, such as phosphatidylcholine (PC), phosphatidylserine (PS), and phosphatidylinositol (PI), are involved with signaling and tissue differentiation (Colin and Jaillais, 2020). Lysophospholipids are usually low abundant, but can rapidly increase as signaling molecules during wounding (Okazaki and Saito, 2014). Lipids are also important for plant immunity, such as the use of polyunsaturated FAs from galactolipids of the plastids to synthesize jasmonic acid, an important signaling molecule (Cavaco et al., 2021). Triacylglycerols (TGs) are a major energy reserve that accumulate during seed development to be used when seeds germinate, but also have important economic and commercial value (Durrett et al., 2008; Dyer et al., 2008).

**Figure 1:**
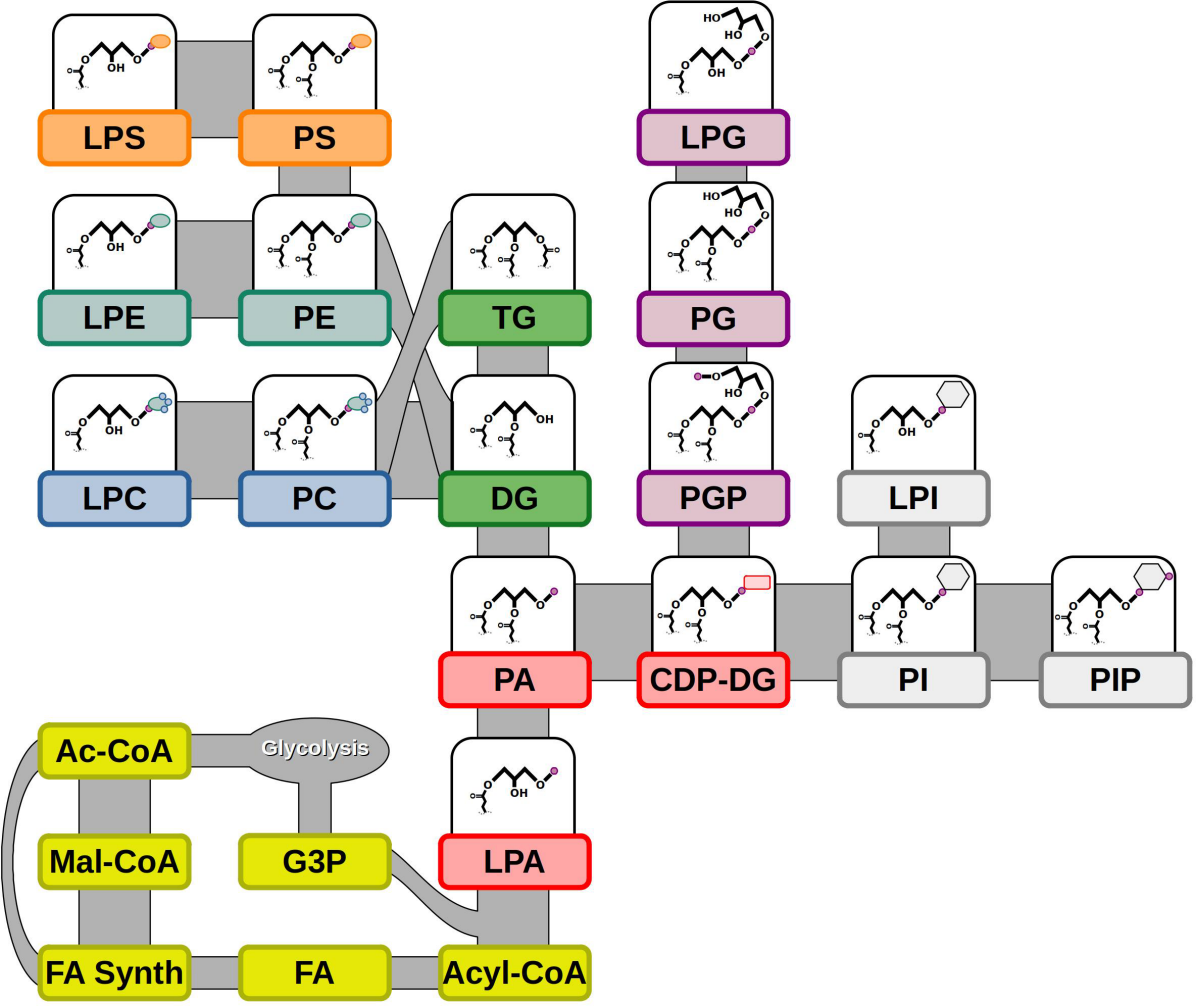
Lipid biosynthesis and molecular diversity. Lipid synthesis begins with precursors from glycolysis: Ac-CoA & G3P. FAs produced from FA synthesis are made into Acyl-CoA and added to G3P to synthesize LPA. Additional acylation and subsequent phospho headgroup addition leads to other lipid classes. Abbreviations – Ac-CoA, acetyl-Coenzyme A; CDP-DG, cytidine diphosphate diacylglycerol; DG, diacylglycerol; FA, fatty acid; FA Synth, fatty acid synthesis; G3P, glycerol-3-phosphate; LPA, lysophosphatidic acid; LPC, lysophosphatidylcholine; LPE, lysophosphatidylethanolamine; LPG, lysophosphatidylglycerol; LPI, lysophosphatidylinositol; LPS, lysophosphatidylserine; Mal-CoA, malonyl-Coenzyme A; PA, phosphatidic acid; PC, phosphatidylcholine; PE, phosphatidylethanolamine; PG, phosphatidylglycerol; PGP, phosphatidylglycerol phosphate; PI, phosphatidylinositol; PIP, phosphatidylinositol-phosphate; PS, phosphatidylserine; TG, triacylglycerol. Shapes – small purple circle, phosphate; rounded red rectangle, CDP; gray hexagon, inositol; light green oval, ethanolamine; small blue circle, methyl group; orange oval, serine.

Synthesis of lipids begins with FAs in the plastids for plants, followed by sequential acylation of a glycerol-3-phosphate to form phosphatidic acid (PA) (**Figure 1**). PA can be dephosphorylated to form the neutral lipid diacylglycerol (DG) that can lead to either TGs with an additional acylation or to other phospholipids, including PC, PE, and PS, by the addition of various phospho headgroups (Nakamura, 2021). Alternatively, PA can become activated with the addition of cytidine-5’-diphosphate (CDP) to lead to other phospholipids, including phosphatidylglycerol (PG), PI, and PS (**Figure 1**) (Jennings and Epand, 2020). Many of the phospholipids can form lyso- counterparts through the deacylation of the *sn*-2 position of the glycerol backbone (Li-Beisson et al., 2010). Lipid molecules vary widely in their chemistry and molecular structure. Different classes of lipids are categorized by their headgroup or lack thereof. Polar lipids have a phospho- or glyco-headgroups and mostly organize into bilayer structures for biomembranes, while non-polar lipids either lack a headgroup, such as DGs, or have an additional fatty acid (FA) to form TGs and do not support a bilayer configuration (Han and Gross, 2021). Further diversity in lipid classes can be seen in the wide range of FAs that are esterified to glycerol backbones of lipids, which differ in unsaturation, carbon length, or other modifications (Cahoon and Li-Beisson, 2020; He et al., 2020). The characteristics of these FAs and the composition of lipids as a whole can greatly affect their properties biologically, as well as their use for human use or consumption, such as the storage oils from seeds.

Oilseed crops are predominantly used for nutritional needs, including human consumption and cattle feed. However, seed oils have increasingly been utilized for other purposes, such as biofuels and sustainable sources of chemical feedstocks (Durrett et al., 2008; Dyer et al., 2008; Ohlrogge and Chapman, 2011). The diversification of oilseed usage, combined to the constant augmentation and modernization of the world’s population, will put more burden on oilseed production which will need enhancements to sustain the demand for both nutritional and biotechnological applications. In the last decade, scientists began working with alternative crop species, such as *Camelina sativa* and *Thlaspi arvense* (pennycress), to meet this increasing demand for seed oil. This study focused on pennycress due to several beneficial traits. First, pennycress is a diploid Brassicaceae and winter annual that grows in temperate regions of the world where it can be rotated with traditional agricultural crops, such as corn or soybean (Best and Mcintyre, 1975; Fan et al., 2013). This crop rotation cycle negates the need to displace other crops from their food and industrial uses, and makes more efficient use of limited crop land. Second, as pennycress is closely related to the well-studied model plant *Arabidopsis*, it could be used as a real-world crop with translational properties for agriculture (Sedbrook et al., 2014; Tsogtbaatar et al., 2015; Chopra et al., 2018; McGinn et al., 2019; Tsogtbaatar et al., 2020; Chopra et al., 2020; Sedbrook and Durrett, 2020; Jarvis et al., 2021; Romsdahl et al., 2021).Third, pennycress seed oil composition has a high erucic acid (22:1^Δ13^) content which makes it a suitable drop-in jet fuel additive, particularly for its performance at colder temperatures where it has good fluidity and lubrication properties (Moser et al., 2009; Moser, 2012; Fan et al., 2013). Lastly, pennycress has been shown to be amenable to genetic engineering *via* agrobacterium mediated transformation and CRISPR-Cas9 gene editing, allowing for the potential to customize FA composition or other oil properties (McGinn et al., 2019; Jarvis et al., 2021). However, such transgenic efforts require an accurate understanding of the metabolic changes in oil synthesis which can be assessed by lipidomics.

Lipidomics is the study of lipid profiles using analytical techniques, such as mass spectrometry. The lipidome constitutes the many different classes and molecular species of lipids found in cells, tissues, or other sample types. These lipid classes and species differ by their relative polarity and structure, often due to a headgroup and FA composition. Because of this wide diversity of chemical characteristics, there is not a single lipid extraction method that can completely recover the different classes of lipids from a biological sample. The most common extraction protocols used are generally the Bligh and Dyer (Bligh and Dyer, 1959) and the Folch (Folch et al., 1957) methods, both chloroform-methanol-water based extractions. Recently, other lipid extraction techniques have emerged to offer new advantages. These include the use of: i) isopropanol as a means to inhibit endogenous phospholipases (Chapman and Moore, 1993), and ii) non-toxic solvents such as butanol and methyl-tert-butyl-ether (MTBE), which induce phase separation of lipids into an upper phase to allow for cleaner sample collection prior to HPLC analysis (Matyash et al., 2008; Löfgren et al., 2016). Along with the extraction process, representative internal standards (isotopically labeled, hydrogenated, or odd chain lipids) are added for each class of lipid in order to aid with the quantification following data collection.

Two of the most common methods of lipidomics analysis using mass spectrometry include either direct infusion (“shotgun” lipidomics) or techniques coupled to liquid chromatography. Direct infusion lipidomics consists in injecting a lipid extract directly into a mass spectrometer and separating different lipid classes by a variety of precursor, neutral loss, and product ion scans using a triple quadrupole mass spectrometer or other hybrid instrument (Welti et al., 2002; Taguchi et al., 2005; Han and Gross, 2021). However, direct infusion lipidomics is unable to resolve lipid molecular species that are isomers or isobars of one another and may suffer from ion suppression effects that can lower the sensitivity of low abundant lipids (Cajka and Fiehn, 2014). In contrast, high performance liquid chromatography (HPLC) allows for the separation of lipids based on their polarities or structural properties. For instance, normal phase (NP) or HILIC chromatography separate lipid classes by their polar head group or lack thereof, while reverse phase (RP) chromatography separates those by the characteristics of their fatty acyl chains, including carbon length and unsaturation. The combination of HPLC and MS allows accurate identification and quantification of molecular species based on the chromatographic retention time and MS/MS fragmentation, and helps to minimize ion suppression effects often found in direct infusion approaches (Rustam and Reid, 2018). Along with MS/MS fragmentation, the FA composition of chromatographically separated isobaric and isomeric molecular species can be determined. However, this still remains particularly challenging for triacylglycerols (TG) which contain three FA chains. Isobaric and isomeric species of TG differ only by the subtleties of their FA composition, such as *sn*-position, double bond location, and carbon length. Recently the technology of RP chromatography has advanced to allow for greater separation of non-polar lipids using C30 columns that produce narrower peaks, better theoretical plate number and retention time reproducibility than C8 or C18 columns (Okusa et al., 2014; Narváez-Rivas and Zhang, 2016; Rampler et al., 2018; Jankevics et al., 2021). Using C30 columns in conjunction with specific MS scans, namely precursor and neutral loss scans within a triple quadrupole mass spectrometer, Taguchi et al. (2005) showed how such an analysis is effective for: i) detecting and measuring individual molecular species without overlapping isobaric molecular species and ii) helping to improve the sensitivity of low abundant species (Taguchi et al., 2005). Thus, liquid chromatography coupled to tandem mass spectrometry using multiple reaction monitoring (MRM) is a powerful technique to accurately identify and quantify lipid classes.

In this study, we elaborate a novel strategy to determine the FA composition of lipid classes including phospholipids (PC, PE, PG, PI, PS and their lysophospholipid counterparts), galactolipids (MGDG, DGDG), non-polar lipids (MG, DG, TG) and the respective molecular species within each class (**Figure 1**). Two distinct LC-MS/MS methods using MRM scan survey were developed to analyze polar and non-polar lipid classes. Polar lipid classes were resolved by HILIC on an amine column while non-polar lipids were separated by FA composition on a solid core-shell C30 RP column. Following LC-MS/MS method development, five extraction procedures were tested on pennycress mature seeds and leaves for optimal recovery of the various lipid classes. Additionally, data analysis for TGs was strengthened by overlaying extracted ion chromatograms (XIC) of MRM transitions that had identical precursor ions but different product ions, and consequently revealing the FA composition for each TG parent molecular ion. On the other hand, polar lipids were quantified by one of two of the possible FA moieties. Thus, the quantification of multiple molecular species from multiple lipid classes could be determined through a stepwise and sequential analytical approach. Our lipidomics workflow was tested and validated on developing, mature, and germinated pennycress seeds/seedlings by monitoring all lipid classes mentioned in this paper and quantifying the ones that were above the limit of quantification. The results showed an accumulation of erucic acid in TG later in seed development and a potentially underappreciated role for PC in seed oil synthesis.

## 2 Materials and methods

### 2.1 Chemicals

The solvents, and chemicals used in this study: Acetonitrile (LC-MS grade), chloroform (HPLC grade), dichloromethane (LC-MS grade), ethanol (Molecular biology grade), ethyl acetate (HPLC grade), hexanes (LC-MS grade), isopropanol (LC-MS grade), methanol (LC-MS grade), acetic acid (LC-MS grade), ammonium acetate (LC-MS grade), ammonium formate (LC-MS grade), and formic acid (LC-MS grade) were purchased from Thermo Fisher Scientific (Waltham, MA, United States). Anhydrous Methyl-tert-butyl-ether (MTBE), butanol (Molecular biology grade), butylated-hydroxytoluene (BHT), and potassium chloride (KCl) (Molecular biology grade) were ordered from MilliporeSigma (Burlington, MA, United States). Ultrapure water (18.2 MΩ) was generated by a Milli-Q system from MilliporeSigma (Burlington, MA, United States).

UltimateSPLASH ONE internal standard mix, hydrogenated monogalactosyldiacylglycerol (MGDG), and hydrogenated digalactosyldiacylglycerol (DGDG) were obtained from Avanti Polar Lipids (Alabaster, AL, United States). Non-deuterated Monoacylglycerol (MG), diacylglycerol (DG), and triacylglycerol (TG) mentioned in this paper were ordered from Nu-Check Prep, Inc. (Elysian, MN, United States).

### 2.2 Plant materials and growth conditions

Pennycress seeds (Ames 32872 accession) were obtained from the United States Department of Agriculture-Germplasm Resources Information Network. Pennycress seeds and plants were respectively germinated and grown in a growth chamber as previously described by Tsogtbaatar et al. (2015); (2020).

Leaves from one week old (young) and three week old (mature) pennycress plants were collected in four biological replicates (four leaves per replicate for young plants and two leaves per replicate for mature plants), flash-frozen in liquid nitrogen and stored in a -80°C freezer until lyophilization.

Siliques were collected 14, 17, 20, and 23 days after pollination (DAP) and put in 50 mL conical tubes maintained on ice. Then, seeds were removed from the siliques, transferred to 2 mL screw capped tubes, flash-frozen in liquid nitrogen, and kept at -80°C until lyophilization. It is important to note that each replicate was comprised of 25, 20, 15, and 8 seeds (n = 4) were utilized for 14, 17, 20, and 23 DAP developing stages. Ten mature seeds per replicate (n = 4) from 10 week old pennycress plants were harvested and lyophilized for three days.

Seeds were germinated following the protocol published by Tsogtbaatar et al. (2015). Briefly, 50 pennycress seeds per Petri dish plate (n =4) were incubated for 5-7 days until the root length was 2-3 mm. Then, 10 seedlings were combined in a 2 mL screw capped tube, flash-frozen in liquid nitrogen, and stored in a -80°C freezer until lyophilization.

### 2.3 Preparation of non-polar and polar lipid standard stock and working solutions

Non-polar standard stock (10 mM) and working solutions (100 µM) were prepared in chloroform/methanol (50:50, v/v). The same stock and working concentrations were made for the polar lipid standards except they were resuspended in chloroform/methanol/water (89:10:1, v/v/v). The UltimateSPLASH ONE internal standard mix was used as is when added to the biological samples. All of the lipid standards were stored at -20°C.

### 2.4 Ultra high pressure liquid chromatography tandem mass spectrometry analysis

Both “non-polar” and “polar” UHPLC-MS/MS methods were performed on an Agilent 1290 Infinity II UHPLC (Santa Clara, CA, USA) coupled to a hybrid ABSciex QTRAP 6500+ ion trap/triple quadrupole mass spectrometer (Framingham, MA, USA). Non-polar and polar lipids were ionized using electrospray ionization (ESI), and detected and quantified using MRM scan survey.

#### 2.4.1 Lipid standard optimization for MRM scan survey

Commercially available lipid standards (**Table 1**) were diluted for optimizing the MS conditions as follows: i) non-polar lipids were prepared in acetonitrile/isopropanol/methanol/water (3:3:3:1; v/v/v/v) containing 2 mM ammonium formate; whereas ii) polar lipids were diluted in 50% aqueous ethanol plus 2 mM ammonium acetate. Final concentration before direct infusion to the mass spectrometer varied from 0.01 µM (TGs, DGs), 0.1µM for polar lipids (except MGDG and DGDG that were infused at 1 µM), to 1 µM for MGs. Optimization of the different lipids was performed using a 1 mL Hamilton syringe with a flow rate of 10 µL/min. The declustering (DP), entrance (EP), cell exit potentials (CXP), and collison energy (CE) were obtained through an automatic acquisition using Analyst 1.7 from ABSciex (see **Table 1**). The top five product ions were automatically determined for each standard precursor ion. For the final MRM method, the precursor/product ion with the highest intensity was selected for each standard. When standards were not available (for the polar and non-polar lipids), the MRM transition was extrapolated from the fragmentation of the available standards.

**Table 1:** Lipid-dependent MS parameters for MRM scan survey.

Lipid	Acyl chain combination	Precursor ion formula	Product ion formula	Precursor/Product transition (m/z)	DP* (V)	EP^¶^ (V)	CE^#^ (V)	CXP^§^ (V)
MG-17:0	17:0/0:0/0:0	C_20_H_44_ ${\text{NO}}_{4}^{+}$	C_17_H_33_O^+^	362.3/253.3	60	10	21	14
MG-17:1	17:1/0:0/0:0	C_20_H_42_ ${\text{NO}}_{4}^{+}$	C_17_H_31_O^+^	360.2/251.2	60	10	15	14
MG-19:0	19:0/0:0/0:0	C_22_H_48_ ${\text{NO}}_{4}^{+}$	C_19_H_37_O^+^	390.3/355.3	60	10	19	18
MG-19:1	19:1/0:0/0:0	C_22_H_46_ ${\text{NO}}_{4}^{+}$	C_19_H_35_O^+^	388.3/279.1	60	10	15	14
MG-19:2	19:2/0:0/0:0	C_22_H_44_ ${\text{NO}}_{4}^{+}$	C_19_H_33_O^+^	386.3/277.1	60	10	15	14
MG-21:0	21:0/0:0/0:0	C_24_H_52_ ${\text{NO}}_{4}^{+}$	C_21_H_41_O^+^	418.3/383.3	60	10	19	20
MG-22:1	22:1/0:0/0:0	C_25_H_52_ ${\text{NO}}_{4}^{+}$	C_22_H_41_O^+^	430.3/321.3	60	10	21	18
MG-24:1	24:1/0:0/0:0	C_27_H_56_ ${\text{NO}}_{4}^{+}$	C_24_H_45_O^+^	458.3/349.3	60	10	21	18
DG-31:1[D5]	17:0/14:1/0:0	C_34_H_63_D_5_ ${\text{NO}}_{5}^{+}$	C_20_H_34_D_5_ ${\text{O}}_{3}^{+}$	575.5/332.2	50	10	21	18
DG-33:1[D5]	17:0/16:1/0:0	C_36_H_67_D_5_ ${\text{NO}}_{5}^{+}$	C_19_H_30_D_5_ ${\text{O}}_{3}^{+}$	603.4/316.4	50	10	21	18
DG-35:1[D5]	17:0/18:1/0:0	C_38_H_71_D_5_ ${\text{NO}}_{5}^{+}$	C_21_H_34_D_5_ ${\text{O}}_{3}^{+}$	631.6/344.2	50	10	27	18
DG-37:3[D5]	17:0/20:3/0:0	C_40_H_71_D_5_ ${\text{NO}}_{5}^{+}$	C_23_H_34_D_5_ ${\text{O}}_{3}^{+}$	655.4/368.4	50	10	27	18
DG-39:4[D5]	17:0/22:4/0:0	C_42_H_73_D_5_ ${\text{NO}}_{5}^{+}$	C_20_H_34_D_5_ ${\text{O}}_{3}^{+}$	681.6/332.2	50	10	27	18
DG-44:2	22:1/22:1/0:0	C_47_H_92_ ${\text{NO}}_{5}^{+}$	C_25_H_47_ ${\text{O}}_{3}^{+}$	750.7/395.4	50	10	26	15
DG-48:2	24:1/24:1/0:0	C_51_H_100_ ${\text{NO}}_{5}^{+}$	C_27_H_51_ ${\text{O}}_{3}^{+}$	806.7/423.4	50	10	31	24
TG-41:0[D5]	14:0/13:0/14:0	C_44_H_83_D_5_ ${\text{NO}}_{6}^{+}$	C_30_H_52_D_5_ ${\text{O}}_{4}^{+}$	731.4/486.4	80	10	33	24
TG-43:1[D5]	14:0/15:1/14:0	C_46_H_85_D_5_ ${\text{NO}}_{6}^{+}$	C_32_H_54_D_5_ ${\text{O}}_{4}^{+}$	757.4/512.4	80	10	33	24
TG-45:1[D5]	14:0/17:1/14:0	C_48_H_89_D_5_ ${\text{NO}}_{6}^{+}$	C_34_H_58_D_5_ ${\text{O}}_{4}^{+}$	785.5/540.6	80	10	33	24
TG-47:1[D5]	16:0/15:1/16:0	C_50_H_93_D_5_ ${\text{NO}}_{6}^{+}$	C_34_H_58_D_5_ ${\text{O}}_{4}^{+}$	813.4/540.4	80	10	39	18
TG-49:1[D5]	16:0/17:1/16:0	C_52_H_97_D_5_ ${\text{NO}}_{6}^{+}$	C_36_H_62_D_5_ ${\text{O}}_{4}^{+}$	841.4/568.4	80	10	33	24
TG-51:2[D5]	16:0/19:2/16:0	C_54_H_99_D_5_ ${\text{NO}}_{6}^{+}$	C_38_H_64_D_5_ ${\text{O}}_{4}^{+}$	867.4/594.4	80	10	37	16
TG-53:3[D5]	18:1/17:1/18:1	C_56_H_101_D_5_ ${\text{NO}}_{6}^{+}$	C_38_H_64_D_5_	893.8/594.6	80	10	37	16
TG-54:9	18:3/18:3/18:3	C_57_H_96_ ${\text{NO}}_{6}^{+}$	C_39_H_63_ ${\text{O}}_{4}^{+}$	890.8/595.6	80	10	37	28
TG-55:4[D5]	18:1/19:2/18:1	C_58_H_103_D_5_ ${\text{O}}_{6}^{+}$	C_40_H_66_D_5_ ${\text{O}}_{4}^{+}$	919.6/620.6	80	10	39	18
TG-57:4[D5]	18:1/21:2/18:1	C_60_H_107_D_5_ ${\text{O}}_{6}^{+}$	C_42_H_70_D_5_ ${\text{O}}_{4}^{+}$	947.8/648.6	80	10	39	18
TG-66:3	22:1/22:1/22:1	C_69_H_132_ ${\text{NO}}_{6}^{+}$	C_47_H_87_ ${\text{O}}_{4}^{+}$	1071.1/715.8	80	10	43	32
TG-72:3	24:1/24:1/24:1	C_75_H_144_ ${\text{NO}}_{6}^{+}$	C_51_H_95_ ${\text{O}}_{4}^{+}$	1155.4/771.8	80	10	43	32
**MGDG-34:0**	**16:0/18:0**	**C_43_H_81_** ${\text{O}}_{10}^{-}$	**C_18_H_35_** ${\text{O}}_{2}^{-}$	**757.6/283.2**	**-80**	**-10**	**-30**	**-15**
**DGDG-36:0**	**18:0/18:0**	**C_51_H_95_** ${\text{O}}_{15}^{-}$	**C_18_H_35_** ${\text{O}}_{2}^{-}$	**947.7/283.2**	**-180**	**-10**	**-40**	**-15**
**LPC-15:0[D5]**	**15:0/0:0**	**C_25_H_46_D_5_NO_9_P^-^**	**C_15_H_29_** ${\text{O}}_{2}^{-}$	**545.4/241.2**	**-40**	**-10**	**-40**	**-13**
**LPC-17:0[D5]**	**17:0/0:0**	**C_27_H_50_D_5_NO_9_P^-^**	**C_17_H_33_** ${\text{O}}_{2}^{-}$	**573.4/269.2**	**-40**	**-10**	**-40**	**-13**
**LPC-19:0[D5]**	**19:0/0:0**	**C_29_H_54_D_5_NO_9_P^-^**	**C_19_H_37_** ${\text{O}}_{2}^{-}$	**601.4/297.3**	**-40**	**-10**	**-40**	**-13**
**LPC-17:1**	**17:1/0:0**	**C_27_H_53_NO_9_P^-^**	**C_17_H_31_** ${\text{O}}_{2}^{-}$	**566.4/267.2**	**-40**	**-10**	**-40**	**-13**
**LPE-15:0[D5]**	**15:0/0:0**	**C_20_H_36_D_5_NO_7_P^-^**	**C_15_H_29_** ${\text{O}}_{2}^{-}$	**443.4/241.2**	**-110**	**-10**	**-30**	**-13**
**LPE-17:0[D5]**	**17:0/0:0**	**C_22_H_40_D_5_NO_7_P^-^**	**C_17_H_33_** ${\text{O}}_{2}^{-}$	**471.4/269.1**	**-110**	**-10**	**-30**	**-13**
**LPE-19:0[D5]**	**19:0/0:0**	**C_24_H_44_D_5_NO_7_P^-^**	**C_19_H_37_** ${\text{O}}_{2}^{-}$	**499.4/297.3**	**-110**	**-10**	**-30**	**-13**
**LPE-18:1**	**18:1/0:0**	**C_23_H_45_NO_7_P^-^**	**C_18_H_33_** ${\text{O}}_{2}^{-}$	**478.3/281.3**	**-110**	**-10**	**-30**	**-13**
**LPG-15:0[D5]**	**15:0/0:0**	**C_21_H_37_D_5_O_9_P^-^**	**C_15_H_29_** ${\text{O}}_{2}^{-}$	**474.3/241.1**	**-100**	**-10**	**-34**	**-17**
**LPG-17:0[D5]**	**17:0/0:0**	**C_23_H_41_D_5_O_9_P^-^**	**C_17_H_33_** ${\text{O}}_{2}^{-}$	**502.3/269.2**	**-100**	**-10**	**-34**	**-17**
**LPG-19:0[D5]**	**19:0/0:0**	**C_25_H_45_D_5_O_9_P^-^**	**C_19_H_37_** ${\text{O}}_{2}^{-}$	**530.4/297.2**	**-100**	**-10**	**-34**	**-17**
**LPG-17:1**	**17:1/0:0**	**C_23_H_44_O_9_P^-^**	**C_17_H_31_** ${\text{O}}_{2}^{-}$	**495.2/267.2**	**-100**	**-10**	**-34**	**-17**
**LPI-15:0[D5]**	**15:0/0:0**	**C_24_H_41_D_5_O_12_P^-^**	**C_15_H_29_** ${\text{O}}_{2}^{-}$	**562.4/241.2**	**-110**	**-10**	**-44**	**-13**
**LPI-17:0[D5]**	**17:0/0:0**	**C_26_H_45_D_5_O_12_P^-^**	**C_17_H_33_** ${\text{O}}_{2}^{-}$	**590.2/269.3**	**-110**	**-10**	**-44**	**-13**
**LPI-19:0[D5]**	**19:0/0:0**	**C_28_H_49_D_5_O_12_P^-^**	**C_19_H_37_** ${\text{O}}_{2}^{-}$	**618.4/297.2**	**-110**	**-10**	**-44**	**-13**
**LPI-18:1**	**18:1/0:0**	**C_27_H_50_O_12_P^-^**	**C_18_H_33_** ${\text{O}}_{2}^{-}$	**597.3/281.3**	**-110**	**-10**	**-44**	**-13**
**LPS-15:0[D5]**	**15:0/0:0**	**C_21_H_36_D_5_NO_9_P^-^**	**C_3_HD_5_O_5_P^-^**	**487.4/158.0**	**-40**	**-10**	**-34**	**-9**
**LPS-17:0[D5]**	**17:0/0:0**	**C_23_H_40_D_5_NO_9_P^-^**	**C_3_HD_5_O_5_P^-^**	**515.4/158.0**	**-40**	**-10**	**-34**	**-9**
**LPS-19:0[D5]**	**19:0/0:0**	**C_25_H_44_D_5_NO_9_P^-^**	**C_3_HD_5_O_5_P^-^**	**543.3/158.0**	**-40**	**-10**	**-34**	**-9**
**LPS-17:1**	**17:1/0:0**	**C_23_H_43_NO_9_P^-^**	**C_17_H_31_** ${\text{O}}_{2}^{-}$	**508.4/267.2**	**-40**	**-10**	**-34**	**-9**
**PC-31:1[D5]**	**14:1/17:0**	**C_41_H_74_D_5_NO_10_P^-^**	**C_14_H_25_** ${\text{O}}_{2}^{-}$	**781.6/225.2**	**-65**	**-10**	**-52**	**-15**
**PC-33:1[D5]**	**16:1/17:0**	**C_43_H_78_D_5_NO_10_P^-^**	**C_16_H_29_** ${\text{O}}_{2}^{-}$	**809.6/253.2**	**-65**	**-10**	**-52**	**-15**
**PC-35:1[D5]**	**18:1/17:0**	**C_45_H_82_D_5_NO_10_P^-^**	**C_18_H_33_** ${\text{O}}_{2}^{-}$	**837.6/281.2**	**-65**	**-10**	**-52**	**-15**
**PC-37:3[D5]**	**20:3/17:0**	**C_47_H_82_D_5_NO_10_P^-^**	**C_20_H_33_** ${\text{O}}_{2}^{-}$	**861.6/305.2**	**-65**	**-10**	**-52**	**-15**
**PC-39:4[D5]**	**22:4/17:0**	**C_49_H_84_D_5_NO_10_P^-^**	**C_22_H_35_** ${\text{O}}_{2}^{-}$	**887.6/331.2**	**-65**	**-10**	**-52**	**-15**
**PE-31:1[D5]**	**14:1/17:0**	**C_36_H_64_D_5_NO_8_P^-^**	**C_14_H_25_** ${\text{O}}_{2}^{-}$	**679.4/225.2**	**-120**	**-10**	**-38**	**-13**
**PE-33:1[D5]**	**16:1/17:0**	**C_38_H_68_D_5_NO_8_P^-^**	**C_16_H_29_** ${\text{O}}_{2}^{-}$	**707.6/253.2**	**-120**	**-10**	**-38**	**-13**
**PE-35:1[D5]**	**18:1/17:0**	**C_40_H_72_D_5_NO_8_P^-^**	**C_18_H_33_** ${\text{O}}_{2}^{-}$	**735.6/281.4**	**-120**	**-10**	**-38**	**-13**
**PE-37:3[D5]**	**20:3/17:0**	**C_42_H_72_D_5_NO_8_P^-^**	**C_20_H_33_** ${\text{O}}_{2}^{-}$	**759.6/305.2**	**-120**	**-10**	**-38**	**-13**
**PE-39:4[D5]**	**22:4/17:0**	**C_44_H_74_D_5_NO_8_P^-^**	**C_22_H_35_** ${\text{O}}_{2}^{-}$	**785.6/331.2**	**-120**	**-10**	**-38**	**-13**
**PG-31:1[D5]**	**14:1/17:0**	**C_37_H_65_D_5_O_10_P^-^**	**C_14_H_25_** ${\text{O}}_{2}^{-}$	**710.6/225.2**	**-120**	**-10**	**-44**	**-13**
**PG-33:1[D5]**	**16:1/17:0**	**C_39_H_69_D_5_O_10_P^-^**	**C_16_H_29_** ${\text{O}}_{2}^{-}$	**738.4/253.2**	**-120**	**-10**	**-44**	**-13**
**PG-35:1[D5]**	**18:1/17:0**	**C_41_H_73_D_5_O_10_P^-^**	**C_18_H_33_** ${\text{O}}_{2}^{-}$	**766.5/281.3**	**-120**	**-10**	**-44**	**-13**
**PG-37:3[D5]**	**20:3/17:0**	**C_43_H_73_D_5_O_10_P^-^**	**C_20_H_33_** ${\text{O}}_{2}^{-}$	**790.4/305.2**	**-120**	**-10**	**-44**	**-13**
**PG-39:4[D5]**	**22:4/17:0**	**C_45_H_75_D_5_O_10_P^-^**	**C_22_H_35_** ${\text{O}}_{2}^{-}$	**816.4/331.2**	**-120**	**-10**	**-44**	**-13**
**PI-31:1[D5]**	**14:1/17:0**	**C_40_H_69_D_5_O_13_P^-^**	**C_17_H_33_** ${\text{O}}_{2}^{-}$	**798.6/269.2**	**-55**	**-10**	**-60**	**-15**
**PI-33:1[D5]**	**16:1/17:0**	**C_42_H_73_D_5_O_13_P^-^**	**C_16_H_29_** ${\text{O}}_{2}^{-}$	**826.6/253.2**	**-55**	**-10**	**-60**	**-15**
**PI-35:1[D5]**	**18:1/17:0**	**C_44_H_77_D_5_O_13_P^-^**	**C_18_H_33_** ${\text{O}}_{2}^{-}$	**854.6/281.2**	**-55**	**-10**	**-60**	**-15**
**PI-37:3[D5]**	**20:3/17:0**	**C_46_H_77_D_5_O_13_P^-^**	**C_20_H_33_** ${\text{O}}_{2}^{-}$	**878.6/305.2**	**-55**	**-10**	**-60**	**-15**
**PI-39:4[D5]**	**22:4/17:0**	**C_48_H_79_D_5_O_13_P^-^**	**C_22_H_35_** ${\text{O}}_{2}^{-}$	**904.6/331.2**	**-55**	**-10**	**-60**	**-15**
**PS-31:1[D5]**	**14:1/17:0**	**C_37_H_64_D_5_NO_10_P^-^**	**C_17_H_33_** ${\text{O}}_{2}^{-}$	**723.6/269.3**	**-60**	**-10**	**-50**	**-13**
**PS-33:1[D5]**	**16:1/17:0**	**C_39_H_68_D_5_NO_10_P^-^**	**C_16_H_29_** ${\text{O}}_{2}^{-}$	**751.6/253.2**	**-60**	**-10**	**-50**	**-13**
**PS-35:1[D5]**	**18:1/17:0**	**C_41_H_72_D_5_NO_10_P^-^**	**C_17_H_33_** ${\text{O}}_{2}^{-}$	**779.6/269.3**	**-60**	**-10**	**-50**	**-13**
**PS-37:3[D5]**	**20:3/17:0**	**C_43_H_72_D_5_NO_10_P^-^**	**C_20_H_33_** ${\text{O}}_{2}^{-}$	**803.6/305.2**	**-60**	**-10**	**-50**	**-13**
**PS-39:4[D5]**	**22:4/17:0**	**C_45_H_74_D_5_NO_10_P^-^**	**C_22_H_35_** ${\text{O}}_{2}^{-}$	**829.6/331.2**	**-60**	**-10**	**-50**	**-13**

*DP: Declustering Potential, ^¶^EP: Entrance Potential, ^#^CE: Collision Energy, ^§^CXP: Collision cell Exit Potential, are depicted for each metabolite. Polar lipids are highlighted in bold.

Once direct infusion was accomplished to establish MS and MRM conditions, we proceeded to chromatographic trials in order to find the best chromatographic separation for the polar and non-polar lipids. Once those were defined, source optimization was performed automatically for the curtain (CUR; 25 to 40 psi; 5 psi increment), nebulizing (GS1; 40 to 70 psi with 5 psi increment), heating (GS2; 40 to 70 psi with 5 psi increment) gases, the temperature (TEM, 350 to 700°C; 50°C increment), the collision associated dissociation (CAD; low, medium and high) as well as the ion spray voltage (IS; 3000 to 5500 V or -3000 to -4500 V; 500 V increment) to maximize sensitivity for both methods used in this paper (see source parameters under “2.4.2 Polar lipid method” and “2.4.3 Non-polar lipid method” below).

#### 2.4.2 Polar lipid method

Polar lipids were separated by the polarity of their headgroups on a Phenomenex (Torrance, CA, United States) Luna NH_2_ column (150 x 4.6 mm, 3 μm, 100 Å). The mobile phases consisted of: A) acetonitrile:water:hexane (92:6:2, v/v/v) with 2 mM ammonium acetate; B) acetonitrile:water (50:50, v/v) with 2 mM ammonium acetate at pH 9.35 (ammonium hydroxide was used to buffer solvent B). The total run time was 19 minutes with the following solvent gradient: 0.0 min, 0% B; 6.5 min, 40% B; 7.0 min, 55% B; 9.0 min, 55% B; 9.5 min, 70% B; 12.0 min, 70% B; 12.1 min, 85% B; 14.5 min, 85% B; 14.6 min, 100% B; 17.0 min, 100% B; 17.1 min, 0% B; 19.0 min, 0% B. The flow rate was 1 mL/min, injection volumes were 5 μL, the oven temperature was set to 25°C as well as the autosampler.

After chromatographic resolution, polar lipids were ionized by ESI in negative ion mode. CUR, GS1, GS2 gases were set to 30, 40, and 40 psi, respectively. Optimal parameters for IS, CAD, and TEM were 4500 V, high, and 650°C, respectively. Mass spectra for polar lipids were acquired using scheduled MRM with a cycling time of one second and MRM windows of 180 seconds. Analyst 1.7 and Multiquant 3.0.3 from ABSciex were utilized to collect and quantify the data, respectively.

#### 2.4.3 Non-polar lipid method

Non-polar lipids were resolved by the properties of their fatty acids, namely the degree of unsaturation and carbon length, on a ThermoFisher Scientific Accucore C30 column (150 x 2.1 mm, 2.6 μm, 150 Å). The mobile phases were made of: A) acetonitrile:water (60:40, v/v) with 10 mM ammonium formate and 0.1% formic acid; B) isopropanol:acetonitrile:water (90:10:1, v/v/v) with 10 mM ammonium formate and 0.1% formic acid. The total run time was 35 minutes with the following solvent gradient: 0 min, 50% B; 3 min, 70% B; 23 min, 90% B; 24 min, 95% B; 28 min, 99% B; 32 min, 99% B; 33 min, 50% B; 35 min, 50% B. It is important to note that the flow rate was not constant: 0-23 min, 0.350 mL/min; 23-24 min, 0.575 mL/min; 24-32 min, 0.575 mL/min, 32-33 min, 0.350 mL/min; 33-35 min, 0.350 mL/min. Injection volumes were 5 μL, the oven temperature was set to 40°C, and the samples were kept in the autosampler at 25°C.

Non-polar lipids eluting from the column were ionized by ESI in positive ion mode. The following optimized source parameters were selected: positive polarity, CUR of 25 psi, “medium” for CAD, 350°C for TEM, 65 psi for GS1 and 50 psi for GS2, and 5500 V for IS. Mass spectra for non-polar lipids were acquired using MRM with a dwell time of 1.5 milliseconds for each precursor/product ion transition. Analyst 1.7 and Multiquant 3.0.3 from ABSciex were used to collect and quantify the data, respectively.

**Figure 2:**
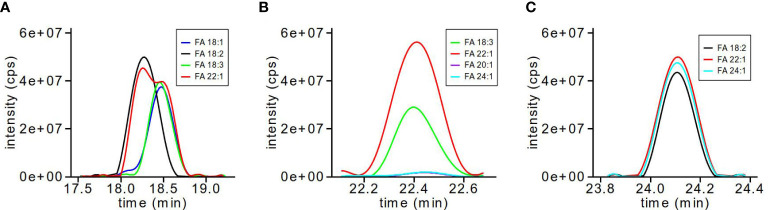
TG MRM transitions for FA composition analysis. The XIC for individual TG-FA MRM transitions are overlaid to determine which TG-FA MRMs are used in quantification and data analysis. TG-58:5 **(A)** shows two molecular species as 18:2_18:2_22:1 and 18:1_18:3_22:1; TG-62:5 **(B)** shows two molecular species as 18:3_22:1_22:1 and 18:3_20:1_24:1; TG-64:4 **(C)** shows one molecular species as 18:2_22:1_24:1.

As TG molecular species are capable of a number of possible FA combinations with the same molecular mass, we calculated MRMs for each of the likely FA fragments for each TG molecular ion. In order to determine the FA composition for each of the chromatographically separated TG molecular species, the XIC of each FA fragmentation was overlaid to determine which three FAs contributed to the parent TG molecular ion. **Figure 2** shows three examples of the types of overlaid possibilities for FA combinations. For example, TG-58:5 (**Figure 2A**) shows two molecular species, 18:2_18:2_22:1 and 18:1_18:3_22:1, where the FA 22:1 XIC (red) is shared between two sets of peaks; TG-62:5 (**Figure 2B**) also shows two molecular species, 18:3_22:1_22:1 and a less certain 18:3_20:1_24:1, where both sets of peaks are overlapping, though the XIC intensities differ between the molecular species, with the FA 22:1 double than the FA 18:3 transition; and finally TG-64:4 (**Figure 2C**) shows the simplest possibility of three FA XIC overlaid with nearly identical intensities for 18:2_22:1_24:1.

### 2.5 Total lipid extraction methods and sample preparation

After lyophilization of pennycress tissues (mature seeds and mature leaves) at -80°C for three days, dried samples were weighed prior to lipid extraction. Leaves were transferred to 15 mL containers and pulverized at 1,750 rpm for 3 minutes using a Geno grinder 2010 from Spex Sample Prep (Metuchen, NJ, United States). 10 ± 0.5 mg of leaf powder was weighed and transferred to 2 mL screw capped tubes. The same amount of material was utilized for pennycress mature seeds.

Following the different extractions, all samples were dried under a stream of nitrogen at 40°C before resuspending in 1 mL of dichloromethane:methanol (1:1, v/v). Resuspended samples were further diluted in solvents appropriate to either the polar or non-polar lipid methods. For the quantification of non-polar lipids, 10 μL of extract was diluted into 490 μL of acetonitrile:isopropanol:methanol:water (3:3:3:1, v/v/v/v) containing 10 mM ammonium formate and 0.1% formic acid whereas 10 μL of extract was added to 490 μL of 100% ethanol containing 2 mM ammonium acetate for the analysis of polar lipids.

Five different lipid extraction methods were tested in this study and relatively compared to one another using the LC-MS peak area normalized to the tissue mass used in extraction, as described below:

#### 2.5.1 Method 1 (hexanes/isopropanol)

A hexanes/isopropanol method was adapted from Hara and Radin (1978). One mL of hexanes:isopropanol (3:2, v/v) was added to 2 mL screw cap tubes containing 10 ± 0.5 mg of mature seed or mature leaf samples and a 5 mm tungsten bead. Samples were disrupted for 5 minutes at 30 Hz at room temperature using a mixer mill MM400 from Retsch (Haan, Germany). Homogenized samples were centrifuged at 17,000 *g* for 5 minutes at room temperature, and the supernatants were transferred to 13 x 100 mm screw cap glass tubes using a Pasteur pipet. The homogenates were re-extracted two more times with an additional 1 mL of hexanes:isopropanol (3:2, v/v) each time, vortexed, and centrifuged. Supernatants were combined, dried and resuspended as indicated above.

#### 2.5.2 Method 2 (isopropanol/chloroform – #1)

An isopropanol/chloroform/methanol-based method was adapted from Shiva et al. (2018). Tissues were homogenized as described in *Method 1* using 1 mL of isopropanol heated to 70°C containing 0.01% butylated-hydroxytoluene (BHT). Homogenized extracts were transferred to 16 x 100 mm screw cap glass tubes. An additional 1 mL of 70°C isopropanol containing 0.01% BHT was used to rinse out the 2 mL homogenization screw cap tubes and combined with the previous extract. The samples were then extracted as described by Shiva et al. (2018). Samples were dried and resuspended as discussed above.

#### 2.5.3 Method 3 (isopropanol/chloroform – #2)

Another isopropanol/chloroform-based method was used, adapted and modified from Chapman and Moore (1993). Similar to *Method 2*, samples were homogenized in 1 mL of 70°C isopropanol containing 0.01% BHT, and the emptied homogenized tubes rinsed with 1 mL of 70°C isopropanol containing 0.01% BHT. One mL of chloroform and 0.45 mL of ultrapure water was added to the homogenate before incubating overnight at 4°C. Samples equilibrated to room temperature were vortexed and then centrifuged at 2,000 *g* for 5 minutes at 25°C. The supernatants were transferred to 16 x 100 mm screw cap glass tubes using a Pasteur pipet. To the remaining homogenate 2 mL of isopropanol, 1 mL of chloroform, and 0.45 mL of ultrapure water was added to re-extract the residual tissue, followed by vortexing and centrifugation at 2,000 *g* for 5 minutes at 25°C. The resulting supernatants were combined with the previous. One mL of chloroform and 2 mL of 1 M KCl were added to the collected supernatants, vortexed, and centrifuged at 2,000 *g* for 5 minutes at 25°C to induce phase separation. The aqueous upper phase was aspirated off, and the lower phase washed again with an additional 2 mL of 1 M KCl. Following a second aspiration, the lower phase was dried and prepared for LC-MS/MS as described above.

#### 2.5.4 Method 4 (butanol/methanol)

A butanol/methanol method was adapted from Löfgren et al. (2016). Homogenization of samples took place in 500 μL of butanol:methanol (3:1, v/v). Then, 500 μL of hexanes:ethyl acetate (3:1, v/v) and 500 μL of 1% acetic acid were added. Samples were vortexed and centrifuged at 17,000 *g* for 5 minutes at room temperature. The upper butanolic phases (600 μL) were transferred to 13 x 100 mm glass tubes using a Pasteur pipet. 500 μL of hexanes/ethyl acetate (3:1, v/v) was added to the remaining lower phases, vortexed, and centrifuged at 17,000 *g* for 5 minutes at room temperature. The resulting upper phases were combined. Extracts were dried, resuspended, and diluted as specified above.

#### 2.5.5 Method 5 (methyl tert butyl ether)

A methyl tert-butyl ether (MTBE) method was adapted from Matyash et al. (2008). Mature seeds and mature leaves were homogenized in 1 mL of methanol. Homogenates were transferred to 13 x 100 mm screw cap glass tubes using a Pasteur pipet. One mL of MTBE was used to rinse the 2 mL screw cap tubes and combined to the previous extract. The rinsing step was repeated once more. An additional 1.3 mL of MTBE was added to the combined homogenates, vortexed, and then incubated at room temperature for 1 hour under constant shaking at 150 rpm. To induce phase separation, 0.8 mL of ultrapure water was added to the combined homogenates, vortexed, and incubated for 3 minutes at room temperature. Samples were centrifuged at 1,000 *g* for 5 minutes at room temperature using a benchtop centrifuge. Upper phases were transferred to new 13 x 100 mm screw cap glass tubes. To the remaining lower phases, 1.3 mL of MTBE:methanol:water (10:3:2.5, v/v/v) was added, vortexed, and then centrifuged at 1,000 *g* for 5 minutes at room temperature. The upper phases were collected, combined with the first, and then dried under nitrogen gas before preparing for LC-MS/MS as indicated above.

### 2.6 Method validation

#### 2.6.1 Linearity, limit of detection, and limit of quantification

Standard curves using neat standards were generated using serial dilution (9 points) for the different lipids used in this study. The non-polar lipids were diluted in acetonitrile:isopropanol:methanol:water (3:3:3:1, v/v/v/v) containing 10 mM ammonium formate and 0.1% formic acid. Serial dilutions of polar lipids were prepared in 100% ethanol containing 2 mM ammonium acetate. The coefficient of correlation, the linearity as well as the limit of detection (LOD) and the limit of quantification (LOQ) for each lipid were calculated from the standard curves. LOD and LOQ were defined as 3 and 10 times the signal to noise, respectively.

#### 2.6.2 Recovery efficiency & matrix effect

RE and ME for the non-polar and polar lipids mentioned in **Table 2** were assessed as described by Cocuron et al. (2019).

**Table 2:** Matrix effect (%) and recovery efficiency (%) for non-polar and polar lipids.

Lipid	ME (%)* (n = 5)	RE (%)** (n = 5)
DG-31:1[D5]	1.6	104.3
DG-35:1[D5]	5.9	79.0
DG-39:4[D5]	8.5	88.8
TG-45:1[D5]	11.1	80.8
TG-53:3[D5]	20.6	87.5
TG-57:4[D5]	7.0	83.9
**MGDG-34:0**	***-32.0***	**96.3**
**DGDG-36:0**	**-18.5**	**85.7**
**LPC-15:0[D5]**	**-18.0**	**75.9**
**LPC-19:0[D5]**	**-20.6**	**87.3**
**LPE-15:0[D5]**	**-14.8**	**85.0**
**LPE-19:0[D5]**	***-26.0***	**81.6**
**LPG-15:0[D5]**	**-23.4**	***53.0***
**LPG-19:0[D5]**	**-19.1**	***65.6***
**LPI-15:0[D5]**	**-18.0**	***35.9***
**LPI-19:0[D5]**	**-11.7**	***51.3***
**LPS-15:0[D5]**	**3.4**	***36.5***
**LPS-19:0[D5]**	**1.3**	***58.0***
**PC-31:1[D5]**	***-28.7***	**89.1**
**PC-35:1[D5]**	***-38.9***	**91.4**
**PE-31:1[D5]**	***-37.3***	**114.5**
**PE-35:1[D5]**	***-32.1***	**93.0**
**PG-31:1[D5]**	**-7.9**	**95.7**
**PG-35:1[D5]**	**-16.9**	**89.0**
**PI-31:1[D5]**	**-19.4**	**77.1**
**PI-35:1[D5]**	**-23.2**	**88.8**
**PS-31:1[D5]**	**-6.9**	**86.2**
**PS-35:1[D5]**	**-7.6**	**90.1**

Lipids showing a ME ± 25% and having a RE< 75% are depicted in italic. Polar lipids are highlighted in bold.

*Matrix effect.

^**^Recovery efficiency.

Briefly, RE was determined using five pennycress seed samples with internal standards spiked before extraction compared to five pennycress seed lipid extracts spiked with internal standards following extraction. The recovery for the different standards for each of the lipid classes analyzed were calculated as the ratio of signal intensities of those spiked before over those spiked after using the following equation (1):


(1) $$\text{RE}=\frac{IS_{before}}{IS_{after}}\times 100\%$$

Where *RE* is the recovery efficiency, *IS_before_* is the internal standard signal intensity of samples spiked before extraction, *IS_after_* is the internal standard signal intensity of spiked extracts after extraction.

ME was calculated using five pennycress seed lipid extracts with internal standards spiked after extraction compared to five samples containing only the internal standards without lipid extract. The ratio of the signal intensities of internal standards spiked into lipid extracts over the signal intensities of the internal standards alone were calculated using equation (2):


(2) $$ME=\left(\frac{IS_{after}}{IS_{alone}}\times 100\%\right)-100\%$$

Where *ME* is the matrix effect, *IS_after_* is the internal standard signal intensity of spiked extracts after extraction, and *IS_alone_* is the signal intensity of standards analyzed without lipid extract. Equation (2) provides the percentage change from an expected 100% if there were no matrix effect. A negative value indicates a matrix effect that decreases the expected signal intensity, and a positive value vice versa.

#### 2.6.3 GC-MS analysis of fatty acid methyl esters

Oil from pennycress seeds was analyzed by GC-MS after derivatization to FAMEs as previously described by Johnston et al. (2022).

### 2.7 Targeted lipidomics study on different pennycress organs

This work was performed on pennycress developing seeds, mature seeds, seedlings, as well as young and mature leaves. Details about the number of seeds and leaves are reported in section “2.2 Plant materials and growth conditions”.

Briefly, 10 ± 0.5 mg of dried tissues were extracted following the MTBE method (Method 5) described in the “2.5 Total lipids extraction methods and sample preparation” section. A mixture of internal standards consisting of 10 μL of UltimateSPLASH ONE internal standard, 2.5 μL of 10 mM hydrogenated monogalactosyldiacylglycerol (MGDG), and 2.5 μL of 10 mM hydrogenated digalactosyldiacylglycerol (DGDG) was added just before homogenization of the sample. These internal standards were used for the normalization of the samples and the quantification of total lipids. Galactolipid standards (MGDG and DGDG) were prepared in 100% chloroform for a stock concentration of 10 mM. Following extraction, non-polar and polar lipids were analyzed using the “non-polar lipid” and “polar lipid” LC-MS/MS methods described in the section “2.4 Ultra High Pressure Liquid Chromatography tandem Mass Spectrometry (UHPLC-MS/MS) analysis”. Peak integration, quantification of lipids and statistical analysis were performed as described in the next section.

### 2.8 Data collection and analysis

Analyst software (v1.7) was used to both operate the LC-MS/MS and collect data. Multiquant 3.0.3 software was used to integrate peaks and to export processed data.

The values for the integrated peaks for each lipid molecular species and each class were entered into a tab-delimited text file. This file was used with a custom R script to normalize the molecular species and classes by a single internal standard from the UltimateSPLASH mix corresponding to the lipid class and the initial tissue mass to give a relative nmol/mg amount (R v3.6.3 and RStudio v1.1.423). Integrated peak values were multiplied by the nmol amount of the lipid internal standard used for each class and divided by the integrated peak area of the internal standard (see **Supplementary Table 1** for internal standards used for each lipid class). The R script was also written to output plotted bar charts of the total average sum of each lipid class and the average molecular species distribution. Lastly, data tables formatted for use with Metaboanalyst v5.0 were given and used for statistical analysis (Pang et al., 2021).

A preliminary step for TG quantification that identified the FA composition was required before data processing. The XICs of each of the MRMs for the FA loss of TGs were exported as text files. Each XIC for the MRM FA loss corresponding to individual TG molecular species was plotted and overlaid against each other to determine which FA combination gave rise to the parent TG molecular species. One of the three TG-FA MRMs were selected for later quantification for each of the possible FA combinations of each TG molecular species. Those TG-FA MRMs that were shared among a number of possible FA combinations, or were multiples within the same TG molecular species, were divided by the appropriate number to reduce the multiple counts (e.g. TG-54:3 (18:1_18:1_18:1) divided by 3 for the multiple 18:1 FAs). The selected TG-FA MRMs were added to the R script that quantified the integrated peaks exported from Multiquant.

## 3 Results

### 3.1 Determination of optimal mass spectrometry parameters for non-polar and polar lipids

In this study, we focused on the major lipid classes found in most seed, seedling and leaf tissues: monoacylglycerols (MG), diacylglycerols (DG), and triacylglycerols (TG) representing the non-polar lipids, and phosphatidylcholines (PC), phosphatidylethanolamines (PE), phosphatidylinositols (PI), phosphatidylglycerols (PG), phosphatidylserines (PS), and their lyso- counterparts, monogalactosyldiacylglycerols (MGDG), and digalactosyldiacylglycerols (DGDG) representing the polar lipids (**Figure 1**).

In order to obtain a robust targeted lipidomics LC-MS/MS method relying on MRM scan survey, representative lipids from each class were directly infused into the mass spectrometer for compound optimization, as described in Materials and Methods. This determined the most abundant product ions after fragmenting the precursor ion. Once each lipid was processed, the Analyst software classified pairs of precursor/product ions according to their abundance with their optimal ionization parameters (**Table 1**).

The results of the direct infusion of MGs, DGs and TGs showed that the ammoniated precursor ions ([M + NH_4_]^+^) were predominant over the protonated form ([M + H]^+^), and that positive ionization was the only polarity under which these non-polar lipids were detectable. Product ions of DGs and TGs were characterized by a loss of NH_3_ and an acyl side-chain to produce a monoacyl or diacyl product ions, respectively. Product ions for MGs were identified as a loss of NH_3_ and glycerol (**Table 1** and **Supplementary Table 2**).

Polar lipids were detectable under negative and positive ionizations. Negative ionization was chosen in this work as it is able to determine the FA composition of the different phospholipids and galactolipids (Kerwin et al., 1994; Welti et al., 2003). For most of the phospholipids, precursor ions were detected as deprotonated forms ([M-H]^-^) with the exception of PC and LPC whose acetate adducts ([M + CH_3_COO]^-^) were more abundant. The fragment ion masses corresponded to the deprotonated acyl side chain ([M-H]^-^) (**Table 1** and **Supplementary Table 3**).

Using the optimized MRM transitions for the non-polar and polar lipid standards and an initial LC-MS/MS method, we proceeded to develop reliable liquid chromatography methods to fully resolve lipid classes and molecular species. Source optimization was then conducted for the non-polar and polar lipids with the same pool of lipids mentioned in **Table 1**.

### 3.2 Development of LC-MS/MS methods for the detection and quantification of non-polar and polar lipids

Initially HILIC chromatography was used to separate both polar and non-polar lipids using an amine column. However, non-polar lipids eluted within the void volume (between 1.5-2.5 minutes), resulting in imprecise and inaccurate quantification, and the inability to determine FA compositions of TG molecular species. Therefore, we opted to develop two chromatographic methods: i) a HILIC method to separate polar lipids on a NH_2_ amine column, and ii) a RP method to separate non-polar lipids on a C30 core-shell column. Using two methods rather than one had the advantage of preventing peak overlap that may confound the quantification process and prohibit the ability to identify FA compositions. Additionally, the use of the C30 column for TG analysis allows determining the FA composition of isobaric TG molecular species. We also found in our initial attempts that the lipid classes PA and lyso-PA (LPA) did not resolve well, had considerable amounts of carryover from injection to injection, and had variable retention times. Both PA and LPA are known to be difficult lipid classes to analyze *via* LC-MS possibly due to binding to metal surfaces of the LC system (Ogiso et al., 2008; Cífková et al., 2016). There are LC systems that have surface coatings that minimize such binding interactions and have been shown to improve detection of PA and LPA (Isaac et al., 2022). However, the Agilent 1290 Infinity II UHPLC used in this study does not have such surface coatings. Therefore, we omitted analysis of PA and LPA from our method as the quantification would not be reliably accurate or reproducible.

#### 3.2.1 HILIC method development for polar lipids

**Figure 3:**
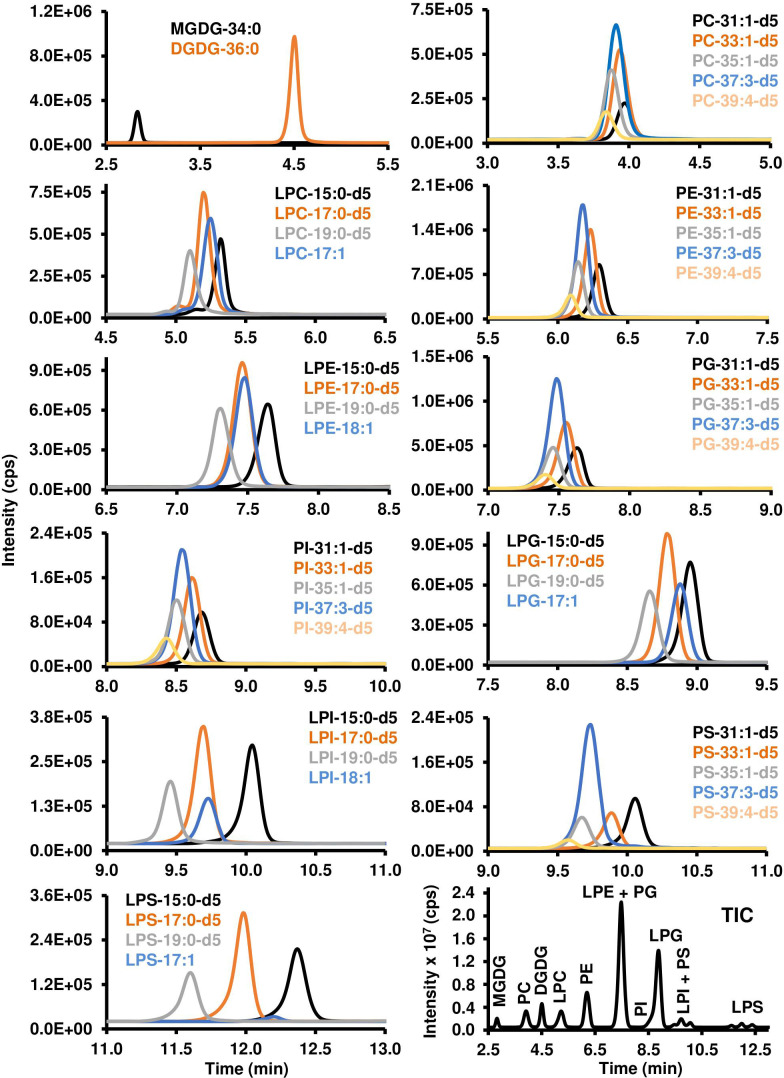
Chromatographic resolution and detection of polar lipids using LC-MS/MS. Polar lipids were detected and quantified using scheduled MRM scan survey as indicated in the *Materials and Methods*. XIC for each molecular species of lipid was generated using a precursor/product ion transition that is listed in **Tables 1** and **2**. TIC of the different classes of polar lipids is reported in the lower right corner of the figure. MGDG, monogalactosyldiacylglycerol; DGDG, digalactosyldiacylglycerol; PC, phosphatidylcholine; LPC, lysophosphatidylcholine; PE, phosphatidylethanolamine; LPE, lysophosphatidylethanolamine; PG, phosphatidylglycerol; PI, phosphatidylinositol; LPG, lysophosphatidylglycerol; LPI, lysophosphatidylinositol; PS, phosphatidylserine; LPS, lysophosphatidylserine. Each lipid class is characterized by its number of carbon atoms and double bonds. “d5” indicates the glycerol part of the lipid has five deuterium instead of hydrogen atoms.

Different chromatographic parameters, including temperature, solvents and additives, pH, and gradient steepness, were tested on the 12 classes of polar lipids. A gradient using acetonitrile/water/hexanes (92:6:2; v/v/v) and acetonitrile/water (50:50; v/v; pH 9.35), both containing 2 mM ammonium acetate, was able to separate the different classes of polar lipids with the exception of LPI and PS. The pH of the solvent was critical to elute LPI, LPS, and PS from the column, as any pH under 9.0 would not elute these lipid classes under the same gradient conditions. Overall, the separation of the polar lipid classes occurred within 13.0 minutes over a total of 17 minutes of chromatographic separation. The earlier eluting classes included those that were more positively charged or neutral: i) MGDG (2.8 min), ii) PC (3.9 min), iii) DGDG (4.5 min), and iv) LPC (5.1 min); while intermediate lipid classes included: v) PE (6.1 min), vi) LPE (7.4 min) and PG (7.4 min); and the more negatively charged lipid classes eluted later in the gradient: vii) PI (8.5 min), viii) LPG (8.7 min), ix) LPI (9.6 min) and PS (9.6 min), and x) LPS (11.9 min) (**Figure 3**).

The LOD and LOQ for most of the phospholipids and galactolipids ranged from 1 to 3 fmol and 3 to 10 fmol, respectively. PC was an exception with LODs between 1.9 to 6.1 fmol and LOQ between 6.3 to 20.2 fmol. These higher values for PC may be a reflection of the polar method performed using negative ionization mode where PC are net neutral due to the positive charge on the quaternary amine on the choline headgroup and negative charge on the phosphate group. The linearity for all calibration curves ranged from low pg/mL to high ng/mL with correlation coefficients over 0.98, demonstrating the robustness of this LC-MS/MS technique (**Table 3**).

**Table 3:** LC-MS/MS method sensitivity and linearity for non-polar and polar lipids.

Lipid	Transition (m/z)	RT (min)	Linearity Range (fmol)	R^2^	LOD (fmol)	LOQ (fmol)
MG-17:0	362.3/253.3	2.3	262.1-160,000.0	0.9990	78.7	262.1
MG-17:1	360.2/251.2	1.8	1,638.4-2,500,000.0	0.9952	492.0	1638.4
MG-19:0	390.3/355.3	2.9	65.5-250,000.0	0.9932	19.7	65.5
MG-19:1	388.3/279.1	2.3	131.1-500,000.0	0.9930	39.4	131.1
MG-19:2	386.3/277.1	1.9	26.2-250,000.0	0.9936	7.9	26.2
MG-21:0	418.3/383.3	3.9	81.9-50,000.0	0.9983	24.6	81.9
MG-22:1	430.3/321.3	3.2	26.2-250,000.0	0.9993	7.9	26.2
MG-24:1	458.3/349.3	3.9	65.5-100,000.0	0.9898	19.7	65.5
DG-31:1[D5]	575.5/332.2	5.2	14.7-22,395.0	0.9968	4.4	14.7
DG-33:1[D5]	603.4/316.4	6.0	28.0-42,650.0	0.9966	8.4	28.0
DG-35:1[D5]	631.6/344.2	7.1	16.0-24,424	0.9981	4.8	16.0
DG-37:3[D5]	655.4/368.4	6.4	4.1-15,670.0	0.9944	1.2	4.1
DG-39:4[D5]	681.6/332.2	6.7	4.9-18,820.0	0.9991	1.5	4.9
DG-44:2	750.7/395.4	12.3	13.1-20,000.0	0.9952	3.9	13.1
DG-48:2	806.7/423.4	15.9	5.2-20,000.0	0.9994	1.6	5.2
TG-41:0[D5]	731.4/486.4	11.4	0.5-1,750.0	0.9990	0.1	0.5
TG-43:1[D5]	757.4/512.4	11.5	0.9-3,375.0	0.9995	0.3	0.9
TG-45:1[D5]	785.5/540.6	13.2	0.5-4,880	0.9984	0.1	0.5
TG-47:1[D5]	813.4/540.4	15.3	0.7-2,512.0	0.9990	0.2	0.7
TG-49:1[D5]	841.4/568.4	16.9	0.8-3,032.0	0.9971	0.2	0.8
TG-51:2[D5]	867.4/594.4	17.2	0.6-2,352.0	0.9997	0.2	0.6
TG-53:3[D5]	893.8/594.6	16.7	0.5-1,712.0	0.9983	0.1	0.5
TG-54:9	890.8/595.6	9.7	1.3-5,000.0	0.9980	0.4	1.3
TG-55:4[D5]	919.6/620.6	17.0	0.3-2,770	0.9997	0.1	0.3
TG-57:4[D5]	947.8/648.6	18.7	0.4-1,345.0	0.9997	0.1	0.4
TG-66:3	1071.1/715.8	25.0	1.3-800.0	0.9977	0.4	1.3
TG-72:3	1155.4/771.8	26.7	1.3-2,000	0.9954	0.4	1.3
**MGDG-34:0**	**757.6/283.2**	**2.8**	**5.2-20,000**	**0.9910**	**1.6**	**5.2**
**DGDG-36:0**	**947.7/283.2**	**4.5**	**5.2-20,000**	**0.9942**	**1.6**	**5.2**
**LPC-15:0[D5]**	**545.4/241.2**	**5.3**	**5.4-20,548.0**	**0.9944**	**1.6**	**5.4**
**LPC-17:0[D5]**	**573.4/269.2**	**5.2**	**10.2-15,544.0**	**1.0000**	**3.1**	**10.2**
**LPC-19:0[D5]**	**601.4/297.3**	**5.1**	**4.8-18,424.0**	**0.9983**	**1.5**	**4.8**
**LPC-17:1**	**566.4/267.2**	**5.2**	**5.2-8,000**	**0.9987**	**1.6**	**5.2**
**LPE-15:0[D5]**	**443.4/241.2**	**7.7**	**5.9-8,998.4**	**0.9994**	**1.8**	**5.9**
**LPE-17:0[D5]**	**471.4/269.1**	**7.5**	**11.1-16,926.4**	**0.9984**	**3.3**	**11.1**
**LPE-19:0[D5]**	**499.4/297.3**	**7.3**	**5.2-19,972.0**	**0.9994**	**1.6**	**5.2**
**LPE-18:1**	**478.3/281.3**	**7.5**	**5.2-1,280.0**	**0.9993**	**1.6**	**5.2**
**LPG-15:0[D5]**	**474.3/241.1**	**9.0**	**5.2-8,040.0**	**0.9957**	**1.6**	**5.2**
**LPG-17:0[D5]**	**502.3/269.2**	**8.8**	**10.0-15,220.8**	**0.9998**	**3.0**	**10.0**
**LPG-19:0[D5]**	**530.4/297.2**	**8.7**	**4.7-7,228.8**	**0.9976**	**1.4**	**4.7**
**LPG-17:1**	**495.2/267.2**	**8.9**	**10.5-2,560.0**	**0.9976**	**3.1**	**10.5**
**LPI-15:0[D5]**	**562.4/241.2**	**10.1**	**4.5-43,050.0**	**0.9946**	**1.4**	**4.5**
**LPI-17:0[D5]**	**590.2/269.3**	**9.7**	**8.4-31,960.0**	**0.9991**	**2.5**	**8.4**
**LPI-19:0[D5]**	**618.4/297.2**	**9.5**	**4.1-15,704.0**	**0.9982**	**1.2**	**4.1**
**LPI-18:1**	**597.3/281.3**	**9.8**	**5.2-20,000.0**	**0.9996**	**1.6**	**5.2**
**LPS-15:0[D5]**	**487.4/158.0**	**12.4**	**5.1-19,588.0**	**0.9997**	**1.5**	**5.1**
**LPS-17:0[D5]**	**515.4/158.0**	**12.0**	**9.4-35,676.0**	**0.9998**	**2.8**	**9.4**
**LPS-19:0[D5]**	**543.3/158.0**	**11.7**	**4.6-17,648.0**	**0.9996**	**1.4**	**4.6**
**LPS-17:1**	**508.4/267.2**	**12.3**	**5.2-20,000.0**	**0.9980**	**1.6**	**5.2**
**PC-31:1[D5]**	**781.6/225.2**	**3.9**	**7.3-4,425.6**	**0.9989**	**2.2**	**7.3**
**PC-33:1[D5]**	**809.6/253.2**	**3.9**	**14.0-21,302.4**	**0.9981**	**4.2**	**14.0**
**PC-35:1[D5]**	**837.6/281.2**	**3.9**	**20.2-12,321.3**	**0.9912**	**6.1**	**20.2**
**PC-37:3[D5]**	**861.6/305.2**	**3.9**	**13.1-7,968.6**	**0.9879**	**3.9**	**13.1**
**PC-39:4[D5]**	**887.6/331.2**	**3.8**	**6.3-3,859.2**	**0.9981**	**1.9**	**6.3**
**PE-31:1[D5]**	**679.4/225.2**	**6.3**	**3.9-5,873.6**	**0.9996**	**1.2**	**3.8**
**PE-33:1[D5]**	**707.6/253.2**	**6.2**	**7.4-11,283.2**	**0.9918**	**2.2**	**7.4**
**PE-35:1[D5]**	**735.6/281.4**	**6.2**	**10.7-6,512.0**	**0.9981**	**3.2**	**10.7**
**PE-37:3[D5]**	**759.6/305.2**	**6.1**	**6.9-10,512.0**	**0.9935**	**2.1**	**6.9**
**PE-39:4[D5]**	**785.6/331.2**	**6.1**	**3.3-5,081.6**	**0.9995**	**1.0**	**3.3**
**PG-31:1[D5]**	**710.6/225.2**	**7.6**	**3.6-5,449.6**	**0.9955**	**1.1**	**3.6**
**PG-33:1[D5]**	**738.4/253.2**	**7.5**	**6.7-10,204.8**	**0.9977**	**2.0**	**6.7**
**PG-35:1[D5]**	**766.5/281.3**	**7.5**	**10.0-6,075.5**	**0.9904**	**3.0**	**10.0**
**PG-37:3[D5]**	**790.4/305.2**	**7.4**	**6.3-9,569.6**	**0.9952**	**1.9**	**6.3**
**PG-39:4[D5]**	**816.4/331.2**	**7.4**	**3.0-11,600.0**	**0.9982**	**0.9**	**3.0**
**PI-31:1[D5]**	**798.6/269.2**	**8.7**	**3.2-4,896.0**	**0.9990**	**1.0**	**3.2**
**PI-33:1[D5]**	**826.6/253.2**	**8.6**	**6.1-23,196.0**	**0.9948**	**1.8**	**6.1**
**PI-35:1[D5]**	**854.6/281.2**	**8.5**	**9.0-34,356.0**	**0.9985**	**2.7**	**9.0**
**PI-37:3[D5]**	**878.6/305.2**	**8.5**	**5.7-21,876.0**	**0.9947**	**1.7**	**5.7**
**PI-39:4[D5]**	**904.6/331.2**	**8.4**	**2.8-26,590.0**	**0.9959**	**2.1**	**7.0**
**PS-31:1[D5]**	**723.6/269.3**	**10.1**	**3.5-33,470.0**	**0.9990**	**1.1**	**3.5**
**PS-33:1[D5]**	**751.6/253.2**	**10.0**	**6.6-25,092.0**	**0.9999**	**2.0**	**6.6**
**PS-35:1[D5]**	**779.6/269.3**	**9.8**	**9.8-37,356.0**	**1.0000**	**2.9**	**9.8**
**PS-37:3[D5]**	**803.6/305.2**	**9.7**	**6.2-23,556.0**	**1.0000**	**1.9**	**6.2**
**PS-39:4[D5]**	**829.6/331.2**	**9.6**	**3.0-11,428.0**	**0.9998**	**0.9**	**3.0**

Limits of detection (LOD) and limit of quantification (LOQ) were obtained based on a signal-to-noise ratio of 3:1 and 10:1, respectively. Retention time (RT), linearity range, and correlation coefficient (R^2^) were also accessible using the current LC-MS/MS method. Polar lipids are depicted in bold.

#### 3.2.2 Reverse phase non-polar lipid method development

**Figure 4:**
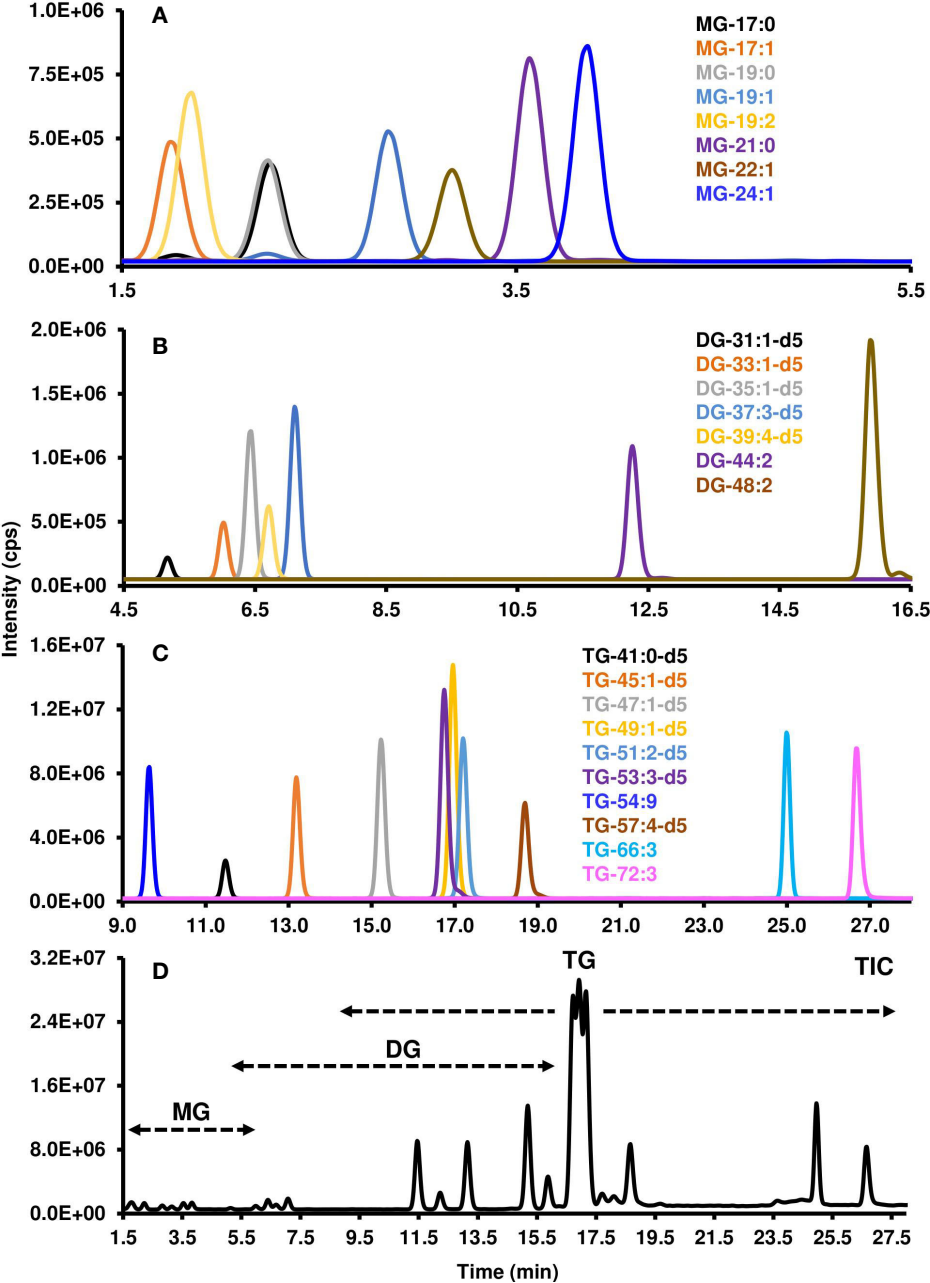
Chromatographic resolution and detection of non-polar lipids using LC-MS/MS. MRM scan survey was conducted to obtained the XIC of the different **(A)** MGs, **(B)** DGs, and **(C)** TGs. Each individual XIC represents a transition of precursor/product ion that was determined as indicated in the Materials and Methods as well as **Tables 1**, **2**. **(D)** Total Ion Count (TIC) which depicts the the total number of ions at a specific chromatographic time. MG, Monoacylglycerol; DG, diacylglycerol; TG, triacylglycerol. Each lipid class is characterized by its number of carbon atoms and double bonds. “d5” indicates the glycerol part of the lipid has five deuterium instead of hydrogen atoms.

As the non-polar lipid classes were separated using RP chromatography, the retention times of the molecular species varied based on their FA composition. The degrees of FA unsaturation and carbon length, along with lipid class, all contributed when each lipid would elute. The mobile phase solvent composition, steepness of the gradient, column temperature, and flow rate were all optimized to produce the greatest separation of non-polar lipid molecular species. Those species with greater FA unsaturation eluted earlier than those with less, and those with longer FAs eluted later than those with shorter FAs. The earliest eluting lipid class was MGs between 1.8 to 3.9 min (**Figure 4A**), then DGs between 5.2 to 15.9 min (**Figure 4B**), and finally TGs between 11.4 to 26.7 min (**Figure 4C** & **Table 3**). The entire run time for the method was 35 minutes (**Figure 4D**).

The standard curves for MGs, DGs, and TGs had correlation coefficients above 0.99 and showed strong linearity. The LC-MS/MS method was very sensitive for TGs with a LOD of 0.1-0.4 fmol and LOQ of 0.3-1.3 fmol for TG-41:0[D5], TG-45:1[D5], TG-53:3[D5], TG-54:9, and TG-55:4[D5], to LOD and LOQ up to 0.4 and 1.3 fmol for TG-72:3, respectively. The LC-MS/MS method was less sensitive for DGs and MGs which showed values that were higher by a factor of 10 and 30, respectively (**Table 3**).

Prior to determining the matrix effect (ME) and recovery efficiency (RE) for each non-polar and polar lipid class, five different extraction procedures were tested in order to select one that would provide the best coverage for the lipid classes of interest, as discussed in the following section.

### 3.3 Unraveling the best extraction method for non-polar and polar lipids

Many considerations go into potential extraction methods for lipids, such as efficiency of extraction for different lipid classes, length of time or number of steps to extract, toxicity of solvents used, cleanliness of the extract prior to analysis, and suppression of phospholipase D (PLD) activation that can hydrolyze phosphatidylcholine to produce phosphatidic acid. Here, we tested five different extraction protocols on leaf and seed tissues, analyzing both polar and non-polar lipids, and assessed each protocol based on the criteria described above. The rationale for testing the leaf and seed tissues was that they are known to have different lipid composition: e.g. leaves are photosynthetic organs containing galactolipids which are minimal in seeds (Reszczyńska and Hanaka, 2020). The five methods tested, described in the Materials and Methods section, include a hexane/isopropanol based extraction (Method 1), two isopropanol/chloroform based extractions (Methods 2 & 3), a butanol/methanol based extraction (Method 4), and a MTBE based extraction (Method 5). The difference between the two isopropanol/chloroform based extractions is that Method 2 (adapted from Shiva et al., 2018) has a longer extraction time while Method 3 (adapted and modified from Chapman and Moore, 1993) has an added KCl wash step that helps to clean samples of debris and proteins.

In terms of efficiency of extraction, we assessed this based on the total LCMS peak area per tissue relative to the other extraction methods. Overall each method was similar in their ability to extract the various different lipid classes. Method 5 (MTBE) extracted PC, PE, and PI slightly better than the other methods in leaf tissue (2.7E6 ± 2.6E5, 8.8E6 ± 4.9E5, 1.3E6 ± 6.0E4 peak area/mg, respectively), and slightly better for PC in seed tissue (5.3E6 ± 1.2E5 peak area/mg) (**Supplementary Table 4**). Method 1 (hexane/isopropanol) had the least efficient extraction for nearly all lipid classes in leaf and seed tissues, and had the greatest variability between replicates relative to the other extraction methods (average RSD of 30.3% in seeds and 23.2% in leaf compared to 9-12% in seeds and 10-12% in leaf for the other methods). All of the methods performed similarly with DGs and TGs, though Method 4 (butanol/methanol) had greater variability than the others (RSD of 21.2% for DGs and 32.8% for TGs).

Assessing the methods on the length of time and ease of extraction, we found that each of the methods required a similar amount of hands-on time by the researcher. Overall Methods 1 and 4 were the fastest, as they had no incubation steps, whereas Method 2 had a 24 h incubation step and Method 3 had a 30 min and an overnight incubation steps in addition to an added washing procedure. Method 5 had a 1 h incubation, and was therefore intermediate between the other methods.

The toxicity of solvents used in extraction is an important consideration for the health of those in the lab and the disposal of extracts following analysis. Here, we considered Methods 1, 4, and 5 to be superior compared to Methods 2 and 3 for avoiding the use of chloroform, a known carcinogen (Davidson et al., 1982). The final criteria in selecting an extraction method was the cleanliness of the samples, as any remaining tissue debris and particulate matter can cause blockage to LC columns, resulting in high back pressure, creating additional wear on the pumps and affect peak retention time and shape. Method 3 has a dedicated washing step using KCl that is meant to aid in removing proteins and carbohydrates, which helps to clear the extract of anything non-lipid related. Methods 4 and 5 have the additional benefit of being biphasic extractions that separate into an aqueous and nonpolar phase with the nonpolar phase on the top so that the extract can be aspirated off without penetrating an interphase that may contain tissue debris. Methods 1 and 2 were the only methods to lack a biphasic separation or a washing step, but rather relied on centrifugation to pellet the homogenate.

A final consideration to take into account when choosing a lipid extraction method is the suppression of PLD activation. PLD hydrolyzes PC into choline and PA during wounding or stress to act as secondary messengers within plants (Munnik, 2001). Likewise, PLD can be activated during lipid extraction which could reduce PC content and produce incorrect quantification. Isopropanol will inhibit such enzyme activity (Chalifa-Caspi et al., 1998) and will therefore minimize the risk of post-extraction degradation of lipids. This is an advantage of Methods 1, 2, and 3, which all contain isopropanol. However, there was no decrease in PC content in Methods 4 or 5 relative to the isopropanol containing extraction methods to suggest PLD activation. Therefore inactivation of PLD did not appear to be a major factor in deciding which extraction method to proceed with. However, researchers should take caution to reduce PLD activity by always halting all metabolic activity in harvested tissue by immediately freezing in liquid nitrogen prior to lipid extraction.

In consideration of all the above criteria we selected Method 5, the MTBE based extraction, as the preferred method as it had a slightly greater extraction efficiency relative to the other methods, was not time intensive, avoided the use of more toxic solvents, and had cleaner extracts prior to analysis.

Using Method 5 we extracted lipids from pennycress seed and leaf tissues to show the differences between the major phospholipids and their FA compositions, and demonstrate the effectiveness of the extraction method and the developed LC-MS/MS methods (**Supplemental Figure 1**). In all of the phospholipid classes, leaf tissue showed a higher relative amount of linoleate (18:2) and linolenate (18:3) whereas seed tissue was more abundant in oleic (18:1) and linoleate (18:2). For example, the most abundant PC in seed tissue was PC 36:2 (18:1_18:1) (1.1E6 ± 3.0E5 peak area/mg) compared to PC 34:3 (18:3_16:0) (9.3E5 ± 1.3E5 peak area/mg) in leaf tissue (**Supplemental Figure 1A**). Similarly, the most abundant PE molecular species in leaf tissue was PE 34:3 (18:3_16:0) (2.7E6 ± 1.9E5 peak area/mg), but in seed tissue was PE 36:4 (18:2_18:2) (2.5E6 ± 1.2E5 peak area/mg) (**Supplemental Figure 1B**). The same FA combination is found for PI in leaf tissue (PI 34:3 (18:3_16:0), 6.6E5 ± 5.0E4 peak area/mg), but for seed was PI 34:2 (18:2_16:0) (1.1E6 ± 5.4E4 peak area/mg) (**Supplemental Figure 1C**). Unlike the other phospholipids, the most abundant PG molecular species for both seed and leaf tissue were PG 34:2 (18:2_16:0) (1.0E6 ± 1.3E5 peak area/mg for seed, 1.7E6 ± 1.1E5 peak area/mg for leaf) and PG 34:1 (18:1_16:0) (3.1E5 ± 4.6E4 peak area/mg for seed, 1.1E6 ± 6.1E4 peak area/mg for leaf) (**Supplemental Figure 1D**). Values for each of the lipid classes and tissues tested are found in **Supplementary Table 4**.

### 3.4 Method validation using RE and ME

To validate the application of the two LC-MS/MS methods, pennycress mature seeds were spiked, before or after extraction, with UltimateSPLASH lipidomics mix and hydrogenated galactolipids (MGDG and DGDG) in order to determine ME and RE for each of those compounds (*Materials and Methods*). The ME for the non-polar lipids contained in the UltimateSPLASH lipidomics mix, DG-XX : X[D5] and TG-XX : X[D5], were in the acceptance range of ± 25%, suggesting that there was no significant effect of the matrix extracted from pennycress mature seeds (**Table 2**). In contrast, six of the twenty-two polar lipid standards (MGDG-34:0, LPE-19:0[D5], PC-31:1[D5], PC-35:1[D5], PE-31:1[D5], PE-35:1[D5]) used to test ME showed significant negative values below the -25% threshold, which indicated ion suppression from mature pennycress seed extracts (**Table 2**).

RE for DGs and TGs were greater than 79% for all of the standards tested, underlining a good recovery for the non-polar lipids. Recovery for the polar lipids was efficient and above 75.9% for MGDG, DGDG, PC, PE, PG, PI, PS, LPC, and LPE, but under the 75% threshold for the LPG, LPI, and LPS classes (**Table 2**).

Overall, the ME and RE for the different classes of non-polar and polar lipids validated both LC-MS/MS methods that can be applied to a variety of plant organs. One last confirmation for accurate determination of FA composition within TG and for accurate quantification was required prior analysis of biological samples, as discussed below.

#### 3.4.1 TG-FA confirmation using LC-MS/MS acquisition dependent information

As the quantification of TGs relied on overlaying individual XIC of TG-FA MRM transitions (see **Figure 2**), we decided to validate this approach by performing an information dependent acquisition (IDA) experiment using MRM as the first scan survey followed by an enhanced product ion (EPI) scan step to produce the product ion spectrum of the parent TG considered. This LC-MRM-EPI-MS approach used the same LC and source parameters as the non-polar lipid method developed and validated above. The EPI showed the fragmentations of the expected FAs for the TG molecular species with the predicted FA combination. For example, the EPI scan for the MRM of TG 62:3 – FA 18:1 depicted fragmentations of FA 22:1 and FA 18:1, suggesting a final FA combination of 18:1_22:1_22:1, while the EPI scan for TG 62:3 – FA 20:1 displayed fragmentations of a variety of FAs at a much lower intensity relative to TG 62:3 – FA 18:1, many of which were very long chain FAs (**Supplemental Figure 2**).

#### 3.4.2 Comparison of TG FA mol% relative to mature seed FA mol%

To determine how well our strategy in selecting individual TG-FA MRMs in the non-polar method performed, we compared the FA mol% of the TG content of mature seeds as measured using our non-polar method relative to GC-MS analysis of FAMEs taken from pennycress seeds of the same accession. We found that while there are some discrepancies, e.g. lower erucic acid content (30.7% vs. 36.2%) and higher oleic acid content (18.5% vs. 15.7%) in our TG measurements, the overall FA mol% profile is similar between the two sets of measurements (**Supplemental Figure 3**).

### 3.5 Plant targeted lipidomics workflow

**Figure 5:**
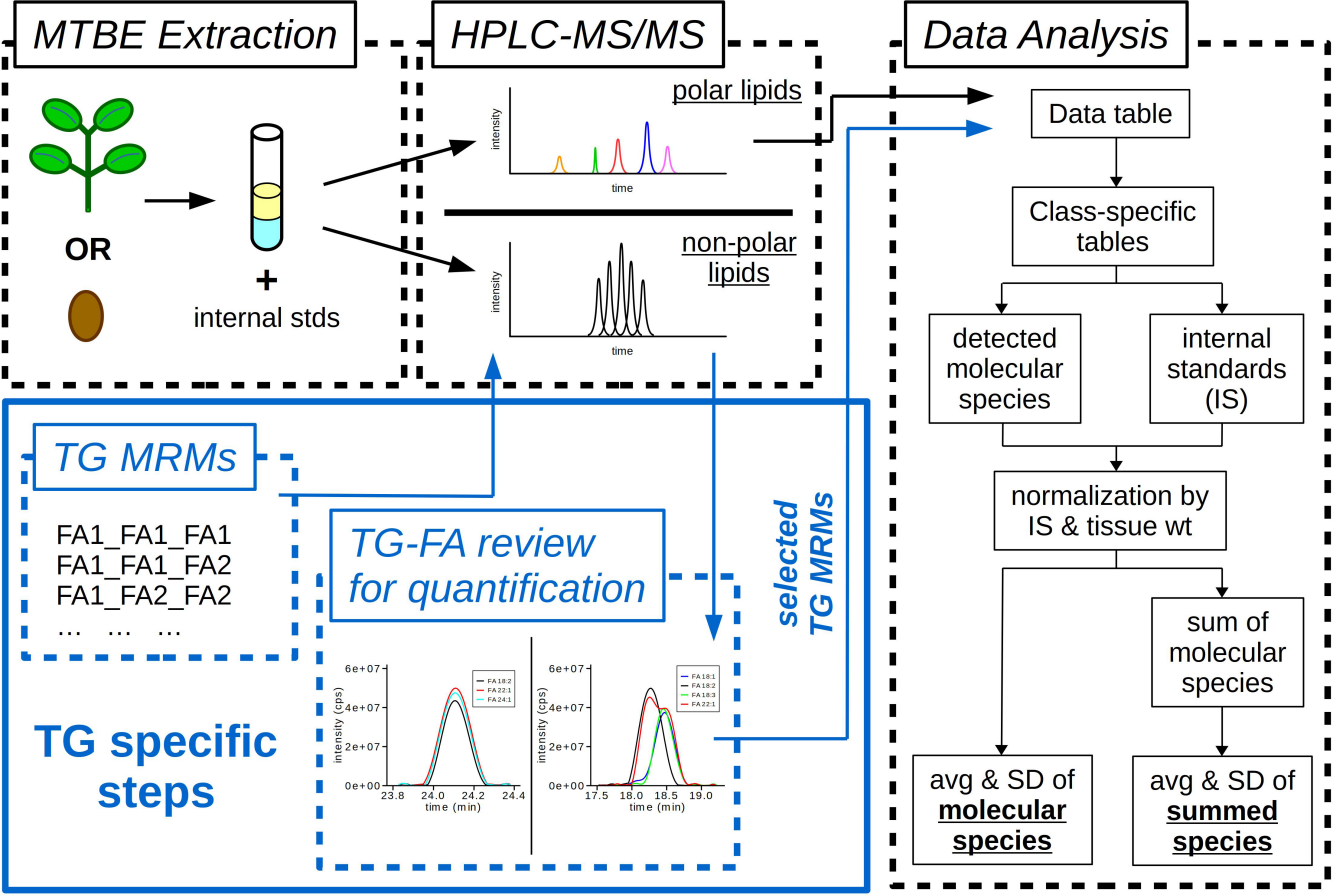
Workflow of lipidomics methods. Samples (leaf or seed) extracted using a methyl-tert-butyl ether (MTBE) extraction. During extraction samples are spiked with internal standards (IS) containing both polar and non-polar lipids. Final extracts are diluted and resuspended in appropriate solvents for two different HPLC-MS/MS methods, one for polar lipids and the other for non-polar lipids. Analysis of triacylglycerols (TG) requires additional steps to determine fatty acid (FA) composition. TG MRMs are calculated for each potential FA composition. TG-FA extracted ion chromatograms (XIC) are overlaid for each TG molecular species to determine the MRM transitions to use for data analysis and quantification. Custom R scripts quantify the integrated LC peak areas for each lipid class, then normalize the data by tissue weight (wt) and IS. The final output is the average (avg) and standard deviation (SD) for each molecular species of each lipid class and the avg and SD of the summed lipid classes.

The final workflow for the targeted lipidomics approach developed in this study is outlined in **Figure 5**. In brief, plant tissues are extracted using an MTBE-based method (Method 5) to which a mixture of internal standards is added prior to extraction. The plant extracts are then prepared and diluted in appropriate solvents for the analysis of polar and non-polar lipids by LC-MS/MS using HILIC (NH_2_ column) and RP (C30 column) separation technology, respectively. Prior to the data analysis of the non-polar lipids, a preceding step is required for TGs to account for all of the possible FA combinations for each of the isobaric TG molecular species. MRMs are calculated for each TG precursor ion and each potential FA contributing to the precursor ion molecular mass, and then TGs are monitored as pairs of precursor/product ions during the LC-MS/MS run. Following LC-MS/MS analysis, the polar lipids data are directly analyzed using Multiquant software. The TG data are first reviewed in Analyst software for the different possible combinations of FAs that can be shown to account for the TG precursor ion mass. Specific TG-FA MRMs are selected for the data analysis step representing the TG profile of the extract and used for quantification. This step eliminates overestimating TG content by removing MRM transitions that are from the same TG precursor ion (e.g. removing both TG 54:2 – FA 16:0 and TG 54:2 – FA 20:1 transitions but keeping TG 54:2 – FA 18:1 for quantification of TG 54:2 (16:0_18:1_20:1)). Analysis of the polar and non-polar lipid data is done using a custom written R script (**Supplemental Files 2, 3**). Data from tab-delimited text files are imported into R as data frames. Lipid classes are separated into individual data frames from which integrated peak areas are normalized by tissue weight and internal standard amount. The total lipid class average and standard deviation are calculated along with the average and standard deviation for each individual molecular species.

### 3.6 Application of polar and non-polar lipid LC-MS/MS methods to biologically relevant samples

To demonstrate the application of the lipidomics methods developed here, we analyzed the lipid extracts from developing pennycress seeds, mature seeds, and seedlings. During seed development, seeds synthesize an abundance of TGs which are stored in lipid droplets until their use during germination as a carbon and energy reserve (Ischebeck et al., 2020). Biosynthesis of TGs in plants utilizes two metabolically distinct sources of DGs―*de novo* DG and PC-derived DG. *De novo* DG is produced by the Kennedy pathway (Kennedy, 1961), where glycerol-3-phosphate is sequentially acylated by glycerol-3-phosphate acyltransferase (GPAT), followed by lysophosphatidic acid acyltransferase (LPAT) to produce phosphatidic acid (PA) (Shockey et al., 2016). The phosphate headgroup is removed by phosphatidic acid phosphatase (PAP), producing DG which can be acylated with a final acyl-CoA by DG acyltransferase (DGAT) (Routaboul et al., 1999; Chen et al., 2022). Alternatively, DG can be acylated using an acyl group from PC by the action of phospholipid:DG acyltransferase (PDAT) (Bates, 2016). PC-derived DG is generated by the removal of the PC headgroup by phosphatidylcholine:diacylglycerol cholinephosphotransferase (PDCT) (encoded by the *ROD1* gene) (Lu et al., 2009), and this PC-derived DG can be acylated to form TG by either DGAT or PDAT. Acyl groups in PC can be modified by desaturases, such as *fatty acid desaturase 2* (FAD2) and FAD3, and/or they can enter and exit from PC by exchanging with the acyl-CoA pool by acyl editing reaction (sometimes referred to as the Lands pathway) (Kennedy, 1961; Lands, 1965) that produce lysophosphatidylcholine (LPC) as an intermediate in the process (Lager et al., 2013). Acyl-CoAs can be modified by elongation through *fatty acid elongase 1* (FAE1), which in pennycress produces a large amount of the very long chain fatty acid erucic acid (22:1) through two cycles of FA elongation beginning with oleoyl-CoA.

Using our lipidomics methods we focused on the major lipid classes involved in TG biosynthesis, namely PC, DG, TG, and LPC.

**Figure 6:**
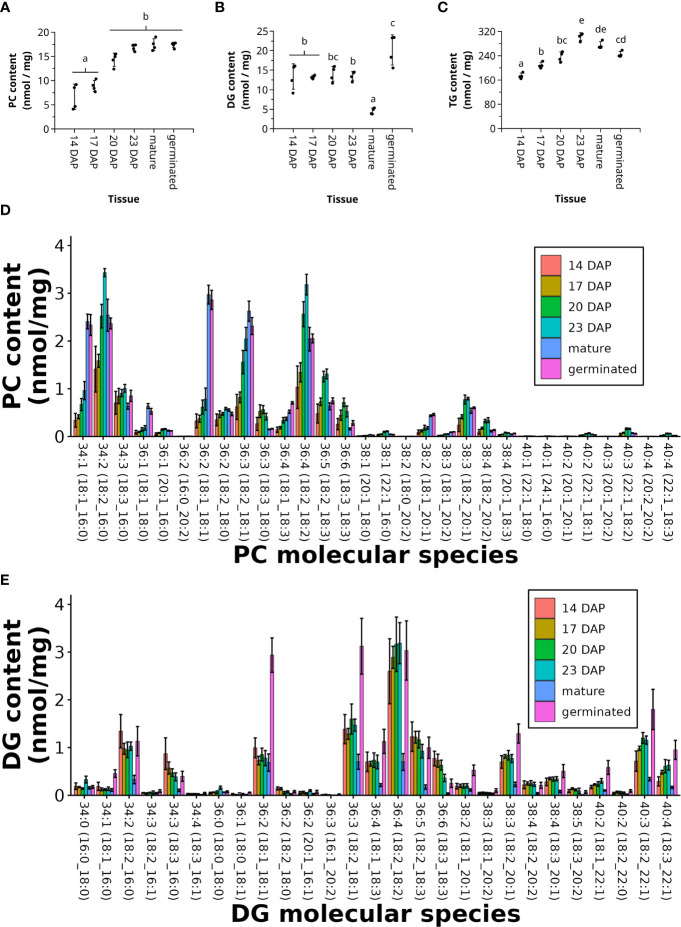
Lipidomics of developing pennycress seeds. Total sum of PC **(A)**, DG **(B)**, and TG **(C)**, and the individual molecular species of PC **(D)** and DG **(E)** from developing pennycress seeds at 14, 17, 20, and 23 days after pollination (DAP), as well as mature and germinated seeds. (n = 4; one-way ANOVA; Tukey test, *p* < 0.01).

PC is a central metabolite within TG biosynthesis during seed development, acting as a source of acyl chains to acylate DG *via* PDAT or as the substrate for FA desaturation. The total amount of PC increased from 6.7 ± 2.6 nmol/mg DW and 8.9 ± 1.1 nmol/mg DW at 14 and 17 DAP, respectively, to nearly doubling at maturity with 17.5 ± 1.2 nmol/mg DW (**Figure 6A**). PCA of the FA composition in PC molecular species showed that mature and germinated pennycress seeds group together away from the other developmental stages (**Supplemental Figure 4A**). Additionally, the younger stages (14 and 17 DAP) cluster together, away from the older ones (20 and 23 DAP). Indeed, the FA composition of PC molecular species during the height of seed oil synthesis at 20 and 23 DAP, showed the greatest diversity of FAs with both polyunsaturated FAs (≥ 2 FA double bonds) and elongated FAs (> 18 C in FAs). The most abundant PC molecular species at 20 and 23 DAP relative to the other time points were PC 34:2 (18:2_16:0), PC 36:4 (18:2_18:2), PC 36:5 (18:2_18:3), and PC 38:3 (18:2_20:1) (**Figure 6D** and **Supplemental Figures 5B**).

A key substrate for TG synthesis is DG, used either in the acyl-CoA dependent pathway with DGAT or in the acyl-CoA independent pathway with PDAT. For much of the seed developmental stages the total amount of DG remained consistent, between 13.3 and 14.0 nmol/mg DW, while the amount of DG dropped considerably to 4.5 ± 0.8 nmol/mg DW in mature seeds (**Figure 6B**). Once the seeds began germinating there was a large abundance of DGs, 20.2 ± 3.9 nmol/mg DW, likely indicating the breakdown products of TGs (**Figure 6B**). PC2 separated the FA composition in DG molecular species in developing pennycress seeds from mature and germinated stages (**Supplemental Figure 4B**). Developing pennycress seeds (14 to 23 DAP) grouped together whereas PC1 separated the mature and germinated ones. There appeared to be little variation in the FA composition in DGs when comparing developing seeds, mature seeds, or germinating seeds, but the most abundant DG molecular species were DG 36:4 (18:2_18:2), DG 36:3 (18:2_18:1), and DG 36:5 (18:2_18:3) (**Figure 6C**).

Because LPC is an intermediate between the PC pool and the acyl-CoA pool used for TG biosynthesis, we quantified the molecular species of LPCs at the various stages of seed development. The total LPC content for all samples was less than 1 nmol/mg DW, but was greatest at 20 DAP with the most abundant molecular species being LPC 18:2 (**Supplemental Figure 6A, B**).

**Figure 7:**
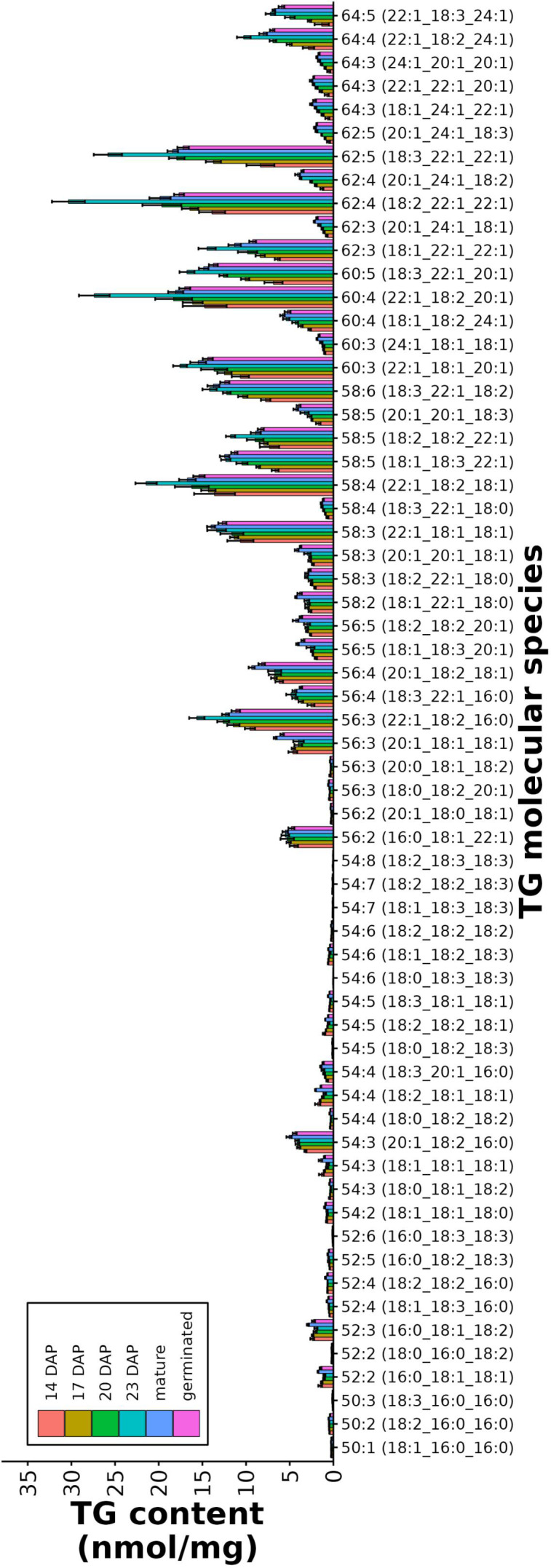
Molecular species of TG from developing pennycress seeds. Individual molecular species of TG from developing pennycress seeds at 14, 17, 20, and 23 DAP, as well as mature and germinated seeds.

The final storage product (TGs) increased throughout seed development, beginning at 180.2 ± 8.2 nmol/mg DW at 14 DAP and ending to approximately 300 nmol/mg DW at 23 DAP and maturity. During germination TG content began to decrease, corresponding to TG breakdown (**Figure 6C**). Using the C30 core-shell column to separate out individual TG molecular species, and our approach aligning different TG-FA MRM transitions, we were able to determine the FA composition of the different TG molecular species. PCA of the FA composition in the TG molecular species showed a clear separation between all the different stages: PC1 separated 14, 17, 20, and 23 DAP whereas PC2 separated mature and germinated seeds (**Supplemental Figure 4C**). The composition of different TG molecular species did not differ much between the different time points of seed development, or at maturity or germination. The most abundant TG molecular species were TG 62:4 (18:2_22:1_22:1), TG 62:5 (18:3_22:1_22:1), TG 60:4 (22:1_18:2_20:1), and TG 58:4 (22:1_18:2_18:1), all of which contain at least one erucic acid, with the most abundant molecular species containing two erucic acids (**Figure 7**). TG molecular species containing erucic acid were most abundant at 23 DAP, just prior to maturity, whereas the TG molecular species containing 18 and 20C FAs were more abundant at maturity relative to the other time points (**Figure 7**).

## 4 Discussion

In this study, we have developed a comprehensive targeted lipidomics approach using LC-MS/MS analysis and MRM scan survey that is able to evaluate molecular species of both polar and non-polar lipid classes from plant tissues. Here we have used these methods to determine the polar and non-polar lipid content from pennycress seeds and seedlings, as well as to assess the complete FA compositions from all lipid classes, including TGs. Our polar lipid LC-MS/MS method effectively separated polar lipid classes by HILIC mode, and detects each molecular species as a deprotonated acyl side-chain in negative ionization mode. In contrast, our non-polar lipid LC-MS/MS method separates molecular species *via* their FA characteristics, i.e. FA carbon length and unsaturation, using RP chromatography mode and positive ionization mode. In particular, the C30 RP column allowed the determination of the acyl side-chain composition of TG molecular species, including isobaric and isomeric species. Other studies have used C30 RP chromatography as a means to improve upon LC peak resolution, and thereby increase the number of features per LC-MS/MS analysis to gain a more complete view of the lipidome (Narváez-Rivas and Zhang, 2016; Rampler et al., 2018; Pham et al., 2019; Jankevics et al., 2021). The MRM-based LC-MS/MS approach described here demonstrates this same high peak capacity separation of C30 RP chromatography by resolving isomers of TGs that may overlap in typical C8 or C18 RP chromatography. With an intermediate step prior to data analysis, choosing specific TG-FA MRM transitions for the actual quantification, we can: i) determine the FA composition of each unique molecular species, and ii) quantify TG using a single TG-FA MRM transition while accounting for TG molecular species that may have more than one of the same acyl chain. Thus, these two LC-MS/MS methods combined together provide a more complete and exhaustive view of the lipidome that is not always trivial in a single-run method.

Both polar and non-polar lipid LC-MS/MS methods displayed good LOD, LOQ, and linearity, with values as low as the fmol range for most of the lipid classes. However, the LOD and LOQ values for MGs were in the hundreds of fmol range, which may reflect the difficulty to ionize and detect this lipid class. Indeed, MGs were not detectable in our biological samples, possibly due to poorer ionization and also its lower abundance in the lipidome.

Considering that a LC-MS/MS method relies on the efficacy of a specific extraction method, five different extraction methods were tested to optimize the coverage of lipid classes. The assessment of these five extractions was based on criteria such as efficiency, time, chemical toxicity, and the cleanliness of extracts prior to analysis. Overall, the efficiency was similar for most extraction methods, and therefore not a predominant factor in selecting an adequate extraction method. For the other criteria assessed, the MTBE-based extraction method (Method 5) was ultimately chosen because it required less time than the other methods, avoided the use of chloroform, and produced cleaner extracts due to its phase separation of the organic and aqueous layers, with the organic phase as the upper phase. The LC-MS/MS lipidomics methods were validated using pennycress mature seeds by calculating ME and RE after adding a specific internal standard mixture of different lipid classes before and after extraction. ME and RE values were within a ± 25% range for most of the lipids (**Table 2**).

This lipidomics workflow was applied to pennycress developing, mature, and germinating seeds to determine the state of the lipidome during rapid TG biosynthesis, up to its storage at maturity, and then when it begins to breakdown during post-germinative growth. In particular, pennycress has a unique seed oil FA composition, containing an abundance of the very long chain FA (VLCFA) erucic acid (22:1), as well as polyunsaturated FAs (PUFA), such as linoleic (18:2) and linolenic (18:3) acids. These two FA modifications are routed through two different pathways. FAs to be elongated use acyl-CoA as a substrate for elongase enzymes, such as FAE1, while FAs to be desaturated use PC as a substrate for desaturase enzymes, such as FAD2 and FAD3. In addition to these two FA modification pathways, there are two main enzymes that synthesize TGs, the acyl-CoA dependent DGAT and the acyl-CoA independent PDAT. DGAT uses DG and acyl-CoA as its substrates to synthesize TG, while PDAT uses PC as its acyl donor to DG. As the FA composition of pennycress contains a wide diversity of FAs, among which VLCFAs (> 50%) and PUFAs (~ 35%) are highly represented, it was not clear how important each of these classes of lipids and what the metabolic pathways were for TG biosynthesis. Previously it was shown that there is heterogeneity in the distributions of TG molecular species in pennycress embryos with VLCFA-containing TGs in the cotyledons and PUFA-containing TGs in the embryonic axis, suggesting a spatial separation of metabolic routes to TG (Jarvis et al., 2021). Here, seed tissues were extracted together and so “blur” the heterogeneity of these different lipid compositions and the lipidomes of different seed tissues.

The PC content in developing seeds remained relatively constant at 14 and 17 DAP, but doubled at 20 and 23 DAP. Interestingly, it was at these later stages of seed development that the FA composition of PC increased in diversity, containing both VLCFAs and PUFAs. However, at maturity and during germination the FA composition was less diverse and constituted mostly of oleic (18:1), stearic (18:0), and palmitic (16:0) acids. These changes in PC composition support that PCs play an important role in “channeling” VLCFAs and PUFAs into TGs during pennycress seed development similar to the situation reported for other Brassicaceae species (Lu et al., 2009; Bates and Browse, 2011; Yang et al., 2017; Bai et al., 2020; Bhandari and Bates, 2021; Pollard and Shachar-Hill, 2022), and as also was inferred from previous studies with pennycress mutants (Jarvis et al., 2021). Given that erucic acid (22:1) is a product of the FA elongation pathway, PCs might not necessarily be suspected as a key player in TG biosynthesis in pennycress; however, it is most probable that erucic acid is channeled back into PC following elongation, and from there it is incorporated into TG by PC-derived DG following the action of PDCT or *via* acyl transfer into TG from PC by PDAT. This is also the probable route for PUFAs to enter TG that are desaturated on PC. These metabolic scenarios are consistent with our observed rise and fall of VLCFAs and PUFAs in PC molecular species, and continue to point to PC as an important intermediate for TG synthesis during the progression through pennycress seed development.

LPCs, products of PDAT when synthesizing TGs, had a low abundance throughout seed development, which could suggest a rapid turnover of FAs from PC through a quick succession of deacylation and reacylation. Alternatively, or in addition to PDAT, FAs can also be cleaved from PC through LPC acyltransferase (LPCAT) or phospholipase A (PLA) to give rise to acyl-CoAs. Since the most abundant LPC contained 18:2, this could indicate other more modified FAs, such as erucic acid (22:1), gondoic acid (20:1), and linolenic acid (18:3) seen in PC at 20 and 23 DAP, are more rapidly exchanged through PC into the acyl-CoA pool or TG, rather than retained on LPC. Alternatively, LPC-18:2 is more abundant because the LPCATs prefer LPC containing 22:1, 20:1, 18:3 to resynthesize PC or that the LPC producing enzymes (PLA & LPCAT) are less likely to use PC molecular species containing these fatty acids.

DG levels remained relatively stable between 14 and 23 DAP, unlike PCs. This may imply DGs are actively synthesized or turned over during seed development as TG accumulation occurs. Once developing seeds reached maturity, the DG content decreased to about a third of its level seen during development, which may be explained by its incorporation into PCs and TGs. During seed germination DG levels increased, which is likely a result of TG breakdown. We also observed that the FA composition of the most abundant DGs during seed development from 14 to 23 DAP had minimal variations. The most abundant DG molecular species were DG 36:4 and DG 36:3, containing 18:2_18:2 and 18:2_18:1 FAs, respectively. This finding is somewhat surprising compared to the FA composition of TGs where the most abundant molecular species contain at least one to two erucic acids (22:1), or gondoic acid (20:1^Δ11^). To attain that FA composition within TGs, these DG molecular species would need to have at least one of their FAs removed, which supports a route through PC and acyl editing, or an uncharacterized transacylase activity. Alternatively, and perhaps more likely, is that erucic acid containing DG molecular species are selectively incorporated into TG. Recent work (Jeppson et al., 2019; Lager et al., 2020) indicates that DGAT enzymes can be highly selective for DG molecular species, including those containing erucic acid. Thus, erucic acid containing DGs may be more quickly utilized to make TG than other DGs, leaving other DGs to accumulate. An additional alternative possibility would be that TG biosynthesis incorporates 18:2 containing DGs, but that TG remodeling after initial synthesis accounts for ultimate TG compositions higher in erucic acid (Bhandari and Bates, 2021).

By contrast, the FA composition of DGs from seedlings contains a relatively higher amount of VLCFAs compared to developing seeds, though by far the highest are the same DG 36:4 and DG 36:3 indicated above. This could be an indication of TGs containing VLCFAs being preferentially used in beta-oxidation for energy as seedlings grow and establish.

These subtleties of the FA compositions of the multiple lipid classes, both the polar lipids and non-polar lipids, enlighten the understanding of TG biosynthesis in pennycress, an important first step in the ultimate goal of engineering pennycress seed oil for different nutritional and biotechnological uses. They also highlight the advantages of our stepwise lipidomics approach described here using two LC-MS/MS methods to separate out the polar lipids and non-polar lipids by their FA characteristics. As analytical technologies advance, even more subtle features of lipid biosynthesis will become unraveled and aid in future endeavors for engineering seed oils.

## Data availability statement

The original contributions presented in the study are included in the article/**Supplementary Material**. Further inquiries can be directed to the corresponding authors.

## Author contributions

TR, J-CC, AA, and KC designed and conceptualized the project and method development; TR. and J-CC prepared pennycress plant materials, and lipid extractions; TR, J-CC, and MP performed LC-MS/MS method development; TR and J-CC performed method validation; TR analyzed data, and created data analysis workflow; TR, J-CC, AA, and KC drafted the manuscript; AA, and KC supervised and coordinated the project; AA, and KC acquired funding for the project. All authors contributed to the article and approved the submitted version.

## Funding

This work was supported by the Department of Energy Office of Science, Office of Biological and Environmental Research (BER), grant # DE-SC0020325.

## Acknowledgments

We acknowledge the BioAnalytical Facility at the University of North Texas for the support with the LC-MS/MS analyses.

## Conflict of interest

The authors declare that the research was conducted in the absence of any commercial or financial relationships that could be construed as a potential conflict of interest.

## Publisher’s note

All claims expressed in this article are solely those of the authors and do not necessarily represent those of their affiliated organizations, or those of the publisher, the editors and the reviewers. Any product that may be evaluated in this article, or claim that may be made by its manufacturer, is not guaranteed or endorsed by the publisher.

## References

[B1] Bai S. Wallis J. G. Denolf P. Engelen S. Bengtsson J. D. Van Thournout M.. (2020). The biochemistry of headgroup exchange during triacylglycerol synthesis in canola. Plant J. 103, 83–94. doi: 10.1111/tpj.14709 31991038 PMC7605783

[B2] Bates P. D. (2016). Understanding the control of acyl flux through the lipid metabolic network of plant oil biosynthesis. Biochim. Biophys. Acta BBA Mol. Cell Biol. Lipids 1861, 1214–1225. doi: 10.1016/j.bbalip.2016.03.021 27003249

[B3] Bates P. D. Browse J. (2011). The pathway of triacylglycerol synthesis through phosphatidylcholine in arabidopsis produces a bottleneck for the accumulation of unusual fatty acids in transgenic seeds: PC is a bottleneck in HFA-TAG synthesis. Plant J. 68, 387–399. doi: 10.1111/j.1365-313X.2011.04693.x 21711402

[B4] Best K. F. Mcintyre G. I. (1975). The biology of Canadian weeds: 9. *Thlaspi arvense* l. *Can* . J. Plant Sci. 55, 279–292. doi: 10.4141/cjps75-039

[B5] Bhandari S. Bates P. D. (2021). Triacylglycerol remodeling in *Physaria fendleri* indicates oil accumulation is dynamic and not a metabolic endpoint. Plant Physiol. 187, 799–815. doi: 10.1093/plphys/kiab294 34608961 PMC8491037

[B6] Bligh E. G. Dyer W. J. (1959). A rapid method of total lipid extraction and purification. Can. J. Biochem. Physiol. 37, 911–917. doi: 10.1139/o59-099 13671378

[B7] Cahoon E. B. Li-Beisson Y. (2020). Plant unusual fatty acids: learning from the less common. Curr. Opin. Plant Biol. 55, 66–73. doi: 10.1016/j.pbi.2020.03.007 32304939

[B8] Cajka T. Fiehn O. (2014). Comprehensive analysis of lipids in biological systems by liquid chromatography-mass spectrometry. TrAC Trends Anal. Chem. 61, 192–206. doi: 10.1016/j.trac.2014.04.017 PMC4187118 25309011

[B9] Cavaco A. R. Matos A. R. Figueiredo A. (2021). Speaking the language of lipids: the cross-talk between plants and pathogens in defence and disease. Cell. Mol. Life Sci. 78, 4399–4415. doi: 10.1007/s00018-021-03791-0 33638652 PMC11073031

[B10] Chalifa-Caspi V. Eli Y. Liscovitch M. (1998). Kinetic analysis in mixed micelles of partially purified rat brain phospholipase d activity and its activation by phosphatidylinositol 4,5-bisphosphate. Neurochem. Res. 23, 589–599. doi: 10.1023/a:1022422418388 9566596

[B11] Chapman K. D. Moore T. S. (1993). N-acylphosphatidylethanolamine synthesis in plants: Occurrence, molecular composition, and phospholipid origin. Arch. Biochem. Biophys. 301, 21–33. doi: 10.1006/abbi.1993.1110 8442663

[B12] Chen G. Harwood J. L. Lemieux M. J. Stone S. J. Weselake R. J. (2022). Acyl-CoA:diacylglycerol acyltransferase: Properties, physiological roles, metabolic engineering and intentional control. Prog. Lipid Res. 88, 101181. doi: 10.1016/j.plipres.2022.101181 35820474

[B13] Chopra R. Johnson E. B. Daniels E. McGinn M. Dorn K. M. Esfahanian M.. (2018). Translational genomics using arabidopsis as a model enables the characterization of pennycress genes through forward and reverse genetics. Plant J. 96, 1093–1105. doi: 10.1111/tpj.14147 30394623

[B14] Chopra R. Johnson E. B. Emenecker R. Cahoon E. B. Lyons J. Kliebenstein D. J.. (2020). Identification and stacking of crucial traits required for the domestication of pennycress. Nat. Food 1, 84–91. doi: 10.1038/s43016-019-0007-z

[B15] Cífková E. Hájek R. Lísa M. HolĿapek M. (2016). Hydrophilic interaction liquid chromatography mass spectrometry of (lyso)phosphatidic acids, (lyso)phosphatidylserines and other lipid classes. J. Chromatogr. A. 1439, 65–73. doi: 10.1016/j.chroma.2016.01.064 26858118

[B16] Cocuron J.-C. Casas M. I. Yang F. Grotewold E. Alonso A. P. (2019). Beyond the wall: High-throughput quantification of plant soluble and cell-wall bound phenolics by liquid chromatography tandem mass spectrometry. J. Chromatogr. A. 1589, 93–104. doi: 10.1016/j.chroma.2018.12.059 30626504

[B17] Colin L. A. Jaillais Y. (2020). Phospholipids across scales: lipid patterns and plant development. Curr. Opin. Plant Biol. 53, 1–9. doi: 10.1016/j.pbi.2019.08.007 31580918

[B18] Davidson I. W. F. Sumner D. D. Parker J. C. (1982). Chloroform: A review of its metabolism, teratogenic, mutagenic, and carcinogenic potential. Drug Chem. Toxicol. 5, 1–87. doi: 10.3109/01480548209017822 6807664

[B19] Durrett T. P. Benning C. Ohlrogge J. (2008). Plant triacylglycerols as feedstocks for the production of biofuels. Plant J. 54, 593–607. doi: 10.1111/j.1365-313X.2008.03442.x 18476866

[B20] Dyer J. M. Stymne S. Green A. G. Carlsson A. S. (2008). High-value oils from plants. Plant J. 54, 640–655. doi: 10.1111/j.1365-313X.2008.03430.x 18476869

[B21] Fan J. Shonnard D. R. Kalnes T. N. Johnsen P. B. Rao S. (2013). A life cycle assessment of pennycress (Thlaspi arvense l.) -derived jet fuel and diesel. Biomass Bioenergy 55, 87–100. doi: 10.1016/j.biombioe.2012.12.040

[B22] Folch J. Lees M. Sloane Stanley G. H. (1957). A simple method for the isolation and purification of total lipides from animal tissues. J. Biol. Chem. 226, 497–509. doi: 10.1016/S0021-9258(18)64849-5 13428781

[B23] Han X. Gross R. W. (2021). The foundations and development of lipidomics. J. Lipid Res. 63 (2), 100164. doi: 10.1016/j.jlr.2021.100164 34953866 PMC8953652

[B24] Hara A. Radin N. S. (1978). Lipid extraction of tissues with a low-toxicity solvent. Anal. Biochem. 90, 420–426. doi: 10.1016/0003-2697(78)90046-5 727482

[B25] He M. Qin C.-X. Wang X. Ding N.-Z. (2020). Plant unsaturated fatty acids: Biosynthesis and regulation. Front. Plant Sci. 11. doi: 10.3389/fpls.2020.00390 PMC7212373 32425958

[B26] Isaac G. Wilson I. D. Plumb R. S. (2022). Application of hybrid surface technology for improving sensitivity and peak shape of phosphorylated lipids such as phosphatidic acid and phosphatidylserine. J. Chromatogr. A. 1669, 462921. doi: 10.1016/j.chroma.2022.462921 35272103

[B27] Ischebeck T. Krawczyk H. E. Mullen R. T. Dyer J. M. Chapman K. D. (2020). Lipid droplets in plants and algae: Distribution, formation, turnover and function. Semin. Cell Dev. Biol. 108, 82–93. doi: 10.1016/j.semcdb.2020.02.014 32147380

[B28] Jankevics A. Jenkins A. Dunn W. B. Najdekr L. (2021). An improved strategy for analysis of lipid molecules utilising a reversed phase C30 UHPLC column and scheduled MS/MS acquisition. Talanta 229, 122262. doi: 10.1016/j.talanta.2021.122262 33838772

[B29] Jarvis B. A. Romsdahl T. B. McGinn M. G. Nazarenus T. J. Cahoon E. B. Chapman K. D.. (2021). CRISPR/Cas9-induced fad2 and rod1 mutations stacked with fae1 confer high oleic acid seed oil in pennycress (Thlaspi arvense l.). Front. Plant Sci. 12. doi: 10.3389/fpls.2021.652319 PMC8100250 33968108

[B30] Jennings W. Epand R. M. (2020). CDP-diacylglycerol, a critical intermediate in lipid metabolism. Chem. Phys. Lipids 230, 104914. doi: 10.1016/j.chemphyslip.2020.104914 32360136

[B31] Jeppson S. Demski K. Carlsson A. S. Zhu L.-H. Banaś A. Stymne S.. (2019). *Crambe hispanica* subsp. *abyssinica* diacylglycerol acyltransferase specificities towards diacylglycerols and acyl-CoA reveal combinatorial effects that greatly affect enzymatic activity and specificity. Front. Plant Sci. 10. doi: 10.3389/fpls.2019.01442 PMC6863138 31798607

[B32] Johnston C. García Navarrete L. T. Ortiz E. Romsdahl T. B. Guzha A. Chapman K. D.. (2022). Effective mechanisms for improving seed oil production in pennycress (*Thlaspi arvense* l.) highlighted by integration of comparative metabolomics and transcriptomics. Front. Plant Sci. 13. doi: 10.3389/fpls.2022.943585 PMC9330397 35909773

[B33] Kennedy E. P. (1961). Biosynthesis of complex lipids. Fed. Proc. 20, 934–940. Available at: https://archive.org/details/sim_federation-of-american-soc-experimental-biology_1961-12_20_4/page/934/mode/2up. 14455159

[B34] Kerwin J. L. Tuininga A. R. Ericsson L. H. (1994). Identification of molecular species of glycerophospholipids and sphingomyelin using electrospray mass spectrometry. J. Lipid Res. 35, 1102–1114. doi: 10.1016/S0022-2275(20)40106-3 8077849

[B35] Lager I. Jeppson S. Gippert A.-L. Feussner I. Stymne S. Marmon S. (2020). Acyltransferases regulate oil quality in *Camelina sativa* through both acyl donor and acyl acceptor specificities. Front. Plant Sci. 11. doi: 10.3389/fpls.2020.01144 PMC7456936 32922411

[B36] Lager I. Yilmaz J. L. Zhou X.-R. Jasieniecka K. Kazachkov M. Wang P.. (2013). Plant acyl-CoA : Lysophosphatidylcholine acyltransferases (LPCATs) have different specificities in their forward and reverse reactions. J. Biol. Chem. 288, 36902–36914. doi: 10.1074/jbc.M113.521815 24189065 PMC3873549

[B37] Lands W. E. M. (1965). Lipid metabolism. Annu. Rev. Biochem. 34, 313–346. doi: 10.1146/annurev.bi.34.070165.001525 14321173

[B38] Li-Beisson Y. Shorrosh B. Beisson F. Andersson M. X. Arondel V. Bates P. D.. (2010). Acyl-lipid metabolism. Arab. Book 8, e0133. doi: 10.1199/tab.0133 PMC3244904 22303259

[B39] Löfgren L. Forsberg G.-B. Ståhlman M. (2016). The BUME method: a new rapid and simple chloroform-free method for total lipid extraction of animal tissue. Sci. Rep. 6, 27688. doi: 10.1038/srep27688 27282822 PMC4901324

[B40] Lu C. Xin Z. Ren Z. Miquel M. Browse J. (2009). An enzyme regulating triacylglycerol composition is encoded by the ROD1 gene of arabidopsis. Proc. Natl. Acad. Sci. 106, 18837–18842. doi: 10.1073/pnas.0908848106 19833868 PMC2774007

[B41] Matyash V. Liebisch G. Kurzchalia T. V. Shevchenko A. Schwudke D. (2008). Lipid extraction by methyl-tert-butyl ether for high-throughput lipidomics. J. Lipid Res. 49, 1137–1146. doi: 10.1194/jlr.D700041-JLR200 18281723 PMC2311442

[B42] McGinn M. Phippen W. B. Chopra R. Bansal S. Jarvis B. A. Phippen M. E.. (2019). Molecular tools enabling pennycress (*Thlaspi arvense*) as a model plant and oilseed cash cover crop. Plant Biotechnol. J. 17, 776–788. doi: 10.1111/pbi.13014 30230695 PMC6419581

[B43] Moser B. R. (2012). Biodiesel from alternative oilseed feedstocks: camelina and field pennycress. Biofuels 3, 193–209. doi: 10.4155/bfs.12.6

[B44] Moser B. R. Shah S. N. Winkler-Moser J. K. Vaughn S. F. Evangelista R. L. (2009). Composition and physical properties of cress (Lepidium sativum l.) and field pennycress (Thlaspi arvense l.) oils. Ind. Crops Prod. 30, 199–205. doi: 10.1016/j.indcrop.2009.03.007

[B45] Munnik T. (2001). Phosphatidic acid: an emerging plant lipid second messenger. Trends Plant Sci. 6, 227–233. doi: 10.1016/S1360-1385(01)01918-5 11335176

[B46] Nakamura Y. (2021). Headgroup biosynthesis of phosphatidylcholine and phosphatidylethanolamine in seed plants. Prog. Lipid Res. 82, 101091. doi: 10.1016/j.plipres.2021.101091 33503494

[B47] Narváez-Rivas M. Zhang Q. (2016). Comprehensive untargeted lipidomic analysis using core–shell C30 particle column and high field orbitrap mass spectrometer. J. Chromatogr. A. 1440, 123–134. doi: 10.1016/j.chroma.2016.02.054 26928874 PMC4792668

[B48] Ogiso H. Suzuki T. Taguchi R. (2008). Development of a reverse-phase liquid chromatography electrospray ionization mass spectrometry method for lipidomics, improving detection of phosphatidic acid and phosphatidylserine. Anal. Biochem. 375, 124–131. doi: 10.1016/j.ab.2007.12.027 18206977

[B49] Ohlrogge J. Chapman K. (2011). The seeds of green energy: Expanding the contribution of plant oils as biofuels. Biochem. 33, 34–38. doi: 10.1042/BIO03302034

[B50] Okazaki Y. Saito K. (2014). Roles of lipids as signaling molecules and mitigators during stress response in plants. Plant J. 79, 584–596. doi: 10.1111/tpj.12556 24844563

[B51] Okusa K. Iwasaki Y. Kuroda I. Miwa S. Ohira M. Nagai T.. (2014). Effect of pressure on the selectivity of polymeric C18 and C30 stationary phases in reversed-phase liquid chromatography. increased separation of isomeric fatty acid methyl esters, triacylglycerols, and tocopherols at high pressure. J. Chromatogr. A. 1339, 86–95. doi: 10.1016/j.chroma.2014.02.077 24666940

[B52] Pang Z. Chong J. Zhou G. de Lima Morais D. A. Chang L. Barrette M.. (2021). MetaboAnalyst 5.0: narrowing the gap between raw spectra and functional insights. Nucleic Acids Res. 49, W388–W396. doi: 10.1093/nar/gkab382 34019663 PMC8265181

[B53] Pham T. H. Zaeem M. Fillier T. A. Nadeem M. Vidal N. P. Manful C.. (2019). Targeting modified lipids during routine lipidomics analysis using HILIC and C30 reverse phase liquid chromatography coupled to mass spectrometry. Sci. Rep. 9, 5048. doi: 10.1038/s41598-019-41556-9 30911033 PMC6433904

[B54] Pollard M. Shachar-Hill Y. (2022). Kinetic complexities of triacylglycerol accumulation in developing embryos from camelina sativa provide evidence for multiple biosynthetic systems. J. Biol. Chem. 298, 101396. doi: 10.1016/j.jbc.2021.101396 34774796 PMC8715117

[B55] Rampler E. Criscuolo A. Zeller M. El Abiead Y. Schoeny H. Hermann G.. (2018). A novel lipidomics workflow for improved human plasma identification and quantification using RPLC-MSn methods and isotope dilution strategies. Anal. Chem. 90, 6494–6501. doi: 10.1021/acs.analchem.7b05382 29708737

[B56] Reszczyńska E. Hanaka A. (2020). Lipids composition in plant membranes. Cell Biochem. Biophys. 78, 401–414. doi: 10.1007/s12013-020-00947-w 33034870 PMC7567678

[B57] Romsdahl T. B. Kambhampati S. Koley S. Yadav U. P. Alonso A. P. Allen D. K.. (2021). Analyzing mass spectrometry imaging data of 13C-labeled phospholipids in camelina sativa and thlaspi arvense (Pennycress) embryos. Metabolites 11, 148. doi: 10.3390/metabo11030148 33806402 PMC7999836

[B58] Routaboul J.-M. Benning C. Bechtold N. Caboche M. Lepiniec L. (1999). The TAG1 locus of arabidopsis encodes for a diacylglycerol acyltransferase. Plant Physiol. Biochem. 37, 831–840. doi: 10.1016/S0981-9428(99)00115-1 10580283

[B59] Rustam Y. H. Reid G. E. (2018). Analytical challenges and recent advances in mass spectrometry based lipidomics. Anal. Chem. 90, 374–397. doi: 10.1021/acs.analchem.7b04836 29166560

[B60] Sedbrook J. C. Durrett T. P. (2020). Pennycress, carbon wise: labeling experiments reveal how pennycress seeds efficiently incorporate carbon into biomass. J. Exp. Bot. 71, 2842–2846. doi: 10.1093/jxb/eraa136 32472690 PMC7260712

[B61] Sedbrook J. C. Phippen W. B. Marks M. D. (2014). New approaches to facilitate rapid domestication of a wild plant to an oilseed crop: Example pennycress (Thlaspi arvense l.). Plant Sci. 227, 122–132. doi: 10.1016/j.plantsci.2014.07.008 25219314

[B62] Shiva S. Enninful R. Roth M. R. Tamura P. Jagadish K. Welti R. (2018). An efficient modified method for plant leaf lipid extraction results in improved recovery of phosphatidic acid. Plant Methods 14, 14. doi: 10.1186/s13007-018-0282-y 29449874 PMC5812192

[B63] Shockey J. Regmi A. Cotton K. Adhikari N. Browse J. Bates P. D. (2016). Identification of arabidopsis *GPAT9* (At5g60620) as an essential gene involved in triacylglycerol biosynthesis. Plant Physiol. 170, 163–179. doi: 10.1104/pp.15.01563 26586834 PMC4704598

[B64] Taguchi R. Houjou T. Nakanishi H. Yamazaki T. Ishida M. Imagawa M.. (2005). Focused lipidomics by tandem mass spectrometry. J. Chromatogr. B. 823, 26–36. doi: 10.1016/j.jchromb.2005.06.005 15990370

[B65] Tsogtbaatar E. Cocuron J.-C. Alonso A. P. (2020). Non-conventional pathways enable pennycress (Thlaspi arvense l.) embryos to achieve high efficiency of oil biosynthesis. J. Exp. Bot. 71, 3037–3051. doi: 10.1093/jxb/eraa060 32006014 PMC7260723

[B66] Tsogtbaatar E. Cocuron J.-C. Sonera M. C. Alonso A. P. (2015). Metabolite fingerprinting of pennycress (*Thlaspi arvense* l.) embryos to assess active pathways during oil synthesis. J. Exp. Bot. 66, 4267–4277. doi: 10.1093/jxb/erv020 25711705 PMC4493779

[B67] Welti R. Li W. Li M. Sang Y. Biesiada H. Zhou H.-E.. (2002). Profiling membrane lipids in plant stress responses. J. Biol. Chem. 277, 31994–32002. doi: 10.1074/jbc.M205375200 12077151

[B68] Welti R. Wang X. Williams T. D. (2003). Electrospray ionization tandem mass spectrometry scan modes for plant chloroplast lipids. Anal. Biochem. 314, 149–152. doi: 10.1016/S0003-2697(02)00623-1 12633615

[B69] Yang W. Wang G. Li J. Bates P. D. Wang X. Allen D. K. (2017). Phospholipase dζ enhances diacylglycerol flux into triacylglycerol. Plant Physiol. 174, 110–123. doi: 10.1104/pp.17.00026 28325849 PMC5411150

