# Supplementary material for: A lipidomics platform to analyze the fatty acid compositions of non-polar and polar lipid molecular species from plant tissues: Examples from developing seeds and seedlings of pennycress (Thlaspi arvense)
Source: Front Plant Sci. 2022 Nov 9;13:1038161. doi: 10.3389/fpls.2022.1038161 (PMC9682148; doi:10.3389/fpls.2022.1038161)
Supplement: Supplementary file 1 [file DataSheet_1.docx]

Supplementary Material


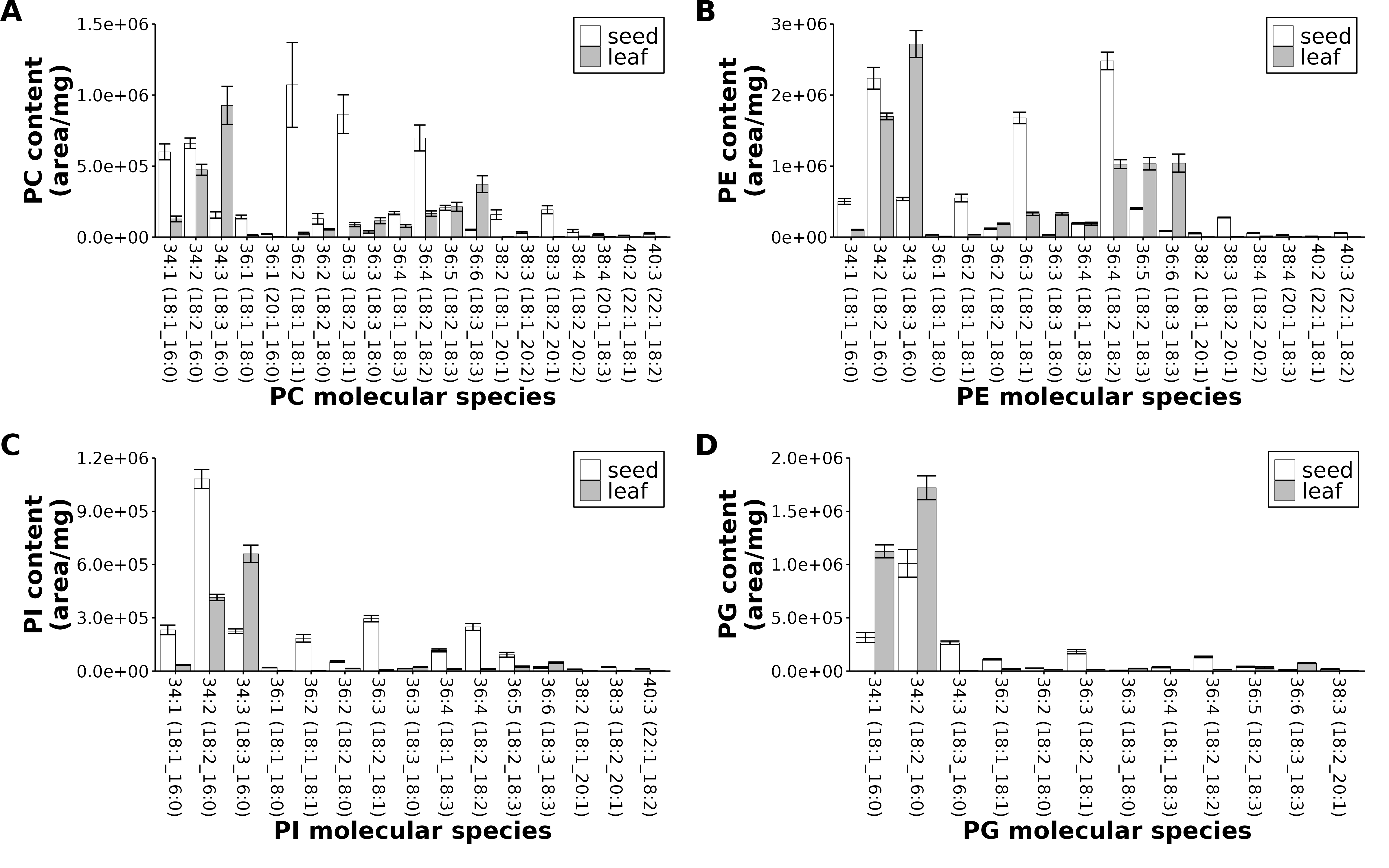


**Supplemental Figure 1. Phospholipids of pennycress seed and leaf tissues.** Comparison of the major molecular species of phosphatidylcholine (PC) (**A**), phosphatidylethanolamine (PE) (**B**), phosphatidylinositol (PI) (**C**), and phosphatidylglycerol (PG) (**D**) from seed and leaf tissues. Values represent the average LC peak area for each molecular species, normalized by tissue weight. (n = 4, ± S.D.)


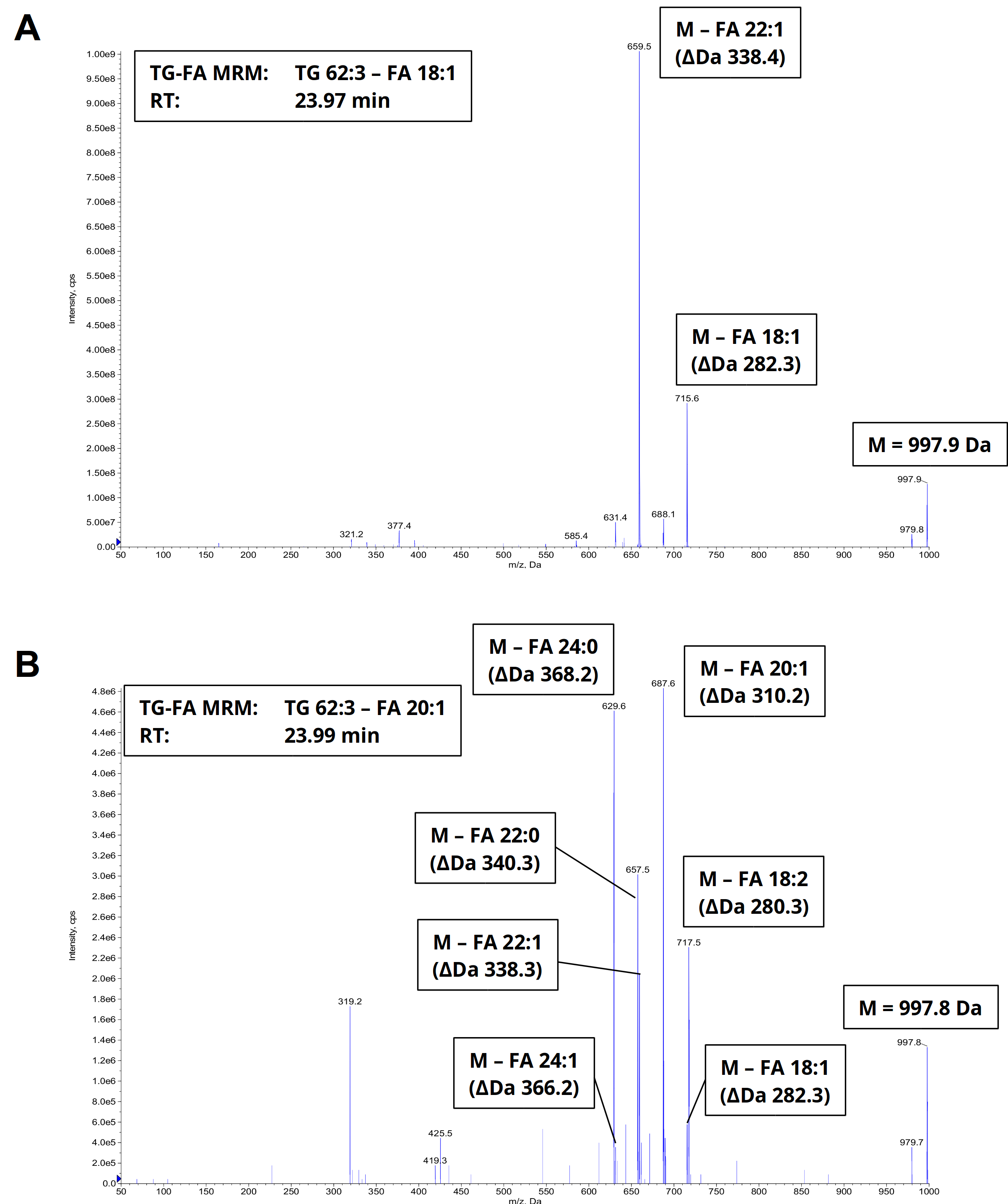


**Supplemental Figure 2. TG-FA IDA confirmation for TG 62:3.** The EPI fragmentation of the MRM for TG 62:3 – FA 18:1 (**A**) shows fragmentations of FA 22:1 at *m/z* 659.5 and FA 18:1 at *m/z* 715.6, which would suggest a final FA combination of 18:1_22:1_22:1. For TG 62:3 – FA 20:1 (**B**), many more fragment ion peaks are observed suggesting multiple possible FA combinations (e.g. 20:1_18:2_24:0, 20:1_18:1_24:1, 20:1_22:1_20:1).


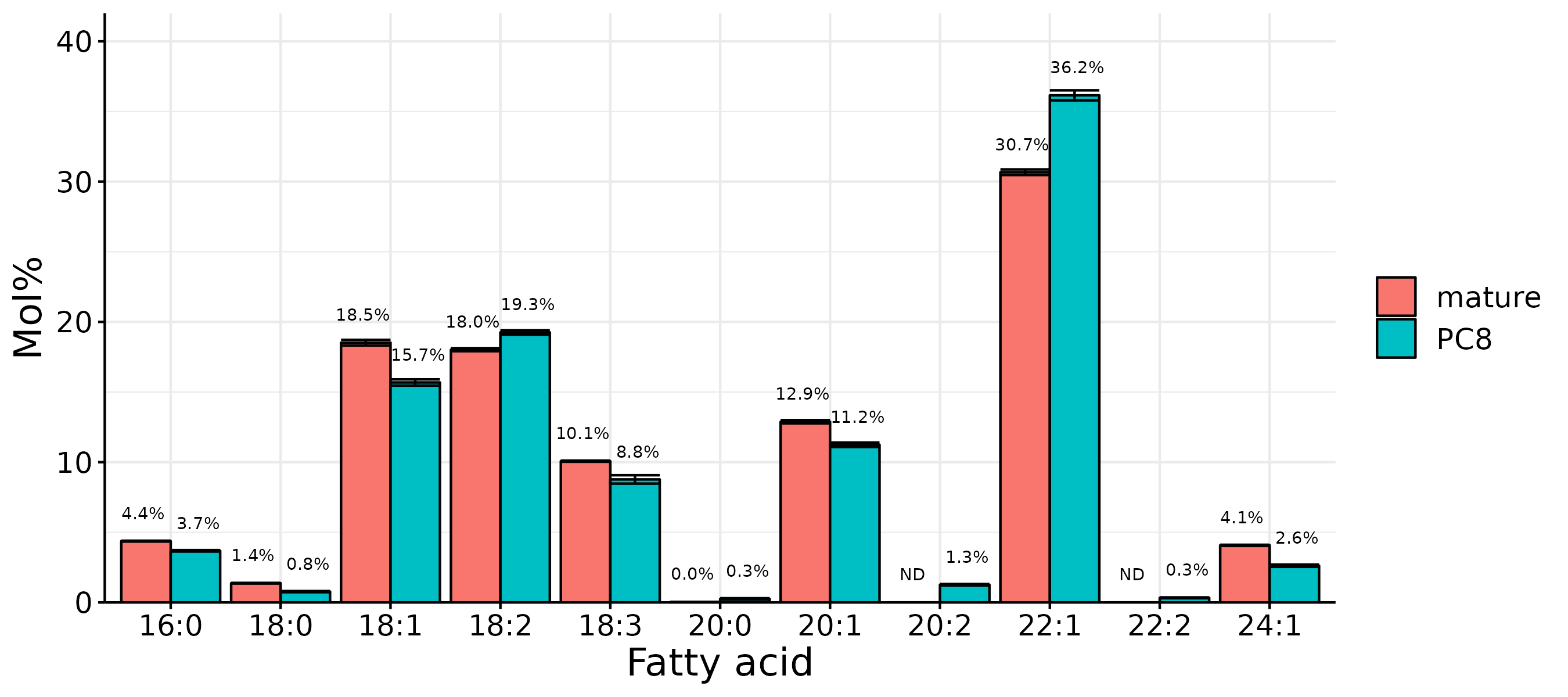


**Supplemental Figure 3. Comparison of TG FA mol% relative to GC-MS FA mol% of pennycress seeds.** The FA mol% was calculated from the TG content measured from mature pennycress seeds through the non-polar method (mature, pink) and compared to GC-MS analysis of FAMEs of pennycress seeds of the same accession (PC8, blue). (n = 4, ± S.D.; ND = not detected)


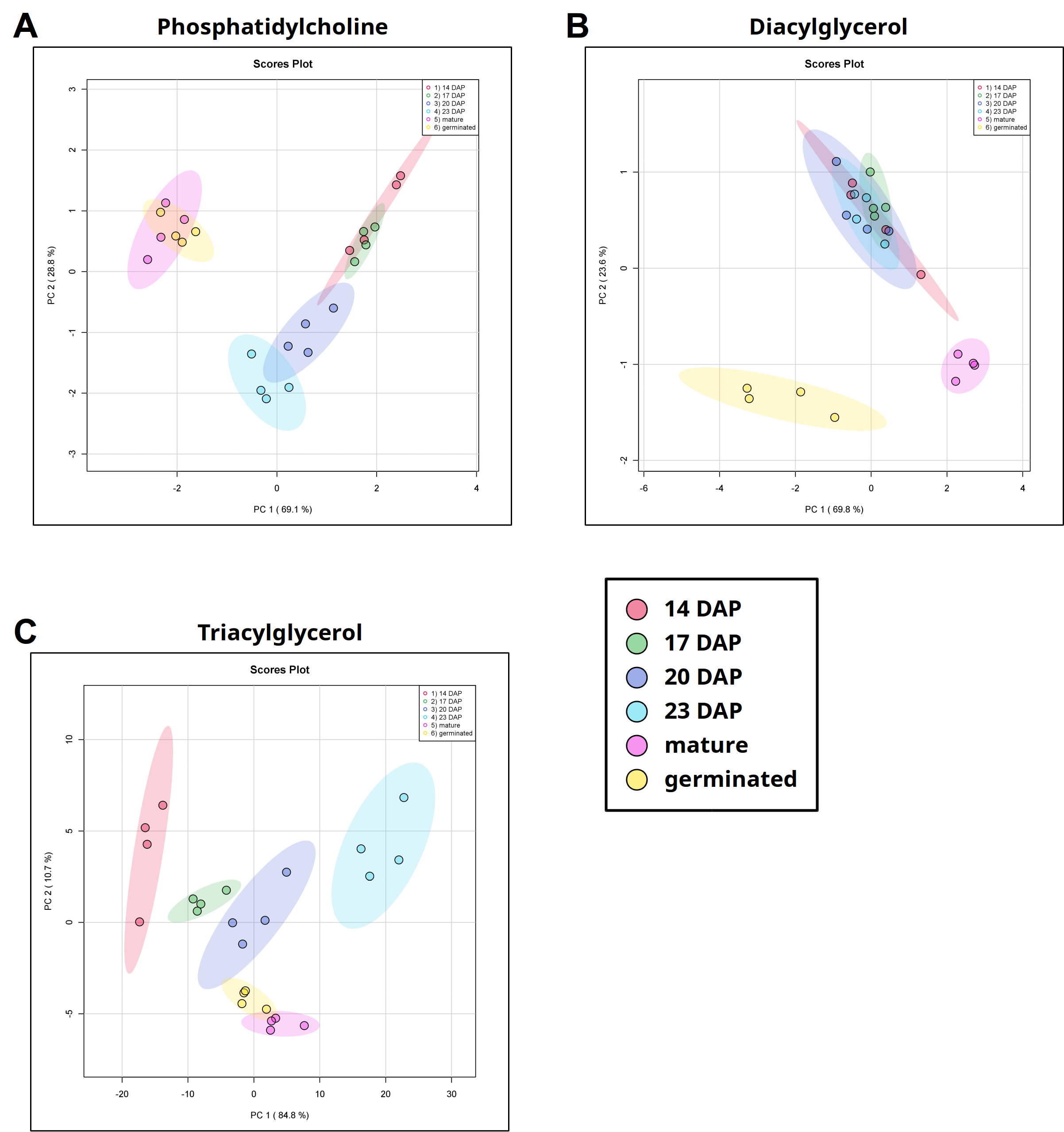


**Supplemental Figure 4. Principal component analysis (PCA) of PC, DG, and TG from developing pennycress seeds.** PCA plots of PC (**A**), DG (**B**), and TG (**C**) from developing pennycress seeds at 14, 17, 20, and 23 DAP, as well as mature and germinated seeds. PC1 and 2 of PC collectively represent 97.9% of the total variance; PC1 and 2 of DG collectively represent 93.4% of the total variance; and PC1 and 2 of TG collectively represent 95.5% of the total variance. PCA was done using MetaboAnalyst; shaded regions represent 95% confidence intervals.


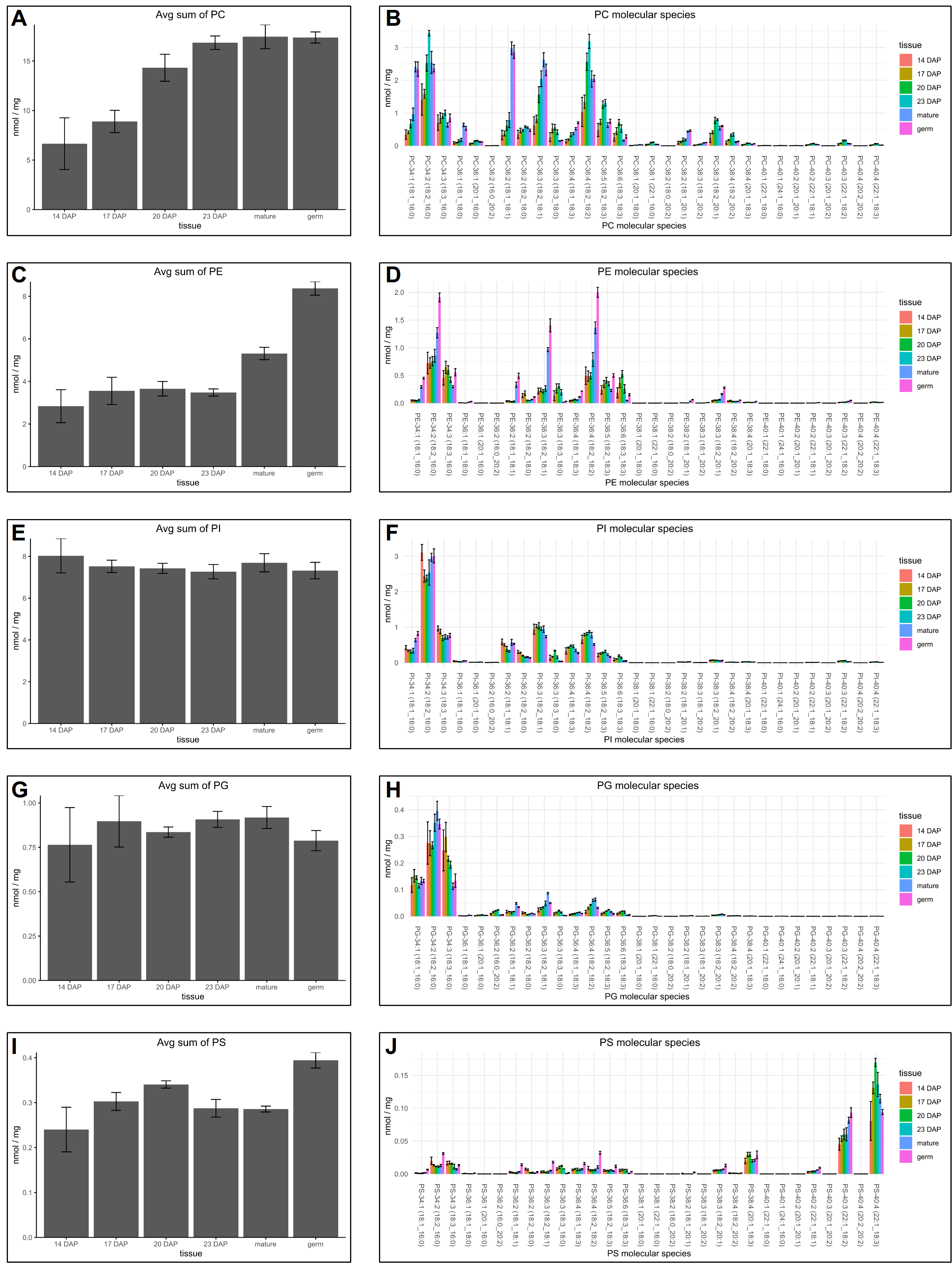


**Supplemental Figure 5. Phospholipid content and composition from developing pennycress seeds.** Total sum and individual molecular species of PC (**A**, **B**), PE (**C**, **D**), PI (**E**, **F**), PG (**G**, **H**), and PS (**I**, **J**) from developing pennycress seeds at 14, 17, 20, 23, DAP and mature and germinated seeds. (n = 4, ± S.D.)


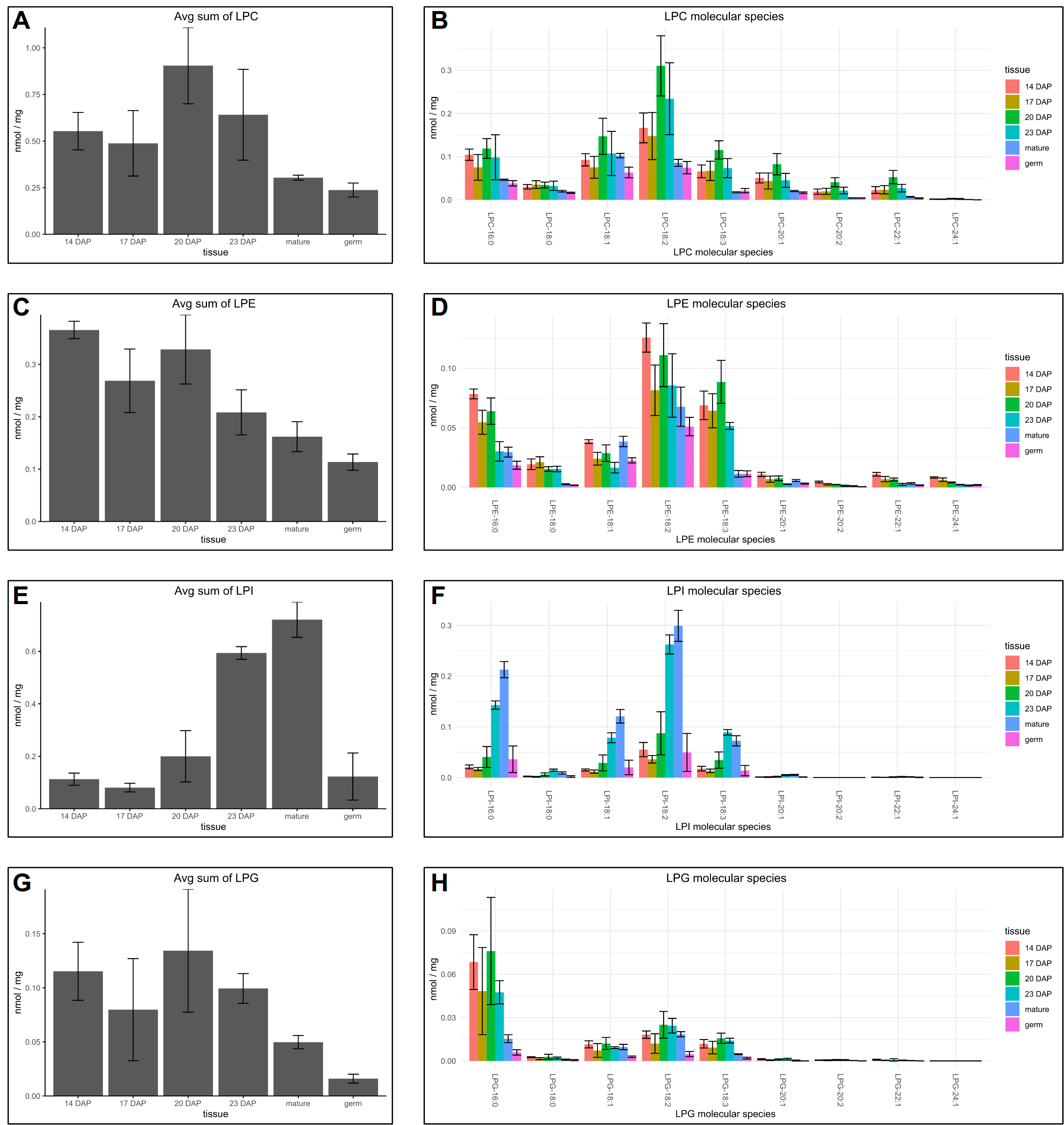


**Supplemental Figure 6. Lysophospholipid content and composition from developing pennycress seeds.** Total sum and individual molecular species of LPC (**A**, **B**), LPE (**C**, **D**), LPI (**E**, **F**), and LPG (**G**, **H**) from developing pennycress seeds at 14, 17, 20, 23, DAP and mature and germinated seeds. (n = 4, ± S.D.)


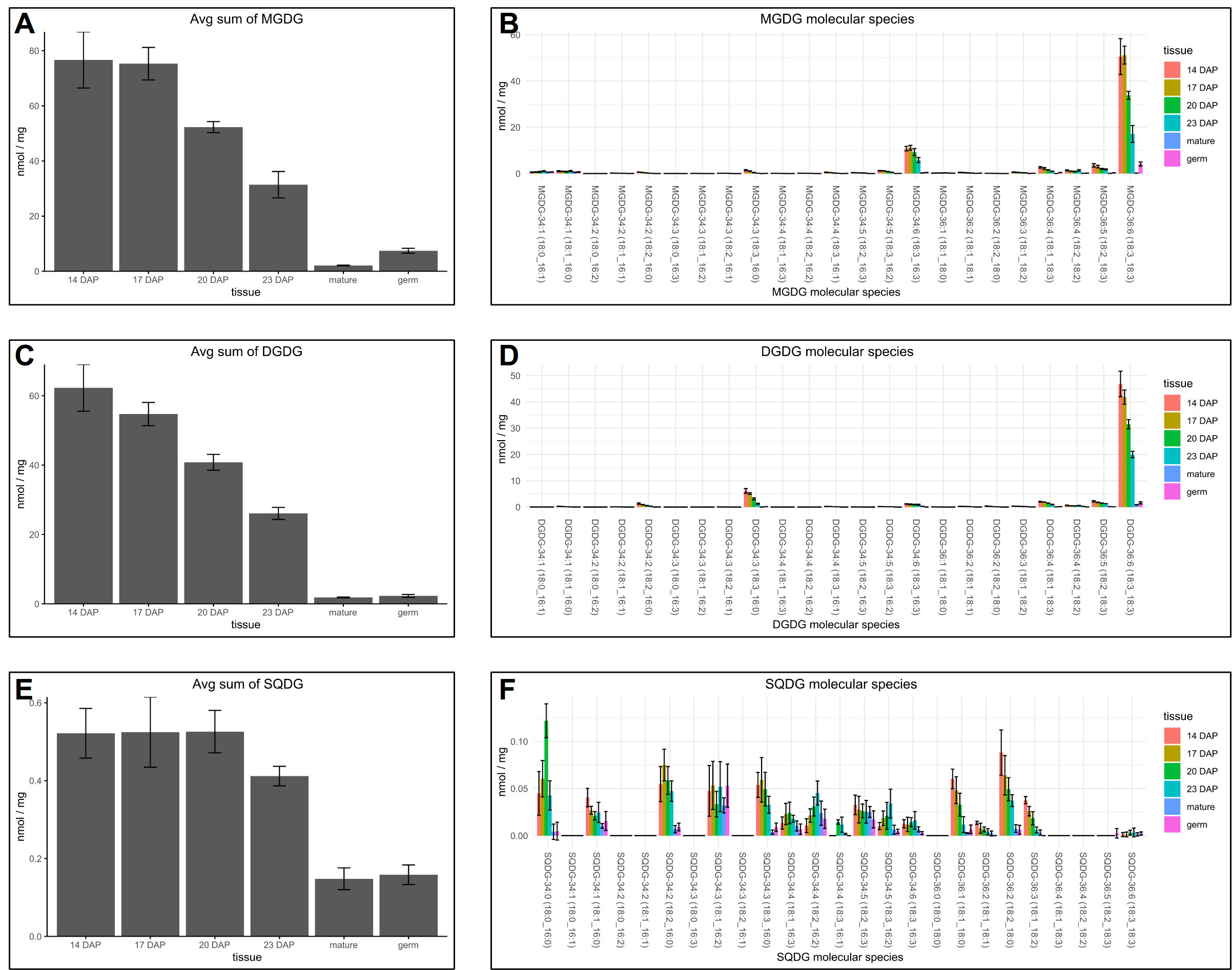


**Supplemental Figure 7. Galactolipid and sulfoquinovosyl diacylglycerol content and composition from developing pennycress seeds.** Total sum and individual molecular species of MGDG (**A**, **B**), DGDG (**C**, **D**), and SQDG (**E**, **F**) from developing pennycress seeds at 14, 17, 20, 23, DAP and mature and germinated seeds. (n = 4, ± S.D.)
