# Supplementary material for: A lipidomics platform to analyze the fatty acid compositions of non-polar and polar lipid molecular species from plant tissues: Examples from developing seeds and seedlings of pennycress (Thlaspi arvense)
Source: Front Plant Sci. 2022 Nov 9;13:1038161. doi: 10.3389/fpls.2022.1038161 (PMC9682148; doi:10.3389/fpls.2022.1038161)
Supplement: Supplementary file 2 [file DataSheet_2.docx]

# Polar lipidomics data processing script

# Order of components in R script + stds

# PC stds: "PC-31:1-d5 (14:1_17:0)", "PC-35:1-d5 (17:0_18:1)", "PC-33:1-d5 (17:0_16:1)", "PC-37:3-d5 (17:0_20:3)", "PC-39:4-d5 (17:0_22:4)"

# PE stds: "PE-31:1-d5 (17:0_14:1)", "PE-35:1-d5 (17:0_18:1)", "PE-33:1-d5 (17:0_16:1)", "PE-37:3-d5 (17:0_20:3)", "PE-39:4-d5 (17:0_22:4)"

# DGDG stds: "DGDG-36:0 (18:0_18:0)_2"

# LPC stds: "LPC-15:0-d5", "LPC-19:0-d5", "LPC-17:0-d5"

# LPE stds: "LPE-15:0-d5", "LPE-19:0-d5", "LPE-17:0-d5"

# LPG stds: "LPG-15:0-d5", "LPG-19:0-d5", "LPG-17:0-d5"

# LPI stds: "LPI-15:0-d5", "LPI-19:0-d5", "LPI-17:0-d5"

# LPS stds: "LPS-15:0-d5", "LPS-19:0-d5", "LPS-17:0-d5"

# MGDG stds: "MGDG-34:0 (16:0_18:0)"

# PG stds: "PG-31:1-d5 (17:0_14:1)", "PG-35:1-d5 (17:0_18:1)", "PG-33:1-d5 (17:0_16:1)", "PG-37:3-d5 (17:0_20:3)", "PG-39:4-d5 (17:0_22:4)"

# PI stds: "PI-31:1-d5 (17:0_14:1)", "PI-35:1-d5 (17:0_18:1)", "PI-33:1-d5 (17:0_16:1)", "PI-37:3-d5 (17:0_20:3)", "PI-39:4-d5 (17:0_22:4)"

# PS stds: "PS-31:1-d5 (17:0_14:1)", "PS-35:1-d5 (17:0_18:1)", "PS-33:1-d5 (17:0_16:1)", "PS-37:3-d5 (17:0_20:3)", "PS-39:4-d5 (17:0_22:4)"

# SQDG stds: "MGDG-34:0 (16:0_18:0)"

# column headings in data file

# "sample_id" "sample_no" " "tissue" "component_name" "component_group" "mrm" "area" "height" "rt" "baseline" "tissue_wt"

# setting working directory containing data

setwd("PATH TO YOUR DATA FILE")

# loading libraries

library(tidyverse)

library(data.table)

# setting seed_polars as df

seed_polars <- read.delim(file = "FILENAME OF YOUR DATA FILE")

# separating classes into individual dfs

seed_polars_PC <- filter(seed_polars, component_group == "PC")

seed_polars_PE <- filter(seed_polars, component_group == "PE")

seed_polars_DGDG <- filter(seed_polars, component_group == "DGDG")

seed_polars_LPC <- filter(seed_polars, component_group == "LPC")

seed_polars_LPE <- filter(seed_polars, component_group == "LPE")

seed_polars_LPG <- filter(seed_polars, component_group == "LPG")

seed_polars_LPI <- filter(seed_polars, component_group == "LPI")

seed_polars_LPS <- filter(seed_polars, component_group == "LPS")

seed_polars_MGDG <- filter(seed_polars, component_group == "MGDG")

seed_polars_PG <- filter(seed_polars, component_group == "PG")

seed_polars_PI <- filter(seed_polars, component_group == "PI")

seed_polars_PS <- filter(seed_polars, component_group == "PS")

seed_polars_SQDG <- filter(seed_polars, component_group == "SQDG")

# writing class files into '\t' delimited .txt files

write.table(seed_polars_PC, file = "seed_polars_PC.txt", quote = FALSE, row.names = FALSE, sep = "\t")

write.table(seed_polars_PE, file = "seed_polars_PE.txt", quote = FALSE, row.names = FALSE, sep = "\t")

write.table(seed_polars_DGDG, file = "seed_polars_DGDG.txt", quote = FALSE, row.names = FALSE, sep = "\t")

write.table(seed_polars_LPC, file = "seed_polars_LPC.txt", quote = FALSE, row.names = FALSE, sep = "\t")

write.table(seed_polars_LPE, file = "seed_polars_LPE.txt", quote = FALSE, row.names = FALSE, sep = "\t")

write.table(seed_polars_LPG, file = "seed_polars_LPG.txt", quote = FALSE, row.names = FALSE, sep = "\t")

write.table(seed_polars_LPI, file = "seed_polars_LPI.txt", quote = FALSE, row.names = FALSE, sep = "\t")

write.table(seed_polars_LPS, file = "seed_polars_LPS.txt", quote = FALSE, row.names = FALSE, sep = "\t")

write.table(seed_polars_MGDG, file = "seed_polars_MGDG.txt", quote = FALSE, row.names = FALSE, sep = "\t")

write.table(seed_polars_PG, file = "seed_polars_PG.txt", quote = FALSE, row.names = FALSE, sep = "\t")

write.table(seed_polars_PI, file = "seed_polars_PI.txt", quote = FALSE, row.names = FALSE, sep = "\t")

write.table(seed_polars_PS, file = "seed_polars_PS.txt", quote = FALSE, row.names = FALSE, sep = "\t")

write.table(seed_polars_SQDG, file = "seed_polars_SQDG.txt", quote = FALSE, row.names = FALSE, sep = "\t")

# separating molecular species without internal standards

setDT(seed_polars_PC)

seed_polars_PC_species <- seed_polars_PC[!(component_name %in% c("PC-31:1-d5 (14:1_17:0)", "PC-35:1-d5 (17:0_18:1)", "PC-33:1-d5 (17:0_16:1)", "PC-37:3-d5 (17:0_20:3)", "PC-39:4-d5 (17:0_22:4)"))]

setDT(seed_polars_PE)

seed_polars_PE_species <- seed_polars_PE[!(component_name %in% c("PE-31:1-d5 (17:0_14:1)", "PE-35:1-d5 (17:0_18:1)", "PE-33:1-d5 (17:0_16:1)", "PE-37:3-d5 (17:0_20:3)", "PE-39:4-d5 (17:0_22:4)"))]

setDT(seed_polars_DGDG)

seed_polars_DGDG_species <- seed_polars_DGDG[!(component_name %in% c("DGDG-36:0 (18:0_18:0)_2", "DGDG-34:0 (18:0_16:0)", "DGDG-36:0 (18:0_18:0)"))]

setDT(seed_polars_LPC)

seed_polars_LPC_species <- seed_polars_LPC[!(component_name %in% c("LPC-15:0-d5", "LPC-19:0-d5", "LPC-17:0-d5"))]

setDT(seed_polars_LPE)

seed_polars_LPE_species <- seed_polars_LPE[!(component_name %in% c("LPE-15:0-d5", "LPE-19:0-d5", "LPE-17:0-d5"))]

setDT(seed_polars_LPG)

seed_polars_LPG_species <- seed_polars_LPG[!(component_name %in% c("LPG-15:0-d5", "LPG-19:0-d5", "LPG-17:0-d5"))]

setDT(seed_polars_LPI)

seed_polars_LPI_species <- seed_polars_LPI[!(component_name %in% c("LPI-15:0-d5", "LPI-19:0-d5", "LPI-17:0-d5"))]

setDT(seed_polars_LPS)

seed_polars_LPS_species <- seed_polars_LPS[!(component_name %in% c("LPS-15:0-d5", "LPS-19:0-d5", "LPS-17:0-d5"))]

setDT(seed_polars_MGDG)

seed_polars_MGDG_species <- seed_polars_MGDG[!(component_name %in% c("MGDG-34:0 (16:0_18:0)", "MGDG-34:0 (18:0_16:0)", "MGDG-36:0 (18:0_18:0)"))]

setDT(seed_polars_PG)

seed_polars_PG_species <- seed_polars_PG[!(component_name %in% c("PG-31:1-d5 (17:0_14:1)", "PG-35:1-d5 (17:0_18:1)", "PG-33:1-d5 (17:0_16:1)", "PG-37:3-d5 (17:0_20:3)", "PG-39:4-d5 (17:0_22:4)"))]

setDT(seed_polars_PI)

seed_polars_PI_species <- seed_polars_PI[!(component_name %in% c("PI-31:1-d5 (17:0_14:1)", "PI-35:1-d5 (17:0_18:1)", "PI-33:1-d5 (17:0_16:1)", "PI-37:3-d5 (17:0_20:3)", "PI-39:4-d5 (17:0_22:4)"))]

setDT(seed_polars_PS)

seed_polars_PS_species <- seed_polars_PS[!(component_name %in% c("PS-31:1-d5 (17:0_14:1)", "PS-35:1-d5 (17:0_18:1)", "PS-33:1-d5 (17:0_16:1)", "PS-37:3-d5 (17:0_20:3)", "PS-39:4-d5 (17:0_22:4)"))]

setDT(seed_polars_SQDG)

seed_polars_SQDG_species <- seed_polars_SQDG

# separating internal standards

seed_polars_PC_stds <- seed_polars_PC[component_name %in% c("PC-31:1-d5 (14:1_17:0)", "PC-35:1-d5 (17:0_18:1)", "PC-33:1-d5 (17:0_16:1)", "PC-37:3-d5 (17:0_20:3)", "PC-39:4-d5 (17:0_22:4)")]

seed_polars_PE_stds <- seed_polars_PE[component_name %in% c("PE-31:1-d5 (17:0_14:1)", "PE-35:1-d5 (17:0_18:1)", "PE-33:1-d5 (17:0_16:1)", "PE-37:3-d5 (17:0_20:3)", "PE-39:4-d5 (17:0_22:4)")]

seed_polars_DGDG_stds <- seed_polars_DGDG[component_name %in% c("DGDG-36:0 (18:0_18:0)_2", "DGDG-34:0 (18:0_16:0)", "DGDG-36:0 (18:0_18:0)")]

seed_polars_LPC_stds <- seed_polars_LPC[component_name %in% c("LPC-15:0-d5", "LPC-19:0-d5", "LPC-17:0-d5")]

seed_polars_LPE_stds <- seed_polars_LPE[component_name %in% c("LPE-15:0-d5", "LPE-19:0-d5", "LPE-17:0-d5")]

seed_polars_LPG_stds <- seed_polars_LPG[component_name %in% c("LPG-15:0-d5", "LPG-19:0-d5", "LPG-17:0-d5")]

seed_polars_LPI_stds <- seed_polars_LPI[component_name %in% c("LPI-15:0-d5", "LPI-19:0-d5", "LPI-17:0-d5")]

seed_polars_LPS_stds <- seed_polars_LPS[component_name %in% c("LPS-15:0-d5", "LPS-19:0-d5", "LPS-17:0-d5")]

seed_polars_MGDG_stds <- seed_polars_MGDG[component_name %in% c("MGDG-34:0 (16:0_18:0)", "MGDG-34:0 (18:0_16:0)", "MGDG-36:0 (18:0_18:0)")]

seed_polars_PG_stds <- seed_polars_PG[component_name %in% c("PG-31:1-d5 (17:0_14:1)", "PG-35:1-d5 (17:0_18:1)", "PG-33:1-d5 (17:0_16:1)", "PG-37:3-d5 (17:0_20:3)", "PG-39:4-d5 (17:0_22:4)")]

seed_polars_PI_stds <- seed_polars_PI[component_name %in% c("PI-31:1-d5 (17:0_14:1)", "PI-35:1-d5 (17:0_18:1)", "PI-33:1-d5 (17:0_16:1)", "PI-37:3-d5 (17:0_20:3)", "PI-39:4-d5 (17:0_22:4)")]

seed_polars_PS_stds <- seed_polars_PS[component_name %in% c("PS-31:1-d5 (17:0_14:1)", "PS-35:1-d5 (17:0_18:1)", "PS-33:1-d5 (17:0_16:1)", "PS-37:3-d5 (17:0_20:3)", "PS-39:4-d5 (17:0_22:4)")]

# normalizing against internal standards and tissue weight

seed_polars_PC_stds_IS <- filter(seed_polars_PC_stds, component_name == "PC-35:1-d5 (17:0_18:1)")

setDT(seed_polars_PC_stds_IS); setDT(seed_polars_PC_species)

seed_polars_PC_species[seed_polars_PC_stds_IS, nmol_mg := (3.85 * area) * (1 / i.area) * (1 / tissue_wt), on = "sample_no"]

seed_polars_PE_stds_IS <- filter(seed_polars_PE_stds, component_name == "PE-35:1-d5 (17:0_18:1)")

setDT(seed_polars_PE_stds_IS); setDT(seed_polars_PE_species)

seed_polars_PE_species[seed_polars_PE_stds_IS, nmol_mg := (2.036 * area) * (1 / i.area) * (1 / tissue_wt), on = "sample_no"]

seed_polars_DGDG_stds_IS <- filter(seed_polars_DGDG_stds, component_name == "DGDG-36:0 (18:0_18:0)_2")

setDT(seed_polars_DGDG_stds_IS); setDT(seed_polars_DGDG_species)

seed_polars_DGDG_species[seed_polars_DGDG_stds_IS, nmol_mg := (200 * area) * (1 / i.area) * (1 / tissue_wt), on = "sample_no"]

seed_polars_LPC_stds_IS <- filter(seed_polars_LPC_stds, component_name == "LPC-17:0-d5")

setDT(seed_polars_LPC_stds_IS); setDT(seed_polars_LPC_species)

seed_polars_LPC_species[seed_polars_LPC_stds_IS, nmol_mg := (1.944 * area) * (1 / i.area) * (1 / tissue_wt), on = "sample_no"]

seed_polars_LPE_stds_IS <- filter(seed_polars_LPE_stds, component_name == "LPE-17:0-d5")

setDT(seed_polars_LPE_stds_IS); setDT(seed_polars_LPE_species)

seed_polars_LPE_species[seed_polars_LPE_stds_IS, nmol_mg := (2.118 * area) * (1 / i.area) * (1 / tissue_wt), on = "sample_no"]

seed_polars_LPG_stds_IS <- filter(seed_polars_LPG_stds, component_name == "LPG-17:0-d5")

setDT(seed_polars_LPG_stds_IS); setDT(seed_polars_LPG_species)

seed_polars_LPG_species[seed_polars_LPG_stds_IS, nmol_mg := (1.904 * area) * (1 / i.area) * (1 / tissue_wt), on = "sample_no"]

seed_polars_LPI_stds_IS <- filter(seed_polars_LPI_stds, component_name == "LPI-17:0-d5")

setDT(seed_polars_LPI_stds_IS); setDT(seed_polars_LPI_species)

seed_polars_LPI_species[seed_polars_LPI_stds_IS, nmol_mg := (1.644 * area) * (1 / i.area) * (1 / tissue_wt), on = "sample_no"]

seed_polars_LPS_stds_IS <- filter(seed_polars_LPS_stds, component_name == "LPS-17:0-d5")

setDT(seed_polars_LPS_stds_IS); setDT(seed_polars_LPS_species)

seed_polars_LPS_species[seed_polars_LPS_stds_IS, nmol_mg := (1.858 * area) * (1 / i.area) * (1 / tissue_wt), on = "sample_no"]

seed_polars_MGDG_stds_IS <- filter(seed_polars_MGDG_stds, component_name == "MGDG-34:0 (16:0_18:0)")

setDT(seed_polars_MGDG_stds_IS); setDT(seed_polars_MGDG_species)

seed_polars_MGDG_species[seed_polars_MGDG_stds_IS, nmol_mg := (200 * area) * (1 / i.area) * (1 / tissue_wt), on = "sample_no"]

seed_polars_PG_stds_IS <- filter(seed_polars_PG_stds, component_name == "PG-35:1-d5 (17:0_18:1)")

setDT(seed_polars_PG_stds_IS); setDT(seed_polars_PG_species)

seed_polars_PG_species[seed_polars_PG_stds_IS, nmol_mg := (1.898 * area) * (1 / i.area) * (1 / tissue_wt), on = "sample_no"]

seed_polars_PI_stds_IS <- filter(seed_polars_PI_stds, component_name == "PI-35:1-d5 (17:0_18:1)")

setDT(seed_polars_PI_stds_IS); setDT(seed_polars_PI_species)

seed_polars_PI_species[seed_polars_PI_stds_IS, nmol_mg := (1.718 * area) * (1 / i.area) * (1 / tissue_wt), on = "sample_no"]

seed_polars_PS_stds_IS <- filter(seed_polars_PS_stds, component_name == "PS-35:1-d5 (17:0_18:1)")

setDT(seed_polars_PS_stds_IS); setDT(seed_polars_PS_species)

seed_polars_PS_species[seed_polars_PS_stds_IS, nmol_mg := (1.868 * area) * (1 / i.area) * (1 / tissue_wt), on = "sample_no"]

seed_polars_SQDG_stds_IS <- filter(seed_polars_MGDG_stds, component_name == "MGDG-34:0 (16:0_18:0)")

setDT(seed_polars_SQDG_stds_IS); setDT(seed_polars_SQDG_species)

seed_polars_SQDG_species[seed_polars_SQDG_stds_IS, nmol_mg := (200 * area) * (1 / i.area) * (1 / tissue_wt), on = "sample_no"]

# summing molecular species

seed_polars_PC_sum <- seed_polars_PC_species[, .(sum_nmol_mg = sum(nmol_mg)), by = .(tissue, sample_no)]

seed_polars_PE_sum <- seed_polars_PE_species[, .(sum_nmol_mg = sum(nmol_mg)), by = .(tissue, sample_no)]

seed_polars_DGDG_sum <- seed_polars_DGDG_species[, .(sum_nmol_mg = sum(nmol_mg)), by = .(tissue, sample_no)]

seed_polars_LPC_sum <- seed_polars_LPC_species[, .(sum_nmol_mg = sum(nmol_mg)), by = .(tissue, sample_no)]

seed_polars_LPE_sum <- seed_polars_LPE_species[, .(sum_nmol_mg = sum(nmol_mg)), by = .(tissue, sample_no)]

seed_polars_LPG_sum <- seed_polars_LPG_species[, .(sum_nmol_mg = sum(nmol_mg)), by = .(tissue, sample_no)]

seed_polars_LPI_sum <- seed_polars_LPI_species[, .(sum_nmol_mg = sum(nmol_mg)), by = .(tissue, sample_no)]

seed_polars_LPS_sum <- seed_polars_LPS_species[, .(sum_nmol_mg = sum(nmol_mg)), by = .(tissue, sample_no)]

seed_polars_MGDG_sum <- seed_polars_MGDG_species[, .(sum_nmol_mg = sum(nmol_mg)), by = .(tissue, sample_no)]

seed_polars_PG_sum <- seed_polars_PG_species[, .(sum_nmol_mg = sum(nmol_mg)), by = .(tissue, sample_no)]

seed_polars_PI_sum <- seed_polars_PI_species[, .(sum_nmol_mg = sum(nmol_mg)), by = .(tissue, sample_no)]

seed_polars_PS_sum <- seed_polars_PS_species[, .(sum_nmol_mg = sum(nmol_mg)), by = .(tissue, sample_no)]

seed_polars_SQDG_sum <- seed_polars_SQDG_species[, .(sum_nmol_mg = sum(nmol_mg)), by = .(tissue, sample_no)]

# calculating average and standard deviation for summed and individual molecular species

setDT(seed_polars_PC_sum)

seed_polars_PC_sum_avgSD <- seed_polars_PC_sum[, .(avg_nmol_mg = mean(sum_nmol_mg), SD_nmol_mg = sd(sum_nmol_mg)), by = tissue]

seed_polars_PC_species_avgSD <- seed_polars_PC_species[, .(avg_nmol_mg = mean(nmol_mg), SD_nmol_mg = sd(nmol_mg)), by = .(tissue, component_name)]

setDT(seed_polars_PE_sum)

seed_polars_PE_sum_avgSD <- seed_polars_PE_sum[, .(avg_nmol_mg = mean(sum_nmol_mg), SD_nmol_mg = sd(sum_nmol_mg)), by = tissue]

seed_polars_PE_species_avgSD <- seed_polars_PE_species[, .(avg_nmol_mg = mean(nmol_mg), SD_nmol_mg = sd(nmol_mg)), by = .(tissue, component_name)]

setDT(seed_polars_DGDG_sum)

seed_polars_DGDG_sum_avgSD <- seed_polars_DGDG_sum[, .(avg_nmol_mg = mean(sum_nmol_mg), SD_nmol_mg = sd(sum_nmol_mg)), by = tissue]

seed_polars_DGDG_species_avgSD <- seed_polars_DGDG_species[, .(avg_nmol_mg = mean(nmol_mg), SD_nmol_mg = sd(nmol_mg)), by = .(tissue, component_name)]

setDT(seed_polars_LPC_sum)

seed_polars_LPC_sum_avgSD <- seed_polars_LPC_sum[, .(avg_nmol_mg = mean(sum_nmol_mg), SD_nmol_mg = sd(sum_nmol_mg)), by = tissue]

seed_polars_LPC_species_avgSD <- seed_polars_LPC_species[, .(avg_nmol_mg = mean(nmol_mg), SD_nmol_mg = sd(nmol_mg)), by = .(tissue, component_name)]

setDT(seed_polars_LPE_sum)

seed_polars_LPE_sum_avgSD <- seed_polars_LPE_sum[, .(avg_nmol_mg = mean(sum_nmol_mg), SD_nmol_mg = sd(sum_nmol_mg)), by = tissue]

seed_polars_LPE_species_avgSD <- seed_polars_LPE_species[, .(avg_nmol_mg = mean(nmol_mg), SD_nmol_mg = sd(nmol_mg)), by = .(tissue, component_name)]

setDT(seed_polars_LPG_sum)

seed_polars_LPG_sum_avgSD <- seed_polars_LPG_sum[, .(avg_nmol_mg = mean(sum_nmol_mg), SD_nmol_mg = sd(sum_nmol_mg)), by = tissue]

seed_polars_LPG_species_avgSD <- seed_polars_LPG_species[, .(avg_nmol_mg = mean(nmol_mg), SD_nmol_mg = sd(nmol_mg)), by = .(tissue, component_name)]

setDT(seed_polars_LPI_sum)

seed_polars_LPI_sum_avgSD <- seed_polars_LPI_sum[, .(avg_nmol_mg = mean(sum_nmol_mg), SD_nmol_mg = sd(sum_nmol_mg)), by = tissue]

seed_polars_LPI_species_avgSD <- seed_polars_LPI_species[, .(avg_nmol_mg = mean(nmol_mg), SD_nmol_mg = sd(nmol_mg)), by = .(tissue, component_name)]

setDT(seed_polars_LPS_sum)

seed_polars_LPS_sum_avgSD <- seed_polars_LPS_sum[, .(avg_nmol_mg = mean(sum_nmol_mg), SD_nmol_mg = sd(sum_nmol_mg)), by = tissue]

seed_polars_LPS_species_avgSD <- seed_polars_LPS_species[, .(avg_nmol_mg = mean(nmol_mg), SD_nmol_mg = sd(nmol_mg)), by = .(tissue, component_name)]

setDT(seed_polars_MGDG_sum)

seed_polars_MGDG_sum_avgSD <- seed_polars_MGDG_sum[, .(avg_nmol_mg = mean(sum_nmol_mg), SD_nmol_mg = sd(sum_nmol_mg)), by = tissue]

seed_polars_MGDG_species_avgSD <- seed_polars_MGDG_species[, .(avg_nmol_mg = mean(nmol_mg), SD_nmol_mg = sd(nmol_mg)), by = .(tissue, component_name)]

setDT(seed_polars_PG_sum)

seed_polars_PG_sum_avgSD <- seed_polars_PG_sum[, .(avg_nmol_mg = mean(sum_nmol_mg), SD_nmol_mg = sd(sum_nmol_mg)), by = tissue]

seed_polars_PG_species_avgSD <- seed_polars_PG_species[, .(avg_nmol_mg = mean(nmol_mg), SD_nmol_mg = sd(nmol_mg)), by = .(tissue, component_name)]

setDT(seed_polars_PI_sum)

seed_polars_PI_sum_avgSD <- seed_polars_PI_sum[, .(avg_nmol_mg = mean(sum_nmol_mg), SD_nmol_mg = sd(sum_nmol_mg)), by = tissue]

seed_polars_PI_species_avgSD <- seed_polars_PI_species[, .(avg_nmol_mg = mean(nmol_mg), SD_nmol_mg = sd(nmol_mg)), by = .(tissue, component_name)]

setDT(seed_polars_PS_sum)

seed_polars_PS_sum_avgSD <- seed_polars_PS_sum[, .(avg_nmol_mg = mean(sum_nmol_mg), SD_nmol_mg = sd(sum_nmol_mg)), by = tissue]

seed_polars_PS_species_avgSD <- seed_polars_PS_species[, .(avg_nmol_mg = mean(nmol_mg), SD_nmol_mg = sd(nmol_mg)), by = .(tissue, component_name)]

setDT(seed_polars_SQDG_sum)

seed_polars_SQDG_sum_avgSD <- seed_polars_SQDG_sum[, .(avg_nmol_mg = mean(sum_nmol_mg), SD_nmol_mg = sd(sum_nmol_mg)), by = tissue]

seed_polars_SQDG_species_avgSD <- seed_polars_SQDG_species[, .(avg_nmol_mg = mean(nmol_mg), SD_nmol_mg = sd(nmol_mg)), by = .(tissue, component_name)]

# writing final data files (both sum and individual species)

write.table(seed_polars_PC_sum_avgSD, file = "seed_polars_PC_sum_avgSD.txt", quote = FALSE, row.names = FALSE, sep = "\t")

write.table(seed_polars_PC_species_avgSD, file = "seed_polars_PC_species_avgSD.txt", quote = FALSE, row.names = FALSE, sep = "\t")

write.table(seed_polars_PE_sum_avgSD, file = "seed_polars_PE_sum_avgSD.txt", quote = FALSE, row.names = FALSE, sep = "\t")

write.table(seed_polars_PE_species_avgSD, file = "seed_polars_PE_species_avgSD.txt", quote = FALSE, row.names = FALSE, sep = "\t")

write.table(seed_polars_DGDG_sum_avgSD, file = "seed_polars_DGDG_sum_avgSD.txt", quote = FALSE, row.names = FALSE, sep = "\t")

write.table(seed_polars_DGDG_species_avgSD, file = "seed_polars_DGDG_species_avgSD.txt", quote = FALSE, row.names = FALSE, sep = "\t")

write.table(seed_polars_LPC_sum_avgSD, file = "seed_polars_LPC_sum_avgSD.txt", quote = FALSE, row.names = FALSE, sep = "\t")

write.table(seed_polars_LPC_species_avgSD, file = "seed_polars_LPC_species_avgSD.txt", quote = FALSE, row.names = FALSE, sep = "\t")

write.table(seed_polars_LPE_sum_avgSD, file = "seed_polars_LPE_sum_avgSD.txt", quote = FALSE, row.names = FALSE, sep = "\t")

write.table(seed_polars_LPE_species_avgSD, file = "seed_polars_LPE_species_avgSD.txt", quote = FALSE, row.names = FALSE, sep = "\t")

write.table(seed_polars_LPG_sum_avgSD, file = "seed_polars_LPG_sum_avgSD.txt", quote = FALSE, row.names = FALSE, sep = "\t")

write.table(seed_polars_LPG_species_avgSD, file = "seed_polars_LPG_species_avgSD.txt", quote = FALSE, row.names = FALSE, sep = "\t")

write.table(seed_polars_LPI_sum_avgSD, file = "seed_polars_LPI_sum_avgSD.txt", quote = FALSE, row.names = FALSE, sep = "\t")

write.table(seed_polars_LPI_species_avgSD, file = "seed_polars_LPI_species_avgSD.txt", quote = FALSE, row.names = FALSE, sep = "\t")

write.table(seed_polars_LPS_sum_avgSD, file = "seed_polars_LPS_sum_avgSD.txt", quote = FALSE, row.names = FALSE, sep = "\t")

write.table(seed_polars_LPS_species_avgSD, file = "seed_polars_LPS_species_avgSD.txt", quote = FALSE, row.names = FALSE, sep = "\t")

write.table(seed_polars_MGDG_sum_avgSD, file = "seed_polars_MGDG_sum_avgSD.txt", quote = FALSE, row.names = FALSE, sep = "\t")

write.table(seed_polars_MGDG_species_avgSD, file = "seed_polars_MGDG_species_avgSD.txt", quote = FALSE, row.names = FALSE, sep = "\t")

write.table(seed_polars_PG_sum_avgSD, file = "seed_polars_PG_sum_avgSD.txt", quote = FALSE, row.names = FALSE, sep = "\t")

write.table(seed_polars_PG_species_avgSD, file = "seed_polars_PG_species_avgSD.txt", quote = FALSE, row.names = FALSE, sep = "\t")

write.table(seed_polars_PI_sum_avgSD, file = "seed_polars_PI_sum_avgSD.txt", quote = FALSE, row.names = FALSE, sep = "\t")

write.table(seed_polars_PI_species_avgSD, file = "seed_polars_PI_species_avgSD.txt", quote = FALSE, row.names = FALSE, sep = "\t")

write.table(seed_polars_PS_sum_avgSD, file = "seed_polars_PS_sum_avgSD.txt", quote = FALSE, row.names = FALSE, sep = "\t")

write.table(seed_polars_PS_species_avgSD, file = "seed_polars_PS_species_avgSD.txt", quote = FALSE, row.names = FALSE, sep = "\t")

write.table(seed_polars_SQDG_sum_avgSD, file = "seed_polars_SQDG_sum_avgSD.txt", quote = FALSE, row.names = FALSE, sep = "\t")

write.table(seed_polars_SQDG_species_avgSD, file = "seed_polars_SQDG_species_avgSD.txt", quote = FALSE, row.names = FALSE, sep = "\t")

# plotting avg summed classes with SD error bars

# PC

ggplot(seed_polars_PC_sum_avgSD) +

geom_col(mapping = aes(tissue, avg_nmol_mg)) +

scale_x_discrete(limits = c("14 DAP", "17 DAP", "20 DAP", "23 DAP", "mature", "germ")) +

geom_errorbar(mapping = aes(tissue, ymin = avg_nmol_mg - SD_nmol_mg, ymax = avg_nmol_mg + SD_nmol_mg), width = 0.2) +

labs(title = "Avg sum of PC", x = "tissue", y = "nmol / mg") +

theme_minimal() +

theme(plot.title = element_text(hjust = 0.5), axis.line = element_line(color = "black"), panel.grid.major = element_line(linetype = 0), panel.grid.minor = element_line(linetype = 0), axis.ticks = element_line(linetype = 1)) +

scale_y_continuous(expand = c(0, 0), limits = c(0, NA))

ggsave("avg_PC_sum.svg", width = 6, height = 4, units = "in", dpi = 300)

# PE

ggplot(seed_polars_PE_sum_avgSD) +

geom_col(mapping = aes(tissue, avg_nmol_mg)) +

scale_x_discrete(limits = c("14 DAP", "17 DAP", "20 DAP", "23 DAP", "mature", "germ")) +

geom_errorbar(mapping = aes(tissue, ymin = avg_nmol_mg - SD_nmol_mg, ymax = avg_nmol_mg + SD_nmol_mg), width = 0.2) +

labs(title = "Avg sum of PE", x = "tissue", y = "nmol / mg") +

theme_minimal() +

theme(plot.title = element_text(hjust = 0.5), axis.line = element_line(color = "black"), panel.grid.major = element_line(linetype = 0), panel.grid.minor = element_line(linetype = 0), axis.ticks = element_line(linetype = 1)) +

scale_y_continuous(expand = c(0, 0), limits = c(0, NA))

ggsave("avg_PE_sum.svg", width = 6, height = 4, units = "in", dpi = 300)

# DGDG

ggplot(seed_polars_DGDG_sum_avgSD) +

geom_col(mapping = aes(tissue, avg_nmol_mg)) +

scale_x_discrete(limits = c("14 DAP", "17 DAP", "20 DAP", "23 DAP", "mature", "germ")) +

geom_errorbar(mapping = aes(tissue, ymin = avg_nmol_mg - SD_nmol_mg, ymax = avg_nmol_mg + SD_nmol_mg), width = 0.2) +

labs(title = "Avg sum of DGDG", x = "tissue", y = "nmol / mg") +

theme_minimal() +

theme(plot.title = element_text(hjust = 0.5), axis.line = element_line(color = "black"), panel.grid.major = element_line(linetype = 0), panel.grid.minor = element_line(linetype = 0), axis.ticks = element_line(linetype = 1)) +

scale_y_continuous(expand = c(0, 0), limits = c(0, NA))

ggsave("avg_DGDG_sum.svg", width = 6, height = 4, units = "in", dpi = 300)

# LPC

ggplot(seed_polars_LPC_sum_avgSD) +

geom_col(mapping = aes(tissue, avg_nmol_mg)) +

scale_x_discrete(limits = c("14 DAP", "17 DAP", "20 DAP", "23 DAP", "mature", "germ")) +

geom_errorbar(mapping = aes(tissue, ymin = avg_nmol_mg - SD_nmol_mg, ymax = avg_nmol_mg + SD_nmol_mg), width = 0.2) +

labs(title = "Avg sum of LPC", x = "tissue", y = "nmol / mg") +

theme_minimal() +

theme(plot.title = element_text(hjust = 0.5), axis.line = element_line(color = "black"), panel.grid.major = element_line(linetype = 0), panel.grid.minor = element_line(linetype = 0), axis.ticks = element_line(linetype = 1)) +

scale_y_continuous(expand = c(0, 0), limits = c(0, NA))

ggsave("avg_LPC_sum.svg", width = 6, height = 4, units = "in", dpi = 300)

# LPE

ggplot(seed_polars_LPE_sum_avgSD) +

geom_col(mapping = aes(tissue, avg_nmol_mg)) +

scale_x_discrete(limits = c("14 DAP", "17 DAP", "20 DAP", "23 DAP", "mature", "germ")) +

geom_errorbar(mapping = aes(tissue, ymin = avg_nmol_mg - SD_nmol_mg, ymax = avg_nmol_mg + SD_nmol_mg), width = 0.2) +

labs(title = "Avg sum of LPE", x = "tissue", y = "nmol / mg") +

theme_minimal() +

theme(plot.title = element_text(hjust = 0.5), axis.line = element_line(color = "black"), panel.grid.major = element_line(linetype = 0), panel.grid.minor = element_line(linetype = 0), axis.ticks = element_line(linetype = 1)) +

scale_y_continuous(expand = c(0, 0), limits = c(0, NA))

ggsave("avg_LPE_sum.svg", width = 6, height = 4, units = "in", dpi = 300)

# LPG

ggplot(seed_polars_LPG_sum_avgSD) +

geom_col(mapping = aes(tissue, avg_nmol_mg)) +

scale_x_discrete(limits = c("14 DAP", "17 DAP", "20 DAP", "23 DAP", "mature", "germ")) +

geom_errorbar(mapping = aes(tissue, ymin = avg_nmol_mg - SD_nmol_mg, ymax = avg_nmol_mg + SD_nmol_mg), width = 0.2) +

labs(title = "Avg sum of LPG", x = "tissue", y = "nmol / mg") +

theme_minimal() +

theme(plot.title = element_text(hjust = 0.5), axis.line = element_line(color = "black"), panel.grid.major = element_line(linetype = 0), panel.grid.minor = element_line(linetype = 0), axis.ticks = element_line(linetype = 1)) +

scale_y_continuous(expand = c(0, 0), limits = c(0, NA))

ggsave("avg_LPG_sum.svg", width = 6, height = 4, units = "in", dpi = 300)

# LPI

ggplot(seed_polars_LPI_sum_avgSD) +

geom_col(mapping = aes(tissue, avg_nmol_mg)) +

scale_x_discrete(limits = c("14 DAP", "17 DAP", "20 DAP", "23 DAP", "mature", "germ")) +

geom_errorbar(mapping = aes(tissue, ymin = avg_nmol_mg - SD_nmol_mg, ymax = avg_nmol_mg + SD_nmol_mg), width = 0.2) +

labs(title = "Avg sum of LPI", x = "tissue", y = "nmol / mg") +

theme_minimal() +

theme(plot.title = element_text(hjust = 0.5), axis.line = element_line(color = "black"), panel.grid.major = element_line(linetype = 0), panel.grid.minor = element_line(linetype = 0), axis.ticks = element_line(linetype = 1)) +

scale_y_continuous(expand = c(0, 0), limits = c(0, NA))

ggsave("avg_LPI_sum.svg", width = 6, height = 4, units = "in", dpi = 300)

# LPS

ggplot(seed_polars_LPS_sum_avgSD) +

geom_col(mapping = aes(tissue, avg_nmol_mg)) +

scale_x_discrete(limits = c("14 DAP", "17 DAP", "20 DAP", "23 DAP", "mature", "germ")) +

geom_errorbar(mapping = aes(tissue, ymin = avg_nmol_mg - SD_nmol_mg, ymax = avg_nmol_mg + SD_nmol_mg), width = 0.2) +

labs(title = "Avg sum of LPS", x = "tissue", y = "nmol / mg") +

theme_minimal() +

theme(plot.title = element_text(hjust = 0.5), axis.line = element_line(color = "black"), panel.grid.major = element_line(linetype = 0), panel.grid.minor = element_line(linetype = 0), axis.ticks = element_line(linetype = 1)) +

scale_y_continuous(expand = c(0, 0), limits = c(0, NA))

ggsave("avg_LPS_sum.svg", width = 6, height = 4, units = "in", dpi = 300)

# MGDG

ggplot(seed_polars_MGDG_sum_avgSD) +

geom_col(mapping = aes(tissue, avg_nmol_mg)) +

scale_x_discrete(limits = c("14 DAP", "17 DAP", "20 DAP", "23 DAP", "mature", "germ")) +

geom_errorbar(mapping = aes(tissue, ymin = avg_nmol_mg - SD_nmol_mg, ymax = avg_nmol_mg + SD_nmol_mg), width = 0.2) +

labs(title = "Avg sum of MGDG", x = "tissue", y = "nmol / mg") +

theme_minimal() +

theme(plot.title = element_text(hjust = 0.5), axis.line = element_line(color = "black"), panel.grid.major = element_line(linetype = 0), panel.grid.minor = element_line(linetype = 0), axis.ticks = element_line(linetype = 1)) +

scale_y_continuous(expand = c(0, 0), limits = c(0, NA))

ggsave("avg_MGDG_sum.svg", width = 6, height = 4, units = "in", dpi = 300)

# PG

ggplot(seed_polars_PG_sum_avgSD) +

geom_col(mapping = aes(tissue, avg_nmol_mg)) +

scale_x_discrete(limits = c("14 DAP", "17 DAP", "20 DAP", "23 DAP", "mature", "germ")) +

geom_errorbar(mapping = aes(tissue, ymin = avg_nmol_mg - SD_nmol_mg, ymax = avg_nmol_mg + SD_nmol_mg), width = 0.2) +

labs(title = "Avg sum of PG", x = "tissue", y = "nmol / mg") +

theme_minimal() +

theme(plot.title = element_text(hjust = 0.5), axis.line = element_line(color = "black"), panel.grid.major = element_line(linetype = 0), panel.grid.minor = element_line(linetype = 0), axis.ticks = element_line(linetype = 1)) +

scale_y_continuous(expand = c(0, 0), limits = c(0, NA))

ggsave("avg_PG_sum.svg", width = 6, height = 4, units = "in", dpi = 300)

# PI

ggplot(seed_polars_PI_sum_avgSD) +

geom_col(mapping = aes(tissue, avg_nmol_mg)) +

scale_x_discrete(limits = c("14 DAP", "17 DAP", "20 DAP", "23 DAP", "mature", "germ")) +

geom_errorbar(mapping = aes(tissue, ymin = avg_nmol_mg - SD_nmol_mg, ymax = avg_nmol_mg + SD_nmol_mg), width = 0.2) +

labs(title = "Avg sum of PI", x = "tissue", y = "nmol / mg") +

theme_minimal() +

theme(plot.title = element_text(hjust = 0.5), axis.line = element_line(color = "black"), panel.grid.major = element_line(linetype = 0), panel.grid.minor = element_line(linetype = 0), axis.ticks = element_line(linetype = 1)) +

scale_y_continuous(expand = c(0, 0), limits = c(0, NA))

ggsave("avg_PI_sum.svg", width = 6, height = 4, units = "in", dpi = 300)

# PS

ggplot(seed_polars_PS_sum_avgSD) +

geom_col(mapping = aes(tissue, avg_nmol_mg)) +

scale_x_discrete(limits = c("14 DAP", "17 DAP", "20 DAP", "23 DAP", "mature", "germ")) +

geom_errorbar(mapping = aes(tissue, ymin = avg_nmol_mg - SD_nmol_mg, ymax = avg_nmol_mg + SD_nmol_mg), width = 0.2) +

labs(title = "Avg sum of PS", x = "tissue", y = "nmol / mg") +

theme_minimal() +

theme(plot.title = element_text(hjust = 0.5), axis.line = element_line(color = "black"), panel.grid.major = element_line(linetype = 0), panel.grid.minor = element_line(linetype = 0), axis.ticks = element_line(linetype = 1)) +

scale_y_continuous(expand = c(0, 0), limits = c(0, NA))

ggsave("avg_PS_sum.svg", width = 6, height = 4, units = "in", dpi = 300)

# SQDG

ggplot(seed_polars_SQDG_sum_avgSD) +

geom_col(mapping = aes(tissue, avg_nmol_mg)) +

scale_x_discrete(limits = c("14 DAP", "17 DAP", "20 DAP", "23 DAP", "mature", "germ")) +

geom_errorbar(mapping = aes(tissue, ymin = avg_nmol_mg - SD_nmol_mg, ymax = avg_nmol_mg + SD_nmol_mg), width = 0.2) +

labs(title = "Avg sum of SQDG", x = "tissue", y = "nmol / mg") +

theme_minimal() +

theme(plot.title = element_text(hjust = 0.5), axis.line = element_line(color = "black"), panel.grid.major = element_line(linetype = 0), panel.grid.minor = element_line(linetype = 0), axis.ticks = element_line(linetype = 1)) +

scale_y_continuous(expand = c(0, 0), limits = c(0, NA))

ggsave("avg_SQDG_sum.svg", width = 6, height = 4, units = "in", dpi = 300)

# plotting avg molecular species SD error bars

# PC

ggplot(seed_polars_PC_species_avgSD, aes(fill = factor(tissue, levels = c("14 DAP", "17 DAP", "20 DAP", "23 DAP", "mature", "germ")), x = component_name, y = avg_nmol_mg)) +

geom_col(position = "dodge") +

geom_errorbar(mapping = aes(ymin = avg_nmol_mg - SD_nmol_mg, ymax = avg_nmol_mg + SD_nmol_mg), position = "dodge") +

theme_minimal() +

theme(axis.text.x = element_text(angle = -90, vjust = 0.5, hjust = 1), plot.title = element_text(hjust = 0.5)) +

labs(title = "PC molecular species", x = "PC molecular species", y = "nmol / mg", fill = "tissue")

ggsave("avg_PC_species.svg", width = 10, height = 4, units = "in", dpi = 300)

# PE

ggplot(seed_polars_PE_species_avgSD, aes(fill = factor(tissue, levels = c("14 DAP", "17 DAP", "20 DAP", "23 DAP", "mature", "germ")), x = component_name, y = avg_nmol_mg)) +

geom_col(position = "dodge") +

geom_errorbar(mapping = aes(ymin = avg_nmol_mg - SD_nmol_mg, ymax = avg_nmol_mg + SD_nmol_mg), position = "dodge") +

theme_minimal() +

theme(axis.text.x = element_text(angle = -90, vjust = 0.5, hjust = 1), plot.title = element_text(hjust = 0.5)) +

labs(title = "PE molecular species", x = "PE molecular species", y = "nmol / mg", fill = "tissue")

ggsave("avg_PE_species.svg", width = 10, height = 4, units = "in", dpi = 300)

# DGDG

ggplot(seed_polars_DGDG_species_avgSD, aes(fill = factor(tissue, levels = c("14 DAP", "17 DAP", "20 DAP", "23 DAP", "mature", "germ")), x = component_name, y = avg_nmol_mg)) +

geom_col(position = "dodge") +

geom_errorbar(mapping = aes(ymin = avg_nmol_mg - SD_nmol_mg, ymax = avg_nmol_mg + SD_nmol_mg), position = "dodge") +

theme_minimal() +

theme(axis.text.x = element_text(angle = -90, vjust = 0.5, hjust = 1), plot.title = element_text(hjust = 0.5)) +

labs(title = "DGDG molecular species", x = "DGDG molecular species", y = "nmol / mg", fill = "tissue")

ggsave("avg_DGDG_species.svg", width = 10, height = 4, units = "in", dpi = 300)

# LPC

ggplot(seed_polars_LPC_species_avgSD, aes(fill = factor(tissue, levels = c("14 DAP", "17 DAP", "20 DAP", "23 DAP", "mature", "germ")), x = component_name, y = avg_nmol_mg)) +

geom_col(position = "dodge") +

geom_errorbar(mapping = aes(ymin = avg_nmol_mg - SD_nmol_mg, ymax = avg_nmol_mg + SD_nmol_mg), position = "dodge") +

theme_minimal() +

theme(axis.text.x = element_text(angle = -90, vjust = 0.5, hjust = 1), plot.title = element_text(hjust = 0.5)) +

labs(title = "LPC molecular species", x = "LPC molecular species", y = "nmol / mg", fill = "tissue")

ggsave("avg_LPC_species.svg", width = 10, height = 4, units = "in", dpi = 300)

# LPE

ggplot(seed_polars_LPE_species_avgSD, aes(fill = factor(tissue, levels = c("14 DAP", "17 DAP", "20 DAP", "23 DAP", "mature", "germ")), x = component_name, y = avg_nmol_mg)) +

geom_col(position = "dodge") +

geom_errorbar(mapping = aes(ymin = avg_nmol_mg - SD_nmol_mg, ymax = avg_nmol_mg + SD_nmol_mg), position = "dodge") +

theme_minimal() +

theme(axis.text.x = element_text(angle = -90, vjust = 0.5, hjust = 1), plot.title = element_text(hjust = 0.5)) +

labs(title = "LPE molecular species", x = "LPE molecular species", y = "nmol / mg", fill = "tissue")

ggsave("avg_LPE_species.svg", width = 10, height = 4, units = "in", dpi = 300)

# LPG

ggplot(seed_polars_LPG_species_avgSD, aes(fill = factor(tissue, levels = c("14 DAP", "17 DAP", "20 DAP", "23 DAP", "mature", "germ")), x = component_name, y = avg_nmol_mg)) +

geom_col(position = "dodge") +

geom_errorbar(mapping = aes(ymin = avg_nmol_mg - SD_nmol_mg, ymax = avg_nmol_mg + SD_nmol_mg), position = "dodge") +

theme_minimal() +

theme(axis.text.x = element_text(angle = -90, vjust = 0.5, hjust = 1), plot.title = element_text(hjust = 0.5)) +

labs(title = "LPG molecular species", x = "LPG molecular species", y = "nmol / mg", fill = "tissue")

ggsave("avg_LPG_species.svg", width = 10, height = 4, units = "in", dpi = 300)

# LPI

ggplot(seed_polars_LPI_species_avgSD, aes(fill = factor(tissue, levels = c("14 DAP", "17 DAP", "20 DAP", "23 DAP", "mature", "germ")), x = component_name, y = avg_nmol_mg)) +

geom_col(position = "dodge") +

geom_errorbar(mapping = aes(ymin = avg_nmol_mg - SD_nmol_mg, ymax = avg_nmol_mg + SD_nmol_mg), position = "dodge") +

theme_minimal() +

theme(axis.text.x = element_text(angle = -90, vjust = 0.5, hjust = 1), plot.title = element_text(hjust = 0.5)) +

labs(title = "LPI molecular species", x = "LPI molecular species", y = "nmol / mg", fill = "tissue")

ggsave("avg_LPI_species.svg", width = 10, height = 4, units = "in", dpi = 300)

# LPS

ggplot(seed_polars_LPS_species_avgSD, aes(fill = factor(tissue, levels = c("14 DAP", "17 DAP", "20 DAP", "23 DAP", "mature", "germ")), x = component_name, y = avg_nmol_mg)) +

geom_col(position = "dodge") +

geom_errorbar(mapping = aes(ymin = avg_nmol_mg - SD_nmol_mg, ymax = avg_nmol_mg + SD_nmol_mg), position = "dodge") +

theme_minimal() +

theme(axis.text.x = element_text(angle = -90, vjust = 0.5, hjust = 1), plot.title = element_text(hjust = 0.5)) +

labs(title = "LPS molecular species", x = "LPS molecular species", y = "nmol / mg", fill = "tissue")

ggsave("avg_LPS_species.svg", width = 10, height = 4, units = "in", dpi = 300)

# MGDG

ggplot(seed_polars_MGDG_species_avgSD, aes(fill = factor(tissue, levels = c("14 DAP", "17 DAP", "20 DAP", "23 DAP", "mature", "germ")), x = component_name, y = avg_nmol_mg)) +

geom_col(position = "dodge") +

geom_errorbar(mapping = aes(ymin = avg_nmol_mg - SD_nmol_mg, ymax = avg_nmol_mg + SD_nmol_mg), position = "dodge") +

theme_minimal() +

theme(axis.text.x = element_text(angle = -90, vjust = 0.5, hjust = 1), plot.title = element_text(hjust = 0.5)) +

labs(title = "MGDG molecular species", x = "MGDG molecular species", y = "nmol / mg", fill = "tissue")

ggsave("avg_MGDG_species.svg", width = 10, height = 4, units = "in", dpi = 300)

# PG

ggplot(seed_polars_PG_species_avgSD, aes(fill = factor(tissue, levels = c("14 DAP", "17 DAP", "20 DAP", "23 DAP", "mature", "germ")), x = component_name, y = avg_nmol_mg)) +

geom_col(position = "dodge") +

geom_errorbar(mapping = aes(ymin = avg_nmol_mg - SD_nmol_mg, ymax = avg_nmol_mg + SD_nmol_mg), position = "dodge") +

theme_minimal() +

theme(axis.text.x = element_text(angle = -90, vjust = 0.5, hjust = 1), plot.title = element_text(hjust = 0.5)) +

labs(title = "PG molecular species", x = "PG molecular species", y = "nmol / mg", fill = "tissue")

ggsave("avg_PG_species.svg", width = 10, height = 4, units = "in", dpi = 300)

# PI

ggplot(seed_polars_PI_species_avgSD, aes(fill = factor(tissue, levels = c("14 DAP", "17 DAP", "20 DAP", "23 DAP", "mature", "germ")), x = component_name, y = avg_nmol_mg)) +

geom_col(position = "dodge") +

geom_errorbar(mapping = aes(ymin = avg_nmol_mg - SD_nmol_mg, ymax = avg_nmol_mg + SD_nmol_mg), position = "dodge") +

theme_minimal() +

theme(axis.text.x = element_text(angle = -90, vjust = 0.5, hjust = 1), plot.title = element_text(hjust = 0.5)) +

labs(title = "PI molecular species", x = "PI molecular species", y = "nmol / mg", fill = "tissue")

ggsave("avg_PI_species.svg", width = 10, height = 4, units = "in", dpi = 300)

# PS

ggplot(seed_polars_PS_species_avgSD, aes(fill = factor(tissue, levels = c("14 DAP", "17 DAP", "20 DAP", "23 DAP", "mature", "germ")), x = component_name, y = avg_nmol_mg)) +

geom_col(position = "dodge") +

geom_errorbar(mapping = aes(ymin = avg_nmol_mg - SD_nmol_mg, ymax = avg_nmol_mg + SD_nmol_mg), position = "dodge") +

theme_minimal() +

theme(axis.text.x = element_text(angle = -90, vjust = 0.5, hjust = 1), plot.title = element_text(hjust = 0.5)) +

labs(title = "PS molecular species", x = "PS molecular species", y = "nmol / mg", fill = "tissue")

ggsave("avg_PS_species.svg", width = 10, height = 4, units = "in", dpi = 300)

# SQDG

ggplot(seed_polars_SQDG_species_avgSD, aes(fill = factor(tissue, levels = c("14 DAP", "17 DAP", "20 DAP", "23 DAP", "mature", "germ")), x = component_name, y = avg_nmol_mg)) +

geom_col(position = "dodge") +

geom_errorbar(mapping = aes(ymin = avg_nmol_mg - SD_nmol_mg, ymax = avg_nmol_mg + SD_nmol_mg), position = "dodge") +

theme_minimal() +

theme(axis.text.x = element_text(angle = -90, vjust = 0.5, hjust = 1), plot.title = element_text(hjust = 0.5)) +

labs(title = "SQDG molecular species", x = "SQDG molecular species", y = "nmol / mg", fill = "tissue")

ggsave("avg_SQDG_species.svg", width = 10, height = 4, units = "in", dpi = 300)

# creating Metaboanalyst-compatible data table

sample_no <- data.table("tissue" = c("14 DAP", "17 DAP", "20 DAP", "23 DAP", "mature", "germ"), "label" = c(0,1,2,3,4,5))

# PC

seed_polars_PC_species[, sample := do.call(paste, c(.SD, sep = "_")), .SDcols = c("tissue", "sample_no")]

seed_polars_PC_species <- seed_polars_PC_species[sample_no, on = .(tissue), nomatch = 0]

seed_polars_PC_metaboanalyst <- dcast(seed_polars_PC_species, sample + label ~ component_name, value.var = "nmol_mg")

setorder(seed_polars_PC_metaboanalyst, label)

write.table(seed_polars_PC_metaboanalyst, file = "seed_polars_PC_metaboanalyst.txt", quote = FALSE, row.names = FALSE, sep = "\t")

# PE

seed_polars_PE_species[, sample := do.call(paste, c(.SD, sep = "_")), .SDcols = c("tissue", "sample_no")]

seed_polars_PE_species <- seed_polars_PE_species[sample_no, on = .(tissue), nomatch = 0]

seed_polars_PE_metaboanalyst <- dcast(seed_polars_PE_species, sample + label ~ component_name, value.var = "nmol_mg")

setorder(seed_polars_PE_metaboanalyst, label)

write.table(seed_polars_PE_metaboanalyst, file = "seed_polars_PE_metaboanalyst.txt", quote = FALSE, row.names = FALSE, sep = "\t")

# DGDG

seed_polars_DGDG_species[, sample := do.call(paste, c(.SD, sep = "_")), .SDcols = c("tissue", "sample_no")]

seed_polars_DGDG_species <- seed_polars_DGDG_species[sample_no, on = .(tissue), nomatch = 0]

seed_polars_DGDG_metaboanalyst <- dcast(seed_polars_DGDG_species, sample + label ~ component_name, value.var = "nmol_mg")

setorder(seed_polars_DGDG_metaboanalyst, label)

write.table(seed_polars_DGDG_metaboanalyst, file = "seed_polars_DGDG_metaboanalyst.txt", quote = FALSE, row.names = FALSE, sep = "\t")

# LPC

seed_polars_LPC_species[, sample := do.call(paste, c(.SD, sep = "_")), .SDcols = c("tissue", "sample_no")]

seed_polars_LPC_species <- seed_polars_LPC_species[sample_no, on = .(tissue), nomatch = 0]

seed_polars_LPC_metaboanalyst <- dcast(seed_polars_LPC_species, sample + label ~ component_name, value.var = "nmol_mg")

setorder(seed_polars_LPC_metaboanalyst, label)

write.table(seed_polars_LPC_metaboanalyst, file = "seed_polars_LPC_metaboanalyst.txt", quote = FALSE, row.names = FALSE, sep = "\t")

# LPE

seed_polars_LPE_species[, sample := do.call(paste, c(.SD, sep = "_")), .SDcols = c("tissue", "sample_no")]

seed_polars_LPE_species <- seed_polars_LPE_species[sample_no, on = .(tissue), nomatch = 0]

seed_polars_LPE_metaboanalyst <- dcast(seed_polars_LPE_species, sample + label ~ component_name, value.var = "nmol_mg")

setorder(seed_polars_LPE_metaboanalyst, label)

write.table(seed_polars_LPE_metaboanalyst, file = "seed_polars_LPE_metaboanalyst.txt", quote = FALSE, row.names = FALSE, sep = "\t")

# LPG

seed_polars_LPG_species[, sample := do.call(paste, c(.SD, sep = "_")), .SDcols = c("tissue", "sample_no")]

seed_polars_LPG_species <- seed_polars_LPG_species[sample_no, on = .(tissue), nomatch = 0]

seed_polars_LPG_metaboanalyst <- dcast(seed_polars_LPG_species, sample + label ~ component_name, value.var = "nmol_mg")

setorder(seed_polars_LPG_metaboanalyst, label)

write.table(seed_polars_LPG_metaboanalyst, file = "seed_polars_LPG_metaboanalyst.txt", quote = FALSE, row.names = FALSE, sep = "\t")

# LPI

seed_polars_LPI_species[, sample := do.call(paste, c(.SD, sep = "_")), .SDcols = c("tissue", "sample_no")]

seed_polars_LPI_species <- seed_polars_LPI_species[sample_no, on = .(tissue), nomatch = 0]

seed_polars_LPI_metaboanalyst <- dcast(seed_polars_LPI_species, sample + label ~ component_name, value.var = "nmol_mg")

setorder(seed_polars_LPI_metaboanalyst, label)

write.table(seed_polars_LPI_metaboanalyst, file = "seed_polars_LPI_metaboanalyst.txt", quote = FALSE, row.names = FALSE, sep = "\t")

# LPS

seed_polars_LPS_species[, sample := do.call(paste, c(.SD, sep = "_")), .SDcols = c("tissue", "sample_no")]

seed_polars_LPS_species <- seed_polars_LPS_species[sample_no, on = .(tissue), nomatch = 0]

seed_polars_LPS_metaboanalyst <- dcast(seed_polars_LPS_species, sample + label ~ component_name, value.var = "nmol_mg")

setorder(seed_polars_LPS_metaboanalyst, label)

write.table(seed_polars_LPS_metaboanalyst, file = "seed_polars_LPS_metaboanalyst.txt", quote = FALSE, row.names = FALSE, sep = "\t")

# MGDG

seed_polars_MGDG_species[, sample := do.call(paste, c(.SD, sep = "_")), .SDcols = c("tissue", "sample_no")]

seed_polars_MGDG_species <- seed_polars_MGDG_species[sample_no, on = .(tissue), nomatch = 0]

seed_polars_MGDG_metaboanalyst <- dcast(seed_polars_MGDG_species, sample + label ~ component_name, value.var = "nmol_mg")

setorder(seed_polars_MGDG_metaboanalyst, label)

write.table(seed_polars_MGDG_metaboanalyst, file = "seed_polars_MGDG_metaboanalyst.txt", quote = FALSE, row.names = FALSE, sep = "\t")

# PG

seed_polars_PG_species[, sample := do.call(paste, c(.SD, sep = "_")), .SDcols = c("tissue", "sample_no")]

seed_polars_PG_species <- seed_polars_PG_species[sample_no, on = .(tissue), nomatch = 0]

seed_polars_PG_metaboanalyst <- dcast(seed_polars_PG_species, sample + label ~ component_name, value.var = "nmol_mg")

setorder(seed_polars_PG_metaboanalyst, label)

write.table(seed_polars_PG_metaboanalyst, file = "seed_polars_PG_metaboanalyst.txt", quote = FALSE, row.names = FALSE, sep = "\t")

# PI

seed_polars_PI_species[, sample := do.call(paste, c(.SD, sep = "_")), .SDcols = c("tissue", "sample_no")]

seed_polars_PI_species <- seed_polars_PI_species[sample_no, on = .(tissue), nomatch = 0]

seed_polars_PI_metaboanalyst <- dcast(seed_polars_PI_species, sample + label ~ component_name, value.var = "nmol_mg")

setorder(seed_polars_PI_metaboanalyst, label)

write.table(seed_polars_PI_metaboanalyst, file = "seed_polars_PI_metaboanalyst.txt", quote = FALSE, row.names = FALSE, sep = "\t")

# PS

seed_polars_PS_species[, sample := do.call(paste, c(.SD, sep = "_")), .SDcols = c("tissue", "sample_no")]

seed_polars_PS_species <- seed_polars_PS_species[sample_no, on = .(tissue), nomatch = 0]

seed_polars_PS_metaboanalyst <- dcast(seed_polars_PS_species, sample + label ~ component_name, value.var = "nmol_mg")

setorder(seed_polars_PS_metaboanalyst, label)

write.table(seed_polars_PS_metaboanalyst, file = "seed_polars_PS_metaboanalyst.txt", quote = FALSE, row.names = FALSE, sep = "\t")

# SQDG

seed_polars_SQDG_species[, sample := do.call(paste, c(.SD, sep = "_")), .SDcols = c("tissue", "sample_no")]

seed_polars_SQDG_species <- seed_polars_SQDG_species[sample_no, on = .(tissue), nomatch = 0]

seed_polars_SQDG_metaboanalyst <- dcast(seed_polars_SQDG_species, sample + label ~ component_name, value.var = "nmol_mg")

setorder(seed_polars_SQDG_metaboanalyst, label)

write.table(seed_polars_SQDG_metaboanalyst, file = "seed_polars_SQDG_metaboanalyst.txt", quote = FALSE, row.names = FALSE, sep = "\t")
