# Supplementary material for: A lipidomics platform to analyze the fatty acid compositions of non-polar and polar lipid molecular species from plant tissues: Examples from developing seeds and seedlings of pennycress (Thlaspi arvense)
Source: Front Plant Sci. 2022 Nov 9;13:1038161. doi: 10.3389/fpls.2022.1038161 (PMC9682148; doi:10.3389/fpls.2022.1038161)
Supplement: Supplementary file 3 [file DataSheet_3.docx]

# Neutral lipidomics data processing script

# Order of components in R script + stds

# MAG stds: "MAG-17:0" "MAG-17:1" "MAG-19:0" "MAG-19:1" "MAG-19:2" "MAG-21:0"

# DAG stds: "DAG-31:1d5-FA14:1_17:0", "DAG-35:1d5-FA17:0_18:1", "DAG-39:4d5-FA22:4_17:0", "DAG-33:1d5-FA16:1_17:0", "DAG-37:3d5-FA20:3_17:0"

# TAG stds: "TAG-45:1d5-FA14:0 (14:0/17:1/14:0)", "TAG-53:3d5-FA18:1 (18:1/17:1/18:1)", "TAG-57:4d5-FA18:1 (18:1/21:2/18:1)", "TAG-41:0d5-FA14:0 (14:0/13:0/14:0)", "TAG-43:1d5-FA14:0 (14:0/15:1/14:0)", "TAG-47:1d5-FA16:0 (16:0/15:1/16:0)", "TAG-49:1d5-FA16:0 (16:0/17:1/16:0)", "TAG-51:2d5-FA16:0 (16:0/19:2/16:0)", "TAG-55:4d5-FA18:1 (18:1/19:2/18:1)"

# column headings in data file

# "sample_id" "sample_no" "tissue" "mrm_component_name" "component_name" "component_group" "mrm" "area" "height" "rt" "baseline" "tissue_wt"

# setting working directory containing data

setwd("PATH TO YOUR DATA FILE")

# loading libraries

library(tidyverse)

library(data.table)

# setting seed_neutrals as df

seed_neutrals <- read.delim(file = "FILENAME OF YOUR DATA FILE")

# separating classes into individual dfs

seed_neutrals_MAG <- filter(seed_neutrals, component_group == "MAG")

seed_neutrals_DAG <- filter(seed_neutrals, component_group == "DAG")

seed_neutrals_TAG <- filter(seed_neutrals, component_group == "TAG")

# dividing peak area of TAG molecular species by number of duplicate FAs (e.g. "TAG-58:5-FA18:2_(18:2/18:2/22:1)" is divided by 2)

setDT(seed_neutrals_TAG)

seed_neutrals_TAG[component_name %in% c("TAG-58:5-FA18:2_(18:2/18:2/22:1)", "TAG-58:3-FA20:1_(20:1/20:1/18:1)", "TAG-56:5-FA18:2_(18:2/18:2/20:1)", "TAG-54:7-FA18:2_(18:2/18:2/18:3)", "TAG-54:5-FA18:2_(18:2/18:2/18:1)", "TAG-54:2-FA18:1_(18:1/18:1/18:0)", "TAG-52:4-FA18:2_(18:2/18:2/16:0)", "TAG-58:5-FA20:1_(20:1/20:1/18:3)", "TAG-64:3-FA22:1_(22:1/22:1/20:1)", "TAG-64:3-FA24:1_(24:1/20:1/20:1)"), area := area / 2]

seed_neutrals_TAG[component_name == "TAG-54:3-FA18:1_(18:1/18:1/18:1)", area := area / 3]

seed_neutrals_TAG[component_name == "TAG-54:6-FA18:2_(18:2/18:2/18:2)", area := area / 3]

# writing class files into '\t' delimited .txt files

write.table(seed_neutrals_MAG, file = "seed_neutrals_MAG.txt", quote = FALSE, row.names = FALSE, sep = "\t")

write.table(seed_neutrals_DAG, file = "seed_neutrals_DAG.txt", quote = FALSE, row.names = FALSE, sep = "\t")

write.table(seed_neutrals_TAG, file = "seed_neutrals_TAG.txt", quote = FALSE, row.names = FALSE, sep = "\t")

# separating molecular species without internal standards

setDT(seed_neutrals_MAG)

seed_neutrals_MAG_species <- seed_neutrals_MAG[!(component_name %in% c("MAG-17:0", "MAG-17:1", "MAG-19:0", "MAG-19:1", "MAG-19:2", "MAG-21:0"))]

setDT(seed_neutrals_DAG)

seed_neutrals_DAG_species <- seed_neutrals_DAG[component_name %in% c("DAG-32:0-FA16:0_16:0", "DAG-32:1-FA16:0_16:1", "DAG-32:2-FA16:1_16:1", "DAG-34:0-FA16:0_18:0", "DAG-34:1-FA18:1_16:0", "DAG-34:2-FA18:0_16:2", "DAG-34:2-FA18:1_16:1", "DAG-34:2-FA18:2_16:0", "DAG-34:3-FA18:2_16:1", "DAG-34:3-FA18:3_16:0", "DAG-34:4-FA18:3_16:1", "DAG-36:0-FA18:0_18:0", "DAG-36:0-FA20:0_16:0", "DAG-36:1-FA18:0_18:1", "DAG-36:1-FA20:0_16:1", "DAG-36:1-FA20:1_16:0", "DAG-36:2-FA18:1_18:1", "DAG-36:2-FA18:2_18:0", "DAG-36:2-FA20:1_16:1", "DAG-36:3-FA16:1_20:2", "DAG-36:3-FA18:2_18:1", "DAG-36:4-FA18:1_18:3", "DAG-36:4-FA18:2_18:2", "DAG-36:5-FA18:2_18:3", "DAG-36:6-FA18:3_18:3", "DAG-38:0-FA16:0_22:0", "DAG-38:0-FA18:0_20:0", "DAG-38:1-FA16:0_22:1", "DAG-38:1-FA16:1_22:0", "DAG-38:1-FA18:0_20:1", "DAG-38:1-FA18:1_20:0", "DAG-38:2-FA16:1_22:1", "DAG-38:2-FA18:0_20:2", "DAG-38:2-FA18:1_20:1", "DAG-38:2-FA18:2_20:0", "DAG-38:3-FA18:1_20:2", "DAG-38:3-FA18:2_20:1", "DAG-38:3-FA18:3_20:0", "DAG-38:4-FA18:2_20:2", "DAG-38:4-FA18:3_20:1", "DAG-38:5-FA18:3_20:2", "DAG-40:0-FA16:0_24:0", "DAG-40:0-FA18:0_22:0", "DAG-40:0-FA20:0_20:0", "DAG-40:1-FA16:0_24:1", "DAG-40:1-FA16:1_24:0", "DAG-40:1-FA18:0_22:1", "DAG-40:1-FA18:1_22:0", "DAG-40:1-FA20:1_20:0", "DAG-40:2-FA16:1_24:1", "DAG-40:2-FA18:1_22:1", "DAG-40:2-FA18:2_22:0", "DAG-40:2-FA20:1_20:1", "DAG-40:2-FA20:2_20:0", "DAG-40:3-FA18:2_22:1", "DAG-40:3-FA18:3_22:0", "DAG-40:3-FA20:2_20:1", "DAG-40:4-FA18:3_22:1", "DAG-40:4-FA20:2_20:2", "DAG-44:0-FA20:0_24:0", "DAG-44:0-FA22:0_22:0", "DAG-44:1-FA20:0_24:1", "DAG-44:1-FA20:1_24:0", "DAG-44:1-FA22:1_22:0", "DAG-44:2-FA20:0_24:2", "DAG-44:2-FA20:1_24:1", "DAG-44:2-FA20:2_24:0", "DAG-44:2-FA22:1_22:1", "DAG-44:3-FA20:2_24:1", "DAG-46:0-FA22:0_24:0", "DAG-46:1-FA22:0_24:1", "DAG-46:1-FA22:1_24:0", "DAG-46:2-FA22:1_24:1", "DAG-48:0-FA24:0_24:0", "DAG-48:1-FA24:1_24:0", "DAG-48:2-FA24:1_24:1")]

seed_neutrals_TAG_species <- seed_neutrals_TAG[component_name %in% c("TAG-50:3-FA18:3_(18:3/16:0/16:0)", "TAG-50:2-FA18:2_(18:2/16:0/16:0)", "TAG-50:1-FA18:1_(18:1/16:0/16:0)", "TAG-52:6-FA16:0_(16:0/18:3/18:3)", "TAG-52:5-FA16:0_(16:0/18:2/18:3)", "TAG-52:4-FA18:2_(18:2/18:2/16:0)", "TAG-52:4-FA18:1_(18:1/18:3/16:0)", "TAG-52:3-FA16:0_(16:0/18:1/18:2)", "TAG-52:2-FA16:0_(16:0/18:1/18:1)", "TAG-52:2-FA18:0_(18:0/16:0/18:2)", "TAG-54:8-FA18:2_(18:2/18:3/18:3)", "TAG-54:7-FA18:2_(18:2/18:2/18:3)", "TAG-54:7-FA18:1_(18:1/18:3/18:3)", "TAG-54:6-FA18:1_(18:1/18:2/18:3)", "TAG-54:6-FA18:0_(18:0/18:3/18:3)", "TAG-54:6-FA18:2_(18:2/18:2/18:2)", "TAG-54:5-FA18:3_(18:3/18:1/18:1)", "TAG-54:5-FA18:2_(18:2/18:2/18:1)", "TAG-54:5-FA18:0_(18:0/18:2/18:3)", "TAG-54:4-FA18:3_(18:3/20:1/16:0)", "TAG-54:4-FA18:2_(18:2/18:1/18:1)", "TAG-54:4-FA18:0_(18:0/18:2/18:2)", "TAG-54:3-FA18:1_(18:1/18:1/18:1)", "TAG-54:3-FA18:0_(18:0/18:1/18:2)", "TAG-54:2-FA18:1_(18:1/18:1/18:0)", "TAG-56:5-FA18:2_(18:2/18:2/20:1)", "TAG-56:5-FA18:1_(18:1/18:3/20:1)", "TAG-56:4-FA18:3_(18:3/22:1/16:0)", "TAG-56:4-FA20:1_(20:1/18:2/18:1)", "TAG-56:3-FA20:1_(20:1/18:1/18:1)", "TAG-56:2-FA20:1_(20:1/18:0/18:1)", "TAG-58:4-FA22:1_(22:1/18:2/18:1)", "TAG-58:3-FA18:2_(18:2/22:1/18:0)", "TAG-58:3-FA22:1_(22:1/18:1/18:1)", "TAG-58:2-FA18:1_(18:1/22:1/18:0)", "TAG-60:4-FA18:1_(18:1/18:2/24:1)", "TAG-62:3-FA18:1_(18:1/22:1/22:1)", "TAG-58:6-FA18:3_(18:3/22:1/18:2)", "TAG-58:5-FA18:2_(18:2/18:2/22:1)", "TAG-58:5-FA18:1_(18:1/18:3/22:1)", "TAG-60:5-FA18:3_(18:3/22:1/20:1)", "TAG-62:5-FA18:3_(18:3/22:1/22:1)", "TAG-62:4-FA18:2_(18:2/22:1/22:1)", "TAG-62:4-FA20:1_(20:1/24:1/18:2)", "TAG-64:5-FA22:1_(22:1/18:3/24:1)", "TAG-64:4-FA22:1_(22:1/18:2/24:1)", "TAG-64:3-FA18:1_(18:1/24:1/22:1)", "TAG-54:3-FA20:1_(20:1/18:2/16:0)", "TAG-56:3-FA22:1_(22:1/18:2/16:0)", "TAG-56:2-FA16:0_(16:0/18:1/22:1)", "TAG-58:3-FA20:1_(20:1/20:1/18:1)", "TAG-60:4-FA22:1_(22:1/18:2/20:1)", "TAG-60:3-FA22:1_(22:1/18:1/20:1)", "TAG-62:3-FA20:1_(20:1/24:1/18:1)", "TAG-56:3-FA18:0_(18:0/18:2/20:1)", "TAG-56:3-FA20:0_(20:0/18:1/18:2)", "TAG-58:4-FA18:3_(18:3/22:1/18:0)", "TAG-58:5-FA20:1_(20:1/20:1/18:3)", "TAG-60:3-FA24:1_(24:1/18:1/18:1)", "TAG-62:5-FA20:1_(20:1/24:1/18:3)", "TAG-64:3-FA22:1_(22:1/22:1/20:1)", "TAG-64:3-FA24:1_(24:1/20:1/20:1)")]

# normalizing against internal standards and tissue weight

setDT(seed_neutrals_MAG)

seed_neutrals_MAG_IS <- seed_neutrals_MAG[component_name == "MAG-19:2"]

setDT(seed_neutrals_MAG_species); setDT(seed_neutrals_MAG_IS)

seed_neutrals_MAG_species[seed_neutrals_MAG_IS, nmol_mg := (20 * area) * (1 / i.area) * (1 / tissue_wt), on = "sample_no"]

setDT(seed_neutrals_DAG)

seed_neutrals_DAG_IS <- seed_neutrals_DAG[component_name == "DAG-35:1d5-FA17:0_18:1"]

setDT(seed_neutrals_DAG_species); setDT(seed_neutrals_DAG_IS)

seed_neutrals_DAG_species[seed_neutrals_DAG_IS, nmol_mg := (2.442 * area) * (1 / i.area) * (1 / tissue_wt), on = "sample_no"]

seed_neutrals_TAG_IS <- seed_neutrals_TAG[component_name == "TAG-51:2d5-FA16:0 (16:0/19:2/16:0)"]

setDT(seed_neutrals_TAG_species); setDT(seed_neutrals_TAG_IS)

seed_neutrals_TAG_species[seed_neutrals_TAG_IS, nmol_mg := (2.354 * area) * (1 / i.area) * (1 / tissue_wt), on = "sample_no"]

# summing molecular species

seed_neutrals_MAG_sum <- seed_neutrals_MAG_species[, .(sum_nmol_mg = sum(nmol_mg)), by = .(tissue, sample_no)]

seed_neutrals_DAG_sum <- seed_neutrals_DAG_species[, .(sum_nmol_mg = sum(nmol_mg)), by = .(tissue, sample_no)]

seed_neutrals_TAG_sum <- seed_neutrals_TAG_species[, .(sum_nmol_mg = sum(nmol_mg)), by = .(tissue, sample_no)]

# calculating average and standard deviation for summed and individual molecular species

setDT(seed_neutrals_MAG_sum)

seed_neutrals_MAG_sum_avgSD <- seed_neutrals_MAG_sum[, .(avg_nmol_mg = mean(sum_nmol_mg), SD_nmol_mg = sd(sum_nmol_mg)), by = tissue]

seed_neutrals_MAG_species_avgSD <- seed_neutrals_MAG_species[, .(avg_nmol_mg = mean(nmol_mg), SD_nmol_mg = sd(nmol_mg)), by = .(tissue, component_name)]

setDT(seed_neutrals_DAG_sum)

seed_neutrals_DAG_sum_avgSD <- seed_neutrals_DAG_sum[, .(avg_nmol_mg = mean(sum_nmol_mg), SD_nmol_mg = sd(sum_nmol_mg)), by = tissue]

seed_neutrals_DAG_species_avgSD <- seed_neutrals_DAG_species[, .(avg_nmol_mg = mean(nmol_mg), SD_nmol_mg = sd(nmol_mg)), by = .(tissue, component_name)]

setDT(seed_neutrals_TAG_sum)

seed_neutrals_TAG_sum_avgSD <- seed_neutrals_TAG_sum[, .(avg_nmol_mg = mean(sum_nmol_mg), SD_nmol_mg = sd(sum_nmol_mg)), by = tissue]

seed_neutrals_TAG_species_avgSD <- seed_neutrals_TAG_species[, .(avg_nmol_mg = mean(nmol_mg), SD_nmol_mg = sd(nmol_mg)), by = .(tissue, component_name)]

# writing final data files (both sum and individual species)

write.table(seed_neutrals_MAG_sum_avgSD, file = "seed_neutrals_MAG_sum_avgSD.txt", quote = FALSE, row.names = FALSE, sep = "\t")

write.table(seed_neutrals_MAG_species_avgSD, file = "seed_neutrals_MAG_species_avgSD.txt", quote = FALSE, row.names = FALSE, sep = "\t")

write.table(seed_neutrals_DAG_sum_avgSD, file = "seed_neutrals_DAG_sum_avgSD.txt", quote = FALSE, row.names = FALSE, sep = "\t")

write.table(seed_neutrals_DAG_species_avgSD, file = "seed_neutrals_DAG_species_avgSD.txt", quote = FALSE, row.names = FALSE, sep = "\t")

write.table(seed_neutrals_TAG_sum_avgSD, file = "seed_neutrals_TAG_sum_avgSD.txt", quote = FALSE, row.names = FALSE, sep = "\t")

write.table(seed_neutrals_TAG_species_avgSD, file = "seed_neutrals_TAG_species_avgSD.txt", quote = FALSE, row.names = FALSE, sep = "\t")

# plotting avg summed classes with SD error bars

# MAG

ggplot(seed_neutrals_MAG_sum_avgSD) +

geom_col(mapping = aes(tissue, avg_nmol_mg)) +

scale_x_discrete(limits = c("14 DAP", "17 DAP", "20 DAP", "23 DAP", "mature", "germ")) +

geom_errorbar(mapping = aes(tissue, ymin = avg_nmol_mg - SD_nmol_mg, ymax = avg_nmol_mg + SD_nmol_mg), width = 0.2) +

labs(title = "Avg sum of MAG", x = "tissue", y = "nmol / mg") +

theme_minimal() +

theme(plot.title = element_text(hjust = 0.5, size = 20), axis.title = element_text(size = 20), axis.text = element_text(size = 20), axis.line = element_line(color = "black"), panel.grid.major = element_line(linetype = 0), panel.grid.minor = element_line(linetype = 0), axis.ticks = element_line(linetype = 1)) +

scale_y_continuous(expand = c(0, 0), limits = c(0, NA))

ggsave("avg_MAG_sum.svg", width = 10, height = 8, units = "in", dpi = 300)

# DAG

ggplot(seed_neutrals_DAG_sum_avgSD) +

geom_col(mapping = aes(tissue, avg_nmol_mg)) +

scale_x_discrete(limits = c("14 DAP", "17 DAP", "20 DAP", "23 DAP", "mature", "germ")) +

geom_errorbar(mapping = aes(tissue, ymin = avg_nmol_mg - SD_nmol_mg, ymax = avg_nmol_mg + SD_nmol_mg), width = 0.2) +

labs(title = "Avg sum of DAG", x = "tissue", y = "nmol / mg") +

theme_minimal() +

theme(plot.title = element_text(hjust = 0.5, size = 20), axis.title = element_text(size = 20), axis.text = element_text(size = 20), axis.line = element_line(color = "black"), panel.grid.major = element_line(linetype = 0), panel.grid.minor = element_line(linetype = 0), axis.ticks = element_line(linetype = 1)) +

scale_y_continuous(expand = c(0, 0), limits = c(0, NA))

ggsave("avg_DAG_sum.svg", width = 10, height = 8, units = "in", dpi = 300)

# TAG

ggplot(seed_neutrals_TAG_sum_avgSD) +

geom_col(mapping = aes(tissue, avg_nmol_mg)) +

scale_x_discrete(limits = c("14 DAP", "17 DAP", "20 DAP", "23 DAP", "mature", "germ")) +

geom_errorbar(mapping = aes(tissue, ymin = avg_nmol_mg - SD_nmol_mg, ymax = avg_nmol_mg + SD_nmol_mg), width = 0.2) +

labs(title = "Avg sum of TAG", x = "tissue", y = "nmol / mg") +

theme_minimal() +

theme(plot.title = element_text(hjust = 0.5, size = 20), axis.title = element_text(size = 20), axis.text = element_text(size = 20), axis.line = element_line(color = "black"), panel.grid.major = element_line(linetype = 0), panel.grid.minor = element_line(linetype = 0), axis.ticks = element_line(linetype = 1)) +

scale_y_continuous(expand = c(0, 0), limits = c(0, NA))

ggsave("avg_TAG_sum.svg", width = 10, height = 8, units = "in", dpi = 300)

# plotting avg molecular species with SD error bars

# MAG

ggplot(seed_neutrals_MAG_species_avgSD, aes(fill = factor(tissue, levels = c("14 DAP", "17 DAP", "20 DAP", "23 DAP", "mature", "germ")), x = component_name, y = avg_nmol_mg)) +

geom_col(position = "dodge") +

geom_errorbar(mapping = aes(ymin = avg_nmol_mg - SD_nmol_mg, ymax = avg_nmol_mg + SD_nmol_mg), position = "dodge") +

theme_minimal() +

theme(axis.text.x = element_text(angle = -90, vjust = 0.5, hjust = 1, size = 20), plot.title = element_text(hjust = 0.5, size = 20), axis.title = element_text(size = 20), axis.text.y = element_text(size = 20), legend.text = element_text(size = 20), legend.title = element_text(size = 20)) +

labs(title = "MAG molecular species", x = "MAG molecular species", y = "nmol / mg", fill = "tissue")

ggsave("avg_MAG_species.svg", width = 20, height = 10, units = "in", dpi = 300)

# DAG

ggplot(seed_neutrals_DAG_species_avgSD, aes(fill = factor(tissue, levels = c("14 DAP", "17 DAP", "20 DAP", "23 DAP", "mature", "germ")), x = component_name, y = avg_nmol_mg)) +

geom_col(position = "dodge") +

geom_errorbar(mapping = aes(ymin = avg_nmol_mg - SD_nmol_mg, ymax = avg_nmol_mg + SD_nmol_mg), position = "dodge") +

theme_minimal() +

theme(axis.text.x = element_text(angle = -90, vjust = 0.5, hjust = 1, size = 10), plot.title = element_text(hjust = 0.5, size = 20), axis.title = element_text(size = 20), axis.text.y = element_text(size = 20), legend.text = element_text(size = 20), legend.title = element_text(size = 20)) +

labs(title = "DAG molecular species", x = "DAG molecular species", y = "nmol / mg", fill = "tissue")

ggsave("avg_DAG_species.svg", width = 20, height = 10, units = "in", dpi = 300)

# TAG

ggplot(seed_neutrals_TAG_species_avgSD, aes(fill = factor(tissue, levels = c("14 DAP", "17 DAP", "20 DAP", "23 DAP", "mature", "germ")), x = component_name, y = avg_nmol_mg)) +

geom_col(position = "dodge") +

geom_errorbar(mapping = aes(ymin = avg_nmol_mg - SD_nmol_mg, ymax = avg_nmol_mg + SD_nmol_mg), position = "dodge") +

theme_minimal() +

theme(axis.text.x = element_text(angle = -90, vjust = 0.5, hjust = 1, size = 10), plot.title = element_text(hjust = 0.5, size = 20), axis.title = element_text(size = 20), axis.text.y = element_text(size = 20), legend.text = element_text(size = 20), legend.title = element_text(size = 20)) +

labs(title = "TAG molecular species", x = "TAG molecular species", y = "nmol / mg", fill = "tissue")

ggsave("avg_TAG_species.svg", width = 20, height = 10, units = "in", dpi = 300)

# creating Metaboanalyst-compatible data table

sample_no <- data.table("tissue" = c("14 DAP", "17 DAP", "20 DAP", "23 DAP", "mature", "germ"), "label" = c(0,1,2,3,4,5))

# TAG

seed_neutrals_TAG_species[, sample := do.call(paste, c(.SD, sep = "_")), .SDcols = c("tissue", "sample_no")]

seed_neutrals_TAG_species <- seed_neutrals_TAG_species[sample_no, on = .(tissue), nomatch = 0]

seed_neutrals_TAG_metaboanalyst <- dcast(seed_neutrals_TAG_species, sample + label ~ component_name, value.var = "nmol_mg")

setorder(seed_neutrals_TAG_metaboanalyst, label)

write.table(seed_neutrals_TAG_metaboanalyst, file = "seed_neutrals_TAG_metaboanalyst.txt", quote = FALSE, row.names = FALSE, sep = "\t")

# DAG

seed_neutrals_DAG_species[, sample := do.call(paste, c(.SD, sep = "_")), .SDcols = c("tissue", "sample_no")]

seed_neutrals_DAG_species <- seed_neutrals_DAG_species[sample_no, on = .(tissue), nomatch = 0]

seed_neutrals_DAG_metaboanalyst <- dcast(seed_neutrals_DAG_species, sample + label ~ component_name, value.var = "nmol_mg")

setorder(seed_neutrals_DAG_metaboanalyst, label)

write.table(seed_neutrals_DAG_metaboanalyst, file = "seed_neutrals_DAG_metaboanalyst.txt", quote = FALSE, row.names = FALSE, sep = "\t")

# MAG

seed_neutrals_MAG_species[, sample := do.call(paste, c(.SD, sep = "_")), .SDcols = c("tissue", "sample_no")]

seed_neutrals_MAG_species <- seed_neutrals_MAG_species[sample_no, on = .(tissue), nomatch = 0]

seed_neutrals_MAG_metaboanalyst <- dcast(seed_neutrals_MAG_species, sample + label ~ component_name, value.var = "nmol_mg")

setorder(seed_neutrals_MAG_metaboanalyst, label)

write.table(seed_neutrals_MAG_metaboanalyst, file = "seed_neutrals_MAG_metaboanalyst.txt", quote = FALSE, row.names = FALSE, sep = "\t")
